# Comparative transcriptome analysis of *Gastrodia elata* (Orchidaceae) in response to fungus symbiosis to identify gastrodin biosynthesis-related genes

**DOI:** 10.1186/s12864-016-2508-6

**Published:** 2016-03-09

**Authors:** Chi-Chu Tsai, Keh-Ming Wu, Tzen-Yuh Chiang, Chun-Yen Huang, Chang-Hung Chou, Shu-Ju Li, Yu-Chung Chiang

**Affiliations:** Crop Improvement Division, Kaohsiung District Agricultural Improvement Station, Pingtung, 900 Taiwan; Graduate Institute of Biotechnology, National Pingtung University of Science and Technology, Pingtung, 912 Taiwan; Welgene Biotech. Co., Ltd., Taipei, 115 Taiwan; Department of Life Science, Cheng-Kung University, Tainan, 701 Taiwan; Research Center for Biodiversity, China Medical University, Taichung, 404 Taiwan; Department of Biological Sciences, National Sun Yat-sen University, Kaohsiung, 804 Taiwan; Department of Biomedical Science and Environment Biology, Kaohsiung Medical University, Kaohsiung, 807 Taiwan

**Keywords:** Gastrodin, Deep sequencing, Gene regulation, Monooxygenase, Glycosyltransferase

## Abstract

**Background:**

*Gastrodia elata* Blume (Orchidaceae) is an important Chinese medicine with several functional components. In the life cycle of *G. elata*, the orchid develops a symbiotic relationship with two compatible mycorrhizal fungi *Mycena* spp. and *Armillaria mellea* during seed germination to form vegetative propagation corm and vegetative growth to develop tubers, respectively. Gastrodin (p-hydroxymethylphenol-beta-D-glucoside) is the most important functional component in *G. elata*, and gastrodin significantly increases from vegetative propagation corms to tubers. To address the gene regulation mechanism in gastrodin biosynthesis in *G. elata*, a comparative analysis of *de novo* transcriptome sequencing among the vegetative propagation corms and tubers of *G. elata* and *A. mellea* was conducted using deep sequencing.

**Results:**

Transcriptome comparison between the vegetative propagation corms and juvenile tubers of *G. elata* revealed 703 differentially expressed unigenes, of which 298 and 405 unigenes were, respectively up-regulated (fold-change ≥ 2, *q*-value < 0.05, the trimmed mean of M-values (TMM)-normalized fragments per kilobase of transcript per Million mapped reads (FPKM) > 10) and down-regulated (fold-change ≤ 0.5, *q*-value <0.05, TMM-normalized FPKM > 10) in juvenile tubers. After Gene Ontology (GO) annotation and Kyoto Encyclopedia of Genes and Genomes (KEGG) pathway analysis, 112 up-regulated unigenes with KEGG Ortholog identifiers (KOids) or enzyme commission (EC) numbers were assigned to 159 isogroups involved in seventy-eight different pathways, and 132 down-regulated unigenes with KOids or EC numbers were assigned to 168 isogroups, involved in eighty different pathways. The analysis of the isogroup genes from all pathways revealed that the two unigenes TRINITY_DN54282_c0_g1 (putative monooxygenases) and TRINITY_DN50323_c0_g1 (putative glycosyltransferases) might participate in hydroxylation and glucosylation in the gastrodin biosynthetic pathway.

**Conclusions:**

The gene expression of the two unique unigenes encoding monooxygenase and glycosyltransferase significantly increases from vegetative propagation corms to tubers, and the molecular basis of gastrodin biosynthesis in the tubers of *G. elata* is proposed.

**Electronic supplementary material:**

The online version of this article (doi:10.1186/s12864-016-2508-6) contains supplementary material, which is available to authorized users.

## Background

**Fig. 1:**
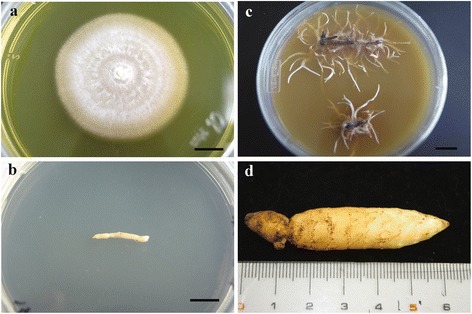
Materials in the study. Fungus of *Mycena dendrobii* (**a**), vegetative propagation corm (**b**), *Armillaria mellea* (**c**), and juvenile tuber (**d**) of *Gastrodia elata*. Scale bars = 1 cm

*Gastrodia elata* is a rootless and leafless achlorophyllous orchid that grows in a symbiotic relationship with two compatible mycorrhizal fungi, *Mycena* spp. and *Armillaria mellea*, during seed germination and vegetative growth, respectively [1–3]. The seeds of *G. elata* are tiny and do not possess an endosperm, and these seeds germinate only when adequate nutrition is obtained through the digestion of the specific fungi, *Mycena* spp., which invades the embryonic cells of these seeds [4–9]. Currently, four fungi species, including *Mycena anoectochila*, *M. dendrobii* (Fig. 1a), *M. orchidicola*, and *M. osmundicola*, isolated from different species of orchids [10], promote the germination of *G. elata* seeds to form protocorms and further develop into vegetative propagation corms (Fig. 1b) [11–16]. Once vegetative propagation corms have been established from seed germination, *G. elata* undergoes vegetative growth through an established symbiotic association with the compatible mycorrhizal fungi, *A. mellea* (Fig. 1c), to yield juvenile tubers (Fig. 1d) [1, 6, 17]. The vegetative propagation corms of *G. elata* obtain nutrition and energy from *A. mellea* to develop into tubers, and the growth conditions of tubers are positively and closely associated with the hyphal development of this fungi [1, 9, 18, 19]. The hyphae of *A. mellea* develop well in the cortical layers of *G. elata* tubers [1, 20-22]; however, the cells in the pith of tubers digest the invaded hyphae to obtain nutrition and energy [1].

Since ancient times, *Gastrodia elata* has been used as a Chinese medicine for the cure of various conditions, including for its analgesic, antiepileptic, neuroprotective, anticonvulsant, and sedative effects against vertigo, general paralysis, and tetanus [23–26]. Numerous functional components isolated from *G. elata* have been characterized, such as gastrodin (4-hydroxymethylphenyl-β-D-glucopyranoside) and the aglycone gastrodigenin (4-hydroxybenzyl alcohol) as a primary active ingredient [27–29], and the other related components, including 4-hydroxybenzaldehyde, vanillyl alcohol, and vanillin, also show potential anticonvulsant activity [30–36]. Moreover, other constituents in *G. elata*, including gastrodioside, 4-hydroxybenzyl methyl ether, 4-hydroxybenzoic acid, parishin, β-Sitosterol, bis (4-hydroxybenzyl) sulfide, N6-(4-hydroxybenzyl) adenine riboside, dauricine, citric acid, palmitate, and succinic acid, have been reported [37–39].

Gastrodin, a simple glycoside comprising glucose and 4-hydroxybenzyl alcohol (the precursor of gastrodin), is the major phenolic compound of *G. elata*, and pharmacological tests have shown that this compound exhibits tranquilization, anti-inflammation, analgesia, cortical neuron protection, memory improvement, sedative, anticonvulsant, free radical scavenging, neuroprotective effect, anesthetic, and antioxidant effects [40–42]. Gastrodin was identified, characterized, and artificially synthesized at the end of 1970s [43]. Furthermore, gastrodin biosynthesis markedly increases from the growth stage of vegetative propagation corms to that of juvenile tubers, which have no flower buds [44]. In general, gastrodin production is derived from 4-hydroxybenzyl alcohol through a one-step glycosylation with different glucose donors. Therefore, one of key enzymes of gastrodin biosynthesis is glucosyltransferase, a large family identified in various plants [45]. Glycosylation is typically the final step in the biosynthesis of secondary plant compounds, resulting in the formation of a large number of glucosides [45–48]. The glycosylation might increase solubility or decrease volatility compared with non-glycosylated molecules [47]. Toluenes are general components in plants that serve as the precursors of plant secondary compounds [49–52]. Toluene is considered as a precursor of gastrodin [53]. The derivation of the metabolic pathway of 4-hydroxybenzyl alcohol derived from toluene is largely unknown in *G. elata* and other plants. However, the catalytic pathway from toluene to 4-hydroxybenzyl alcohol has been reported to involve hydroxylation through monooxygenase of cytochrome P450 (CYP450) [54, 55], a member of a large enzyme family in plants that catalyzes most of the oxidation steps in plant secondary metabolism [56–58]. The molecular basis of gastrodin biosynthesis remains largely unknown. In the present study, the comparative transcriptome analysis among *A. mellea*, the vegetative propagation corms and juvenile tubers of gastrodia was conducted using deep sequencing to reveal the gene regulation of gastrodin biosynthesis in *G. elata*.

## Results and discussion

### The transcriptome sequencing of NGS

*De novo* transcriptome sequencing has been used in various functional genomics studies, and is particularly suitable for gene expression profiling in non-model organisms without genomic sequences. The next generation sequencing (NGS) technology not only provides a comparative expressed sequence tag (EST) analysis for gene discovery on a genome-wide scale in non-model plants but also an efficient process for transcriptome sequencing and characterization. NGS platforms, such as the Illumina/Solexa Genome Analyzer and the Roche 454 GS FLX, have been widely used in recent years for the high-throughput sequencing of various organisms [59, 60]. Using these techniques for *de novo* transcriptome sequencing, EST databases have been successfully obtained for several medicinal herbs, including American ginseng [48], *Salvia miltiorrhiza* [61], sweet wormwood [62], *Euphorbia fischeriana* [63], *Taxus* [64], and other crops, such as chili pepper [65], maize [66], *Curcuma longa* [67], chestnut [68], *Eucalyptus* tree [69], olive [70], *Camellia sinensis* [71], sweet potato [72], *Arabidopsis* [73], and *Phalaenopsis* [74]. The Illumina platform is beneficial and useful for gene discovery because this technique can obtain deeper coverage and higher accuracy than Roche 454 sequencing technology [74]. Hence, the Illumina system was used in the present study to clarify the differential gene expression of different life stages of *G. elata*.

### Sequencing and *de novo* assembly

**Fig. 2:**
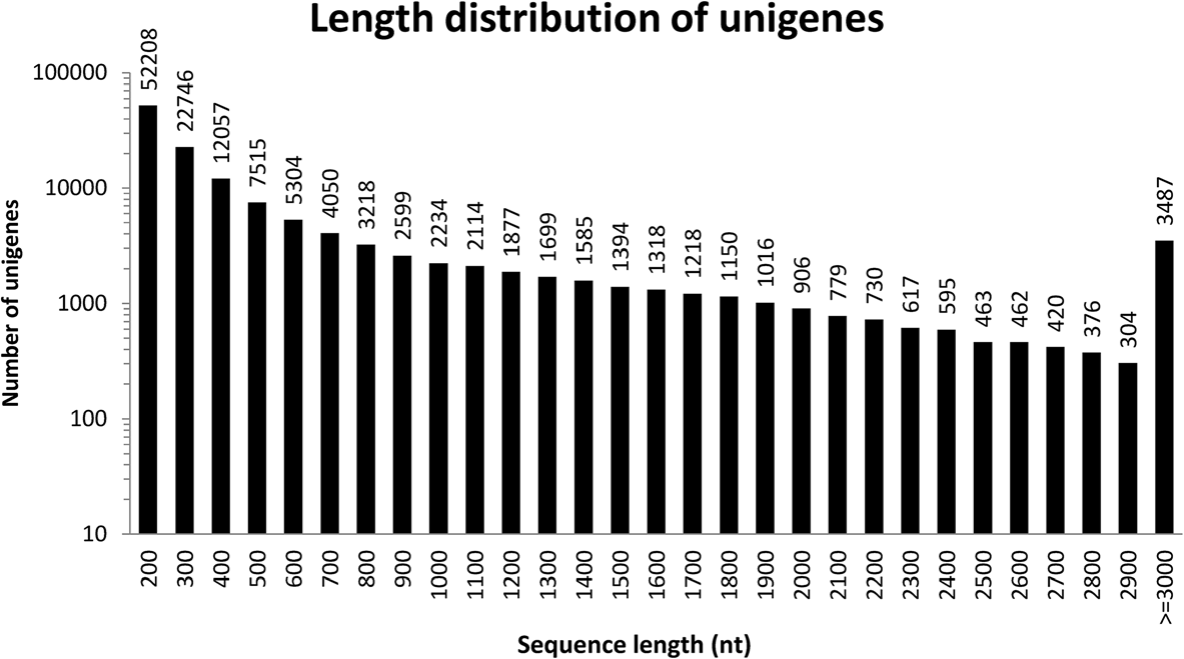
Summary distribution of the lengths of the 134,441 unigenes from combing three samples of raw reads (>200 nt, mean length = 686 nt, N50 = 1184 nt, Min = 201 nt, Max = 19,660 nt)

A total of 21,045,338 (2x75 bases, 51 % GC), 18,436,794 (2x75 bases, 44 % GC) and 18,253,900 (2x75 bases, 45 % GC) high-quality paired-end (PE) reads were generated from Illumina HiSeq2000 platform, and approximately 3 giga bases of sequence data were obtained for each of *A. mellea*, vegetative propagation corm, and juvenile tuber of *G. elata* (Table 1). These short sequence reads have been deposited in NCBI under GEO accession number GSE73633. High-quality bases (above Q20) were more than 97 % for all three samples, indicating an excellent quality (Q20 means 1 error per 100 sequenced bases), while another high quality indicator was the aboundance peak of average sequence quality per read located around Q38 (less than 0.0158 % of error rate) for all three samples. The high-quality PE reads were used for *de novo* transcriptome shotgun assembly (TSA) to build transcript isoforms based on paired-end information. From a total of 161,517 assembled transcript isoforms (≥200 bases), 134,441 transcripts were selected as the representive unigenes with longest length for all loci (i.e., genes). The mapping rates of these high quality reads from all three samples against the total transcripts were all above 86 % (Table 2). The final N50 lengths of 1592 and 1184 bases, and the total lengths of 137,618,051 and 92,177,843 bases were calculated for the transcripts and the unigenes, respectively (Table 2) (Fig. 2). N50 statistics are widely used to assess the quality of the assembly, and the higher the N50 value representing the assembly the better the quality [67]. Compared with other plant transcriptome sequencing of *de novo* TSAs [61, 67, 69, 71, 72, 75–78], the N50 value obtained in the present study was above the average and adequate for further analysis (Table 3).

**Table 1:** Basic Statistics of RNA-Seq generated from *Armillariella mellea*, vegetative propagation corm and juvenile tuber of *Gastrodia elata* encoding by Illumina HiSeq2000 platform

	Sample		
	Vegetative propagation corm	Juvenile tuber^a^	*Armillaria mellea*
Total number of read pairs	18,436,794	18,253,900	21,045,338
Total nucleotides (nt)	2,765,519,100	2,738,085,000	3,156,800,700
GC percentage	44 %	45 %	51 %
≥Q20 percentage	97 %	97 %	97 %
Read length	75	75	75

^a^The tubers of *Gastrodia elata* have established symbiotic associations with *Armillaria mellea*

**Table 2:** Summary of the *de novo* transcriptome shotgun assembly from all Illumina sequences

Assembled transcripts	
Total number of transcripts	161,517
Total bases of transcripts	137,618,051 nt
Longest transcript	19,660 nt
Average transcript length	852 nt
Median transcript length	420 nt
N50 transcript length	1592 nt
Read mapping rate of sample *Armillaria mellea*	88.21 %
Read mapping rate of sample juvenile tuber^a^	88.73 %
Read mapping rate of sample vegetative propagation corm	86.75 %
Assembled unigenes	
Total number of transcripts	134,441
Total bases of unigenes	92,177,843 nt
Longest unigene	19,660 nt
Average unigene length	686 nt
Median unigene length	358 nt
N50 unigene length	1184 nt
Predicted peptides	
Total number of peptides	50,084
Total amino acids of peptides	14,597,170 aa
Longest peptide	5370 aa
Average unigene length	291 aa
Median peptide length	199 aa

*Abbreviations*: *aa* amino acids, *nt* nucleotides

^a^The tubers of *Gastrodia elata* have established symbiotic associations with *Armillaria mellea*

**Table 3:** Comparative analysis of plant transcriptome N50 values

Organisms	N50 (in bases)
*Ipomoea batatas* (Wang et al. [72])	765
*Eucalyptus grandis* (Mizrachi et al. [69])	1640
*Salvia miltiorrhiza* (Hua et al. [61])	533
*Camellia sinensis* (Shi et al. [71])	506
*Cajanus cajan* (Kudapa et al. [76])	1510
*Myrica rubra* (Feng et al. [75])	708
*Capsicum frutescens* (Liu et al. [65])	1108
*Curcuma longa* (Annadurai et al. [67])	1448
*Cuscuta pentagona* (Ranjan et al. [77])	1550
*Hevea brasiliensis* (Salgado et al. [78])	837
Average of above studies	1061
This study	1592

### Functional annotation and Gene Ontology classification

All unigenes were annotated according to the sequence similarity search against NCBI non-redundant protein sequence (nr) database using BLASTX algorithm. A total of 59,932 unique sequences were annotated, accounting for 44.58 % of the total unigenes (Table 4). Gene Ontology (GO) assignment were performed for the functional categorization of the annotated unigenes. A total of 11,645 unigenes were mapped to GO terms, accounting for 8.66 % of the unigenes (Table 4). Because multiple GO terms can be assigned to the same unigene [79], totally 58,488 GO terms were assigned in the present study. The GO annotation showed that these unigenes represent diverse functionalities and are involved in various metabolic pathways. In *A. mellea*, 9101, 7484, and 5694 GO terms, respectively, represent molecular function, biological process and cellular component categories [See Additional file 1: Figure S1]. In the molecular function category, the terms integral to “binding” (GO:0005488) and “catalytic activity” (GO:0003824) were shown as the most frequently occurring, constituting 19.23 % (4285) and 18.56 % (4136) of the level 2 GO terms, respectively. “Metabolic process” (GO:0008152) and “biological regulation” (GO:0065007) were the most frequently occurring under the biological process category, representing 9.50 % (2116) and 6.97 % (1552) of the level 2 GO terms, respectively. In the cellular component category, “cell part” (GO:0044464) was the most frequently occurring, representing 19.20 % (4277) of the total level 2 GO terms.

**Table 4:** Summary statistics of unigenes with functional annotations for all combined assembly and for each sample

Functional annotation	No. of unigene hits	Percentage (%)
All (134,441 unigenes)		
NR	59,932	44.58
GO	11,645	8.66
KEGG	27,008	20.09
Vegetative propagation corm (82,712 unigenes)		
NR	33,638	40.67
GO	7338	8.87
KEGG	15,073	18.22
Juvenile tuber^a^ (71,722 unigenes)		
NR	27,296	38.06
GO	4396	6.13
KEGG	11,301	15.76
*Armillaria mellea* (49,890 unigenes)		
NR	29,401	58.93
GO	3900	7.82
KEGG	13,243	26.54

^a^The tubers of Gastrodia elata have established symbiotic associations with Armillaria mellea

In vegetative propagation corm of *G. elata*, 17,371, 9875, and 6915 GO terms, respectively, represent molecular function, biological process, and cellular component categories [See Additional file 1: Figure S1]. In the molecular function category, the terms integral to “binding” (GO:0005488) and “catalytic activity” (GO:0003824) occurred most frequently, representing 24.84 % (8484) and 20.71 % (7076) of the total level 2 GO terms, respectively. In the biological process category, “metabolic process” (GO:0008152) was the most frequently occurring, representing 11.95 % (4083) of the total level 2 GO terms. In the cellular component category, “cell part” (GO:0044464) was the most frequently observed, representing 15.69 % (5359) of the total level 2 GO terms.

In the juvenile tuber of *G. elata*, 10,259, 5927, and 3753 GO terms were shown for molecular function, biological process and cellular component categories, respectively [See Additional file 1: Figure S1]. In the molecular function category, the terms integral to “binding” (GO:0005488) and “catalytic activity” (GO:0003824) were most frequently observed, representing 27.18 % (5420) and 19.53 % (3895) of the total level 2 GO terms, respectively. Metabolic process (GO:0008152) was the most frequently observed under the biological process category, representing 12.37 % (2466) of the total level 2 GO terms. In the cellular component category, “cell part” (GO:0044464) was the most frequently observed, representing 14.8 % (2951) of the total level 2 GO terms.

### Differential expression analysis between *A. mellea* and juvenile tuber of *Gastrodia elata*

**Fig. 3:**
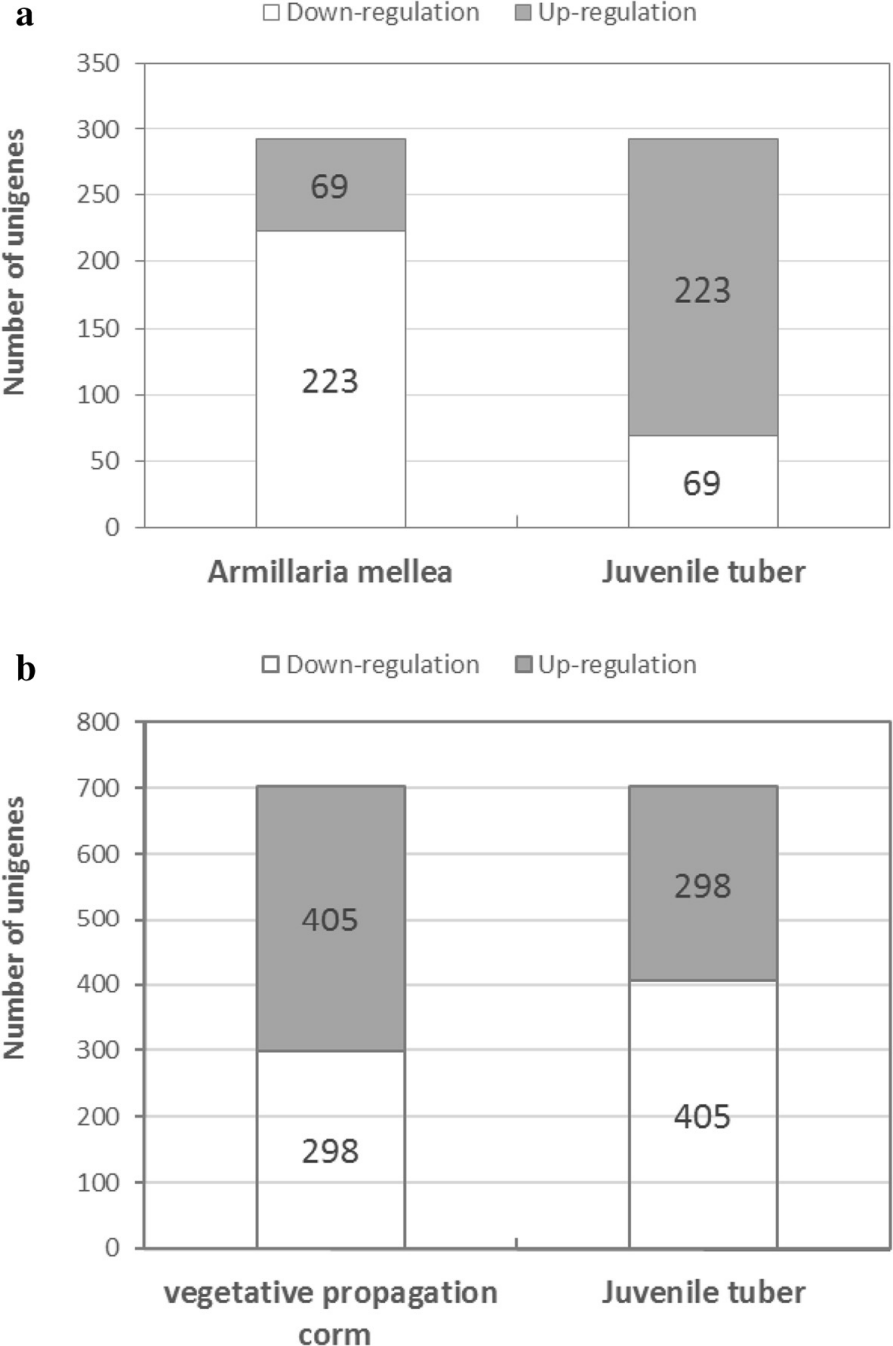
Differentially expressed unigenes between **a** *Armillaria mellea* and juvenile tuber of *Gastrodia elata* (under the criteria: the absolute value of the log2-FC ≥ 1, the *q*-values < 0.05 and the TMM-normalized FPKM > 0.3), and **b** vegetative propagation corm and juvenile tuber (under the criteria: the absolute value of the log2-FC ≥ 1, the *q*-values < 0.05 and the TMM-normalized FPKM > 10). Numbers of up- and down-regulated unigenes were shoen in boxes

The comparative analysis of the transcriptomes of the *A. mellea* and *G. elata* (symbiosis with *A. mellea*) juvenile tubers was conducted based on the combined transcriptome assembly of all three samples. Among the total 134,441 unigenes, 49,890 and 71,722 unigenes were aligned with reads from *A. mellea* and *G. elata* juvenile tubers, respectively [See Additional file 2: Figure S2]. Among these unigenes, only 5547 unigenes were identified in both samples of deep sequencing data. To evaluate differential gene expression, the absolute value of the log2-FC (fold changes) ≥ 1, the *q*-values < 0.05 and the TMM-normalized FPKM > 0.3 were used as the criteria to determine the significance of gene expression differences [80]. A total of 292 differentially expressed unigenes were revealed in the transcriptome comparison, of which sixty-nine unigenes were significantly up-regulated (log2-FC ≥ 1, FPKM > 0.3, *q*-values < 0.05) in *A. mellea* [See Additional file 3: Table S1], and 223 unigenes in *G. elata* juvenile tubers were expressed at significantly higher levels (log2-FC ≤ -1, FPKM > 0.3, *q*-values < 0.05) (Fig. 3a) [See Additional file 4: Table S2]. Of 292 the differentially expressed unigenes, only 106 unigenes can be assigned with KEGG Ortholog identifiers (KOids) or enzyme commission (EC) numbers corresponding to biological pathways for cellular functions and molecular interactions after KEGG analysis. Among these, twenty-five up-regulated unigenes from *A. mellea* and *G. elata* juvenile tuber were assigned KOids or EC numbers corresponding to twenty-eight isogroups involved in sixteen different pathways [See Additional file 5: Table S3]; and eighty-one down-regulated unigenes were corresponding to 134 isogroups involved in sixty different pathways [See Additional file 6: Table S4].

### Differential expression analysis between the juvenile tuber and vegetative propagation corm of *Gastrodia elata*

The comparative analysis of transcriptomes of the juvenile tubers (symbiosis with *A. mellea*) and vegetative propagation corms (asymbiosis with *A. mellea*) was conducted also based on the combined transcriptome assembly of all three samples. Among the total 134,441 unigenes, 71,722 and 82,712 unigenes were aligned with reads from juvenile tubers and vegetative propagation corms, respectively [See Additional file 7: Figure S3]. Among these, 63,317 unigenes were identified in both samples, and 22,942 differentially expressed unigenes were revealed in the transcriptome comparison using the same criteria (the absolute value of the log2-FC ≥ 1, the *q*-values < 0.05 and the TMM-normalized FPKM > 0.3). 7383 and 15,559 unigenes were expressed at higher levels in *G. elata* juvenile tubers and vegetative propagation corms, respectively (data not shown). To focus on highly and differentially expressed unigenes by modifying the threshold of TMM-normalized FPKM larger than ten, 703 highly and differentially expressed unigenes were revealed in the transcriptome comparison. Among which 298 unigenes were significantly up-regulated (log2-FC ≥ 1, *q*-values < 0.05, TMM-normalized FPKM > 10) in juvenile tubers [See Additional file 8: Table S5], and 405 unigenes in vegetative propagation corms were expressed at significantly higher levels (log2-FC ≤ -1, *q*-values < 0.05, TMM-normalized FPKM > 10) (Fig. 3b) [See Additional file 9: Table S6]. Of 703 the differentially expressed genes, only 244 unigenes can be assigned to KOids or EC numbers corresponding to to biological pathways for cellular functions and molecular interactions after KEGG analysis. Among these, 112 up-regulated (log2-FC ≥ 1, *q*-value < 0.05, TMM-normalized FPKM > 10) unigenes from juvenile tubers compared with vegetative propagation corms were assigned to KOids or EC numbers corresponding to 159 isogroups involved in seventy-eight different pathways [See Additional file 10: Table S7]; and 132 down-regulated (log2-FC ≤ -1, *q*-value < 0.05, TMM-normalized FPKM > 10) unigenes were assigned to KOids or EC numbers corresponding to 168 isogroups involved in eighty different pathways [See Additional file 11: Table S8].

Kusano (1911) first reported that *G. elata* existed in a mycorrhizal relationship with the wood-rotting pathogen *A. mellea*; however, this relationship was uncharacterized [18]. Until 1980, Zhang and Li showed that *G. elata* digests the invasive hyphae of *A. mellea* as the source of nutrition [1]. Lan et al. (1986) also confirmed that *A. mellea* was used as the source of nutrition for *G. elata* through the observation of labeled materials from *A. mellea* in the transverse section of *G. elata*. The labeled materials appeared in mitochondria, the endoplasmic reticulum and vacuoles of *G. elata* cortical cells [20]. When the hyphae of *A. mellea* are disconnected between wood (source of nutrition for *A. mellea*) and *G. elata*, the growth of *G. elata* terminates and this organism dies; therefore, the role of *A. mellea* for *G. elata* was considered as the food for survival [21]. According to the differential gene expression in *G. elata* in response to *A. mellea* symbiosis, unigene TRINITY_DN70668_c0_g1 is significantly induced [See Additional file 8: Table S5], as high as ~7 folds, and this gene was annotated as a gastrodianin (i.e., gastrodia antifungal proteins, GAFPs) gene, which digests the cell wall of *A. mellea* [81]. This result suggested that *G. elata* digests the invasive hyphae of *A. mellea* as a source of nutrition according to Zhang and Li (1980) [1]. In the present study, the low-level gene expression of the gastrodianin biosynthetic gene was detected in vegetative propagation corms (i.e., primary corms), which differentiated from protocorms, suggesting that the symbiotic relationship between *A. mellea* and *G. elata* only can be developed during the vegetative propagation corms of *G. elata* [82]. According to previous reports, there are two copies of gastrodianin biosynthetic genes in *G. elata* [81], and these two genes (unigenes TRINITY_DN70668_c0_g1 and TRINITY_DN48867_c0_g1) were also identified through deep sequencing data in the present study, only unigene TRINITY_DN70668_c0_g1 was significantly induced in the juvenile tubers of *G. elata* in response to *A. mellea* symbiosis [See Additional file 8: Table S5]. The result was consistent with the previous gene expression study of the gastrodianin biosynthetic gene in *G. elata* [81].

### Identification and validation of candidate genes involved in gastrodin biosynthesis

**Fig. 4:**
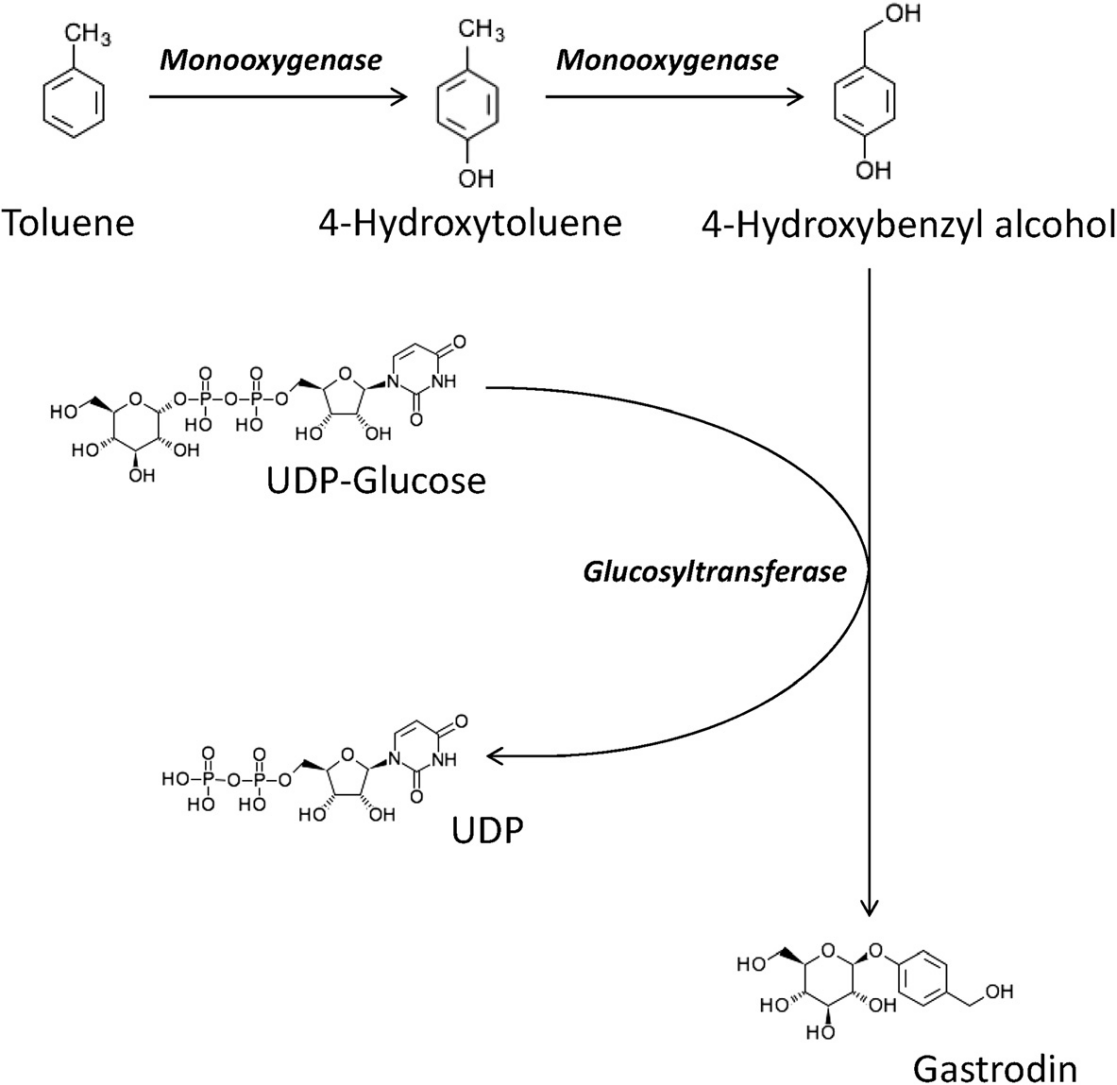
Putative gastrodin biosynthetic pathway in *Gastrodia elata*

The mechanism and related genes in the gastrodin biosynthesis pathway are currently unknown. To the best of our knowledge, gastrodin (4-hydroxymethylphenyl-β-D-glucopyranoside) is a simple glycoside comprising glucose and 4-hydroxybenzyl alcohol [27]. The last biosynthesis enzyme of gastrodin is glucosyltransferase [45]. Gastrodins are synthesized from 4-hydroxybenzyl alcohol with UDP-glucose via glucosylation catalyzed through glucosyltransferase. The precursor of gastrodin, 4-hydroxybenzyl alcohol, is catalyzed through cresols degradation (toluene degradation) from toluene through two steps of hydroxylation via monooxygenase (CYP450) [54, 55]. Therefore, both monooxygenase and glucosyltransferase are considered two key enzymes for gastrodin biosynthesis [53]. According to the chemical structure of gastrodin and gastrodin precursors, analyzed in previous reports, the putative gastrodin biosynthetic pathway is shown in Fig. 4. However, both monooxygenase (CYP450) and glucosyltransferase belong to a large enzyme families involved in different biosynthesis pathways in various plants [45, 54, 55]. Moreover, 4-hydroxybenzyl alcohol and gastrodin were also detected in *Anoectochilus formosanus* [83]. To determine the candidate genes involved in gastrodin biosynthesis, the comparative analysis of the transcriptomes between vegetative propagation corm and juvenile tuber of *G. elata* was conducted. A total of thirty and forty-six unigenes, respectively, were annotated to monooxygenase and glucosyltransferase among the 63,317 unigenes expressed both in the juvenile tubers and the vegetative propagation corms of *G. elata*. Under the criteria of log2-FC ≥ 1, TMM-normalized FPKM > 3 and *q*-values < 0.05, four putative monooxygenases were selected. Among them, unigene TRINITY_DN54282_c0_g1 was the most abundance in FPKMs and differentially expressed one (~2.4 times higher in the juvenile tubers). Under the same criteria, three putative glucosyltransferases were selected and the unigene TRINITY_DN50323_c0_g1 was the highest differentially expressed one (~3.2 times higher in the juvenile tubers).

**Fig. 5:**
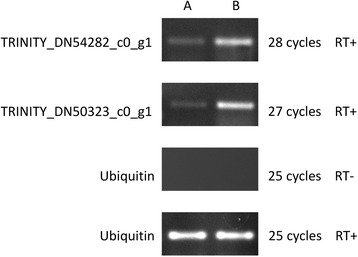
Semi-quantitative RT-PCR profile of gasstrodin related candidate genes unigene TRINITY_DN54282_c0_g1 (monooxygenase) and TRINITY_DN50323_c0_g1 (glycosyltransferase) in different life stage of vegetative propagation corm (**a**) and juvenile tuber (**b**) of *Gastrodia elata* tissues with ubiquitin as the internal control. RT+ and RT- represent amplifications with and without reverse transcriptase

**Fig. 6:**
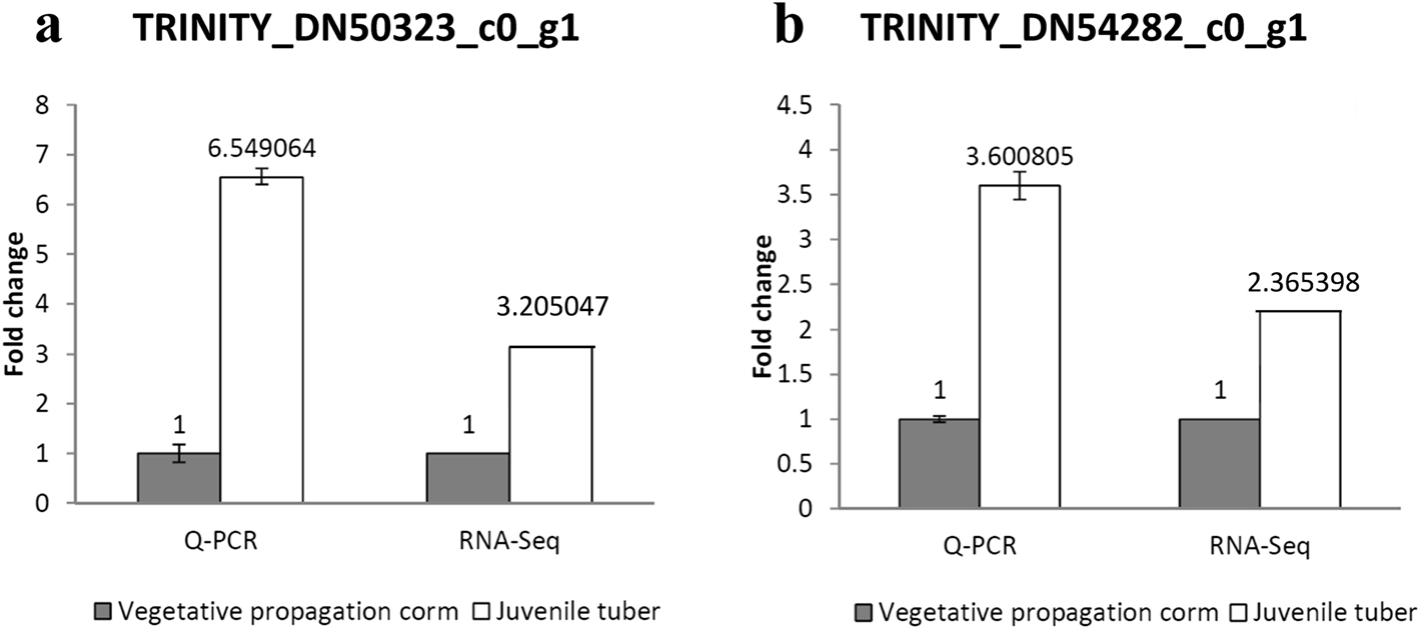
Quantitative real-time PCR (Q-PCR) validations of (**a**) unigene TRINITY_DN50323_c0_g1 and (**b**) unigene TRINITY_DN54282_c0_g1 of RNAseq results (TMM-normalized FPKM fold changes). Comparison of differential expression values between the juvenile tuber (white bar) and vegetative propagation corm (grey bar) of *Gastrodia elata* determined by qRT-PCR and RNAseq

To validate the differential expression of gastrodin biosynthesis-related unigenes TRINITY_DN54282_c0_g1 and TRINITY_DN50323_c0_g1, we investigated the expression of these genes in different life stages between the vegetative propagation corms and juvenile tubers of *G. elata* using semi-quantitative RT-PCR and real-time PCR (quantitative RT-PCR, qRT-PCR), and compared the results with fold-changes of FPKM (RNA-Seq). The specific primers for semi-quantitative RT-PCR and qRT-PCR were designed. In semi-quantitative RT-PCR, expression profiling revealed the differential expression of both monooxygenases and glycosyltransferases between the vegetative propagation corms and juvenile tubers of *Gastrodia elata* (Fig. 5). Both monooxygenases and glycosyltransferase genes were up-regulated in the juvenile tubers and were considered as gastrodin biosynthetic-related genes, as gastrodin production markedly increases from the growth stage of vegetative propagation corms to that of juvenile tubers [44]. In addition, the differential expression of the two genes was also validated through qRT-PCR analysis as shown in Fig. 6. The expression levels of the unigene TRINITY_DN54282_c0_g1 were up-regulated up to 6.5 times in juvenile tubers and 2.4 times for qRT-PCR and RNA-Seq, respectively. Similarly, qRT-PCR and RNA-Seq showed that the expression levels for unigene TRINITY_DN50323_c0_g1 were up-regulated approximately 3.6 and 3.2 times, respectively, in juvenile tubers. The results of both semi-quantitative RT-PCR (Fig. 5) and qRT-PCR analysis (Fig. 6) were consistent with the RNA-Seq data.

**Fig. 7:**
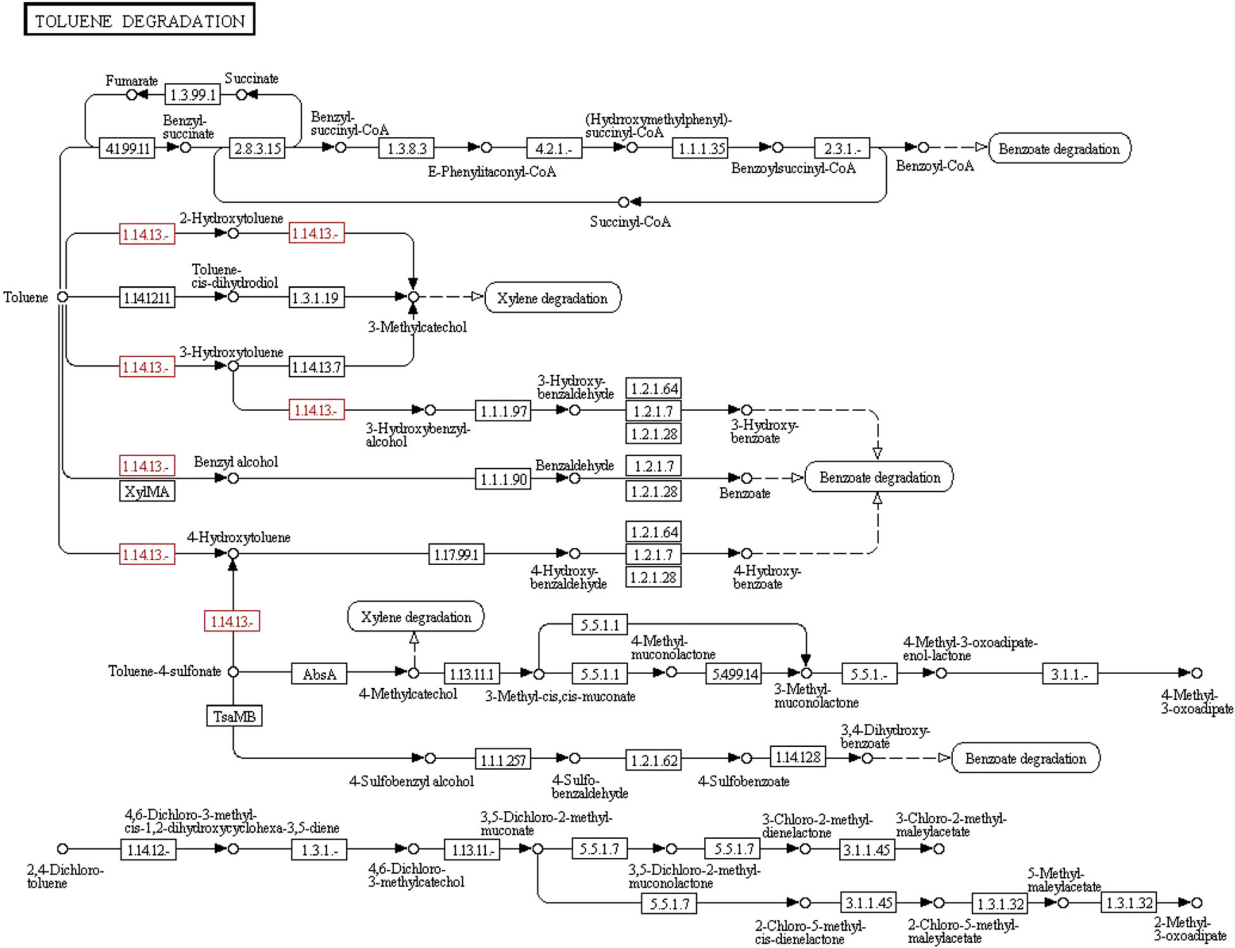
Hydroxylation by monooxygenase (EC:1.14.13.-) in toluene degradation. The putative enzymes are in red

**Fig. 8:**
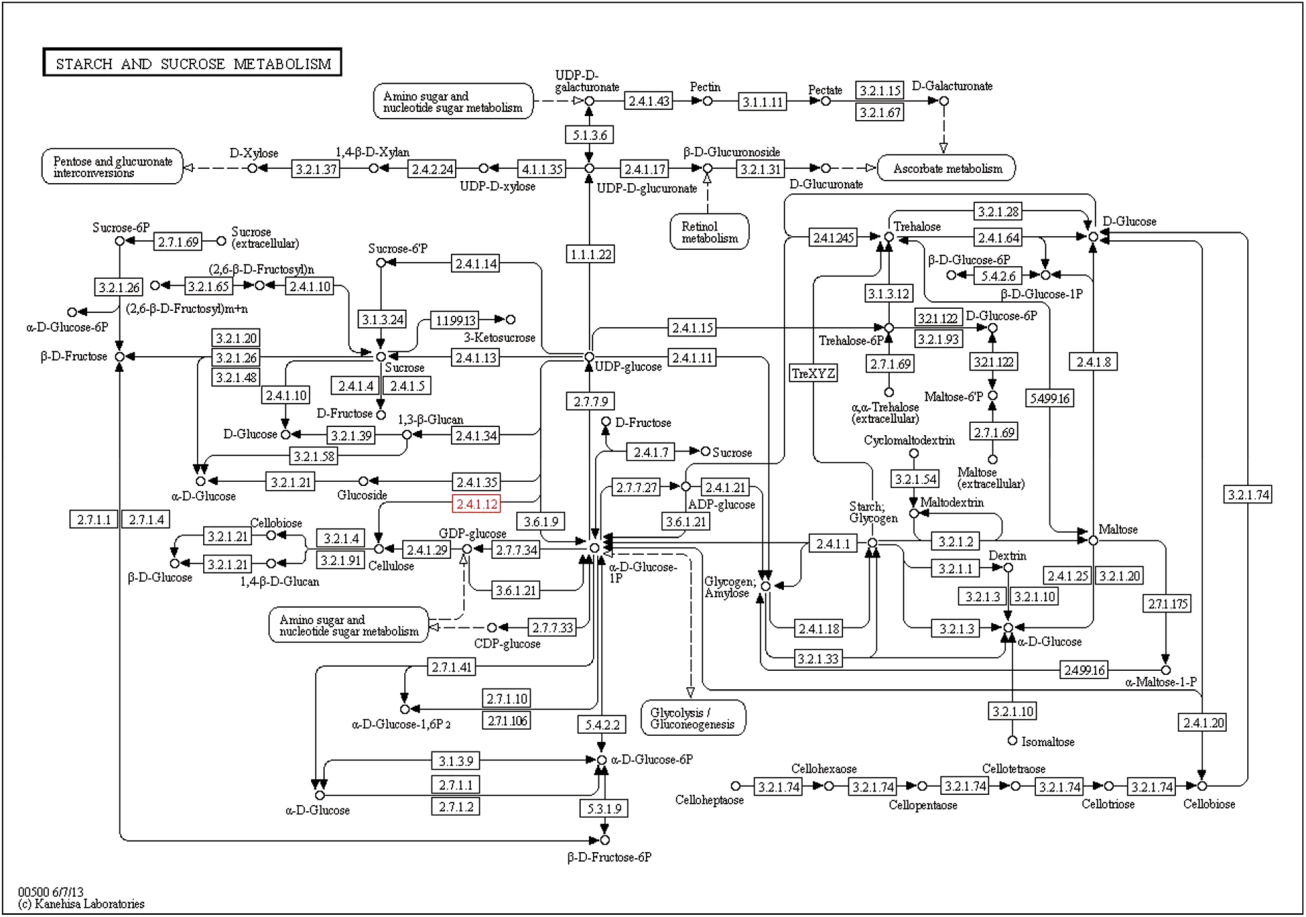
Glucosylation by beta-1,4-glucosyltransferase (EC:2.4.1.12) in starch and sucrose metabolism. The putative enzyme is in red

Monooxygenases belong to cytochrome P450 proteins, the largest family of plant proteins, which catalyze most of the oxidation steps in plant secondary metabolism [57, 58]. The comparison of the chemical structures of 4-hydroxybenzyl alcohol and the precursor toluene (Fig. 4), revealed two steps catalyzed through monooxygenase, the conversion of toluene to 4-hydroxytoluene and the conversion of 4-hydroxytoluene to 4-hydroxybenzyl alcohol. Therefore, unigene TRINITY_DN54282_c0_g1 (EC: 1.14.13.-) was considered as a toluene monooxygenase gene, consistent with the KEGG pathway annotation (Fig. 7). Generally, glycosylation is the last step in the biological biosynthesis of secondary metabolism because sugar conjunction results in both the increased water solubility and stability of the compounds [84–86]. In *G. elata*, glycosyltransferase catalyzes the last step of the gastrodin biosynthesis pathway, which converts 4-hydroxybenzyl alcohol to gastrodin [45] (Fig. 4). Therefore, unigene TRINITY_DN50323_c0_g1 (EC: 2.4.1.12) was considered as a glycosyltransferase gene, consistent with the KEGG pathway annotation (Fig. 8). In short, both unigenes TRINITY_DN54282_c0_g1 and TRINITY_DN50323_c0_g1 might be key enzyme genes that, respectively, participate in the hydroxylation (Fig. 7) and glucosylation (Fig. 8) of gastrodin (Fig. 4).

### Cloning of gastrodin biosynthesis related genes

**Fig. 9:**
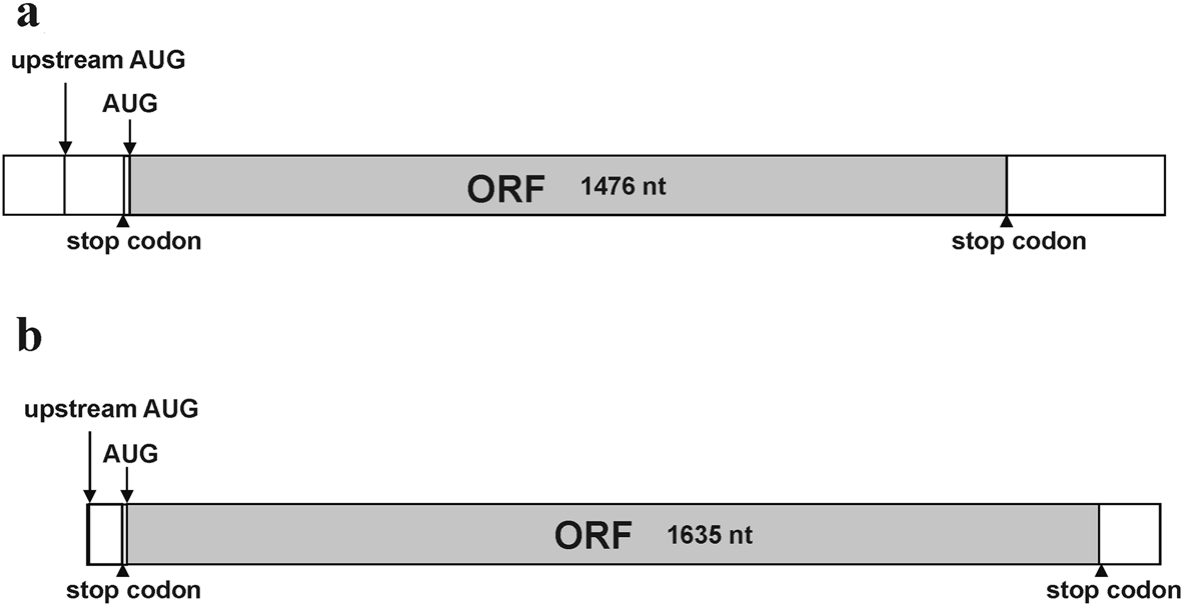
**a** Schematic representation of the mRNA transcripts of (**a**) unigene TRINITY_DN54282_c0_g1 (monooxygenase) with an out-of-frame AUG and an in-frame stop codon upstream the start codon; **b** unigene TRINITY_DN50323_c0_g1 (glycosyltransferase) with an out-of-frame AUG and an in-frame stop codon upstream the start codon

The full-length cDNA sequences of monooxygenases (unigene TRINITY_DN54282_c0_g1) and glycosyltransferases (unigene TRINITY_DN50323_c0_g1) from *G. elata* were further isolated through RACE analysis. The nucleotide sequence of the full-length monooxygenase cDNA has an open reading frame (ORF) of 1476 nucleotides spanning from the first initiation codon (ATG) to the termination codon (TGA), an in-frame stop codon located 12 nt upstream from the initiation codon and an out-of-frame ATG located upstream of the main ORF. The complete ORF encodes a protein of 491 amino acids with a predicted molecular mass of 55.8 kDa (Fig. 9a). In addition, the nucleotide sequence of the full-length glucosyltransferase cDNA has an ORF of 1635 nucleotides spanning from the first initiation codon ATG to the termination codon TGA, an in-frame stop codon located 12 nt upstream from the initiation codon and an out-of-frame ATG located upstream of the main ORF. The complete ORF encodes a protein of 544 amino acids with a predicted protein of 63.1 kDa (Fig. 9b).

Notably, both monooxygenases (unigene TRINITY_DN54282_c0_g1) and glycosyltransferases (unigene TRINITY_DN50323_c0_g1) induced in response to fungi symbiosis possess an out-of-frame upstream ATG and an in-frame stop codon in the main ORF within the 5’UTR. In mammals, upstream ATGs/upstream ORFs significantly reduce protein expression levels through a reduction of the translation efficiency [87, 88] or mRNA decay [89–91]. Upstream AUGs/upstream ORFs in the 5′ UTR efficiently disrupt the translation of the downstream coding sequence, thereby reducing the translation efficiency of the main coding region [87, 88]. According to deep sequencing, semi-quantitative RT-PCR, and real-time RT-PCR, both monooxygenases (unigene TRINITY_DN54282_c0_g1) and glycosyltransferase (unigene TRINITY_DN50323_c0_g1) genes were co-expressed in vegetative propagation corms. Therefore, the upstream ATGs of these two genes might result in the low concentration of gastrodin in vegetative propagation corms. The induction of gene expression in response to fungi symbiosis might increase the translation efficiency or mRNA stability of the two key enzymes to increase gastrodin production in juvenile tubers. In response to stress, the translation repression of upstream ATGs/upstream ORFs could be significantly reduced [92–94] or mRNA stability could be increased [95, 96]. In response to fungi invasion, the repression reduction of the translation efficiency of both monooxygenases (unigene TRINITY_DN54282_c0_g1) and glycosyltransferase (unigene TRINITY_DN50323_c0_g1) genes in *G. elata* might also increase the accumulation of gastrodin in juvenile tubers, as fungal infection could be a biotic stress to *G. elata*.

## Conclusions

The molecular basis of gastrodin biosynthesis in *G. elata* was clarified based on *de novo* transcriptome sequencing in the present study. Two putative monooxygenase (unigene TRINITY_DN54282_c0_g1) and glycosyltransferase (unigene TRINITY_DN50323_c0_g1) genes associated with the gastrodin biosynthesis pathway were identified. The genes of the two key enzymes involved in gastrodin biosynthesis might be applied as the target genes for plant gene transformation in future studies to obtain transgenic plants or microbial hosts with gastrodin production. Moreover, this transcriptome dataset also provides important information to accelerate future gene expression and functional genomics studies in *G. elata*.

## Methods

### Materials

Plant materials used in this study were got from the Chinese medical farm. The voucher specimens were deposited at the herbarium of the Taiwan Endemic Species Research Institute (TAIE) and the voucher numbers are Hsu 17054 and 17055. The vegetative propagation corms and juvenile tubers of *G. elata* were, respectively, harvested 1 and 12 months after sowing at the Chinese medical farm (Hakusan City, Jilin Province, China) (Fig. 1b and d). *A. mellea* was isolated from the juvenile tuber of *G. elata* and identified based on ITS sequence of nuclear ribosomal DNA (nrDNA) (data not shown). The fungi were cultured on PDA medium according to Mishra and Dubey (1994) for further molecular studies (Fig. 1c) [97].

### RNA isolation, cDNA library preparation, deep sequencing and *de novo* assembly

The vegetative propagation corms of *G. elata*, juvenile tuber of *G. elata* and *A. mellea* was separately harvested and extracted total RNA using Trizol® Reagent (Invitrogen, Carlsbad, CA, USA) and followed by the RNeasy Plant Mini Kit (Qiagen, Hilden, Germany). Purified RNA was quantified at OD260 using a ND-1000 spectrophotometer (NanoDrop Technology, San Diego, CA, USA) and qualitated using an Agilent Bioanalyzer 2100 with the RNA 6000 nano labchip kit (Agilent Technology, Santa Clara, CA, USA). The RNA Integrity Number (RIN) was identified 8.7, 9.1, and 8.0 for vegetative propagation corm, juvenile tuber, and *A. mellea*, respectively. Each of the RNA libraries was separately constructed using TruSeq RNA Library Preparation Kit v2 (Illumina Inc., San Diego, CA) with standard Illumina protocols. After quality-control using an Agilent Bioanalyzer 2100, each library was paired-end deep sequenced by Illumina HiSeq 2000 sequencer (Illumina Inc., San Diego, CA, USA) according to the manufacturer’s instructions. The quality of the produced fastq sequences was assessed using FastQC program (http://www.bioinformatics.babraham.ac.uk/projects/fastqc/), see Additional files 12, 13 and 14: Figures S4, S5 and S6. Before the assembling stage, the reads were processed to trim adaptor sequences and low-quality ends via Trimmomatic version 0.32 [98] with parameters “ILLUMINACLIP: TruSeq3-PE.fa:2:30:10 SLIDINGWINDOW:4:5 LEADING:5 TRAILING:5 MINLEN:25”. A single *de novo* transcriptome assembly was generated from high-quality short reads of all three samples using the Trinity software (https://github.com/trinityrnaseq/trinityrnaseq/wiki/) [99]. Trinity v2.1.0 (release 20140413p1) was employed with the default *k-mer* of 25 and minimum assembled contig length of 200. Candidate coding regions within transcript sequences were identified using TransDecoder (http://transdecoder.github.io/).

### Transcript abundance estimation

Quantification of transcripts was estimated using the RNA-Seq by Expectation-Maximization (RSEM) software version 1.2.23 [100], which was bundled with the Trinity software distribution. The RSEM protocal uses the Bowtie software (http://bowtie-bio.sourceforge.net/index.shtml) [101] to align trimmed reads from each sample seperatively to the assembled transcripts, and then computes transcript abundance, estimates the number of aligned fragments corresponding to each transcript, including normalized expression values as FPKM for paried-end reads. In addition, RSEM computes ‘gene-level’ expression values using the Trinity component as a proxy for the gene. For comparing expression levels of different transcripts or genes across samples, a Trinity-bundled script invokes the EdgeR package to perform an additional TMM scaling normalization that aims to estimate differences in total RNA production across all samples [102, 103]. Transcripts with zero TMM-normalized FPKM values for all three samples were removed from the assembly and not counted into the total transcript number, and the rest with longest length per component (i.e., gene locus) defined by Trinity were interpreted to represent “unigenes” for downstream analysis. The data presented in this publication have been deposited in NCBI’s Gene Expression Omnibus [104] and are accessible through GEO Series accession number GSE73633 (https://www.ncbi.nlm.nih.gov/geo/query/acc.cgi?acc=GSE73633).

### Gene functional annotation, GO classification and KEGG pathway analysis

The functional annotation of the unigenes was conducted using the BLASTX algorithm of DIAMOND program [105] with E-values < 1.00E-5 and enabled ‘sensitive mode’ against NCBI non-redundant protein sequence database (nr, October 2015), which consisted of all non-redundant peptide sequences from GenBank CDS translations, RefSeq Proteins, PDB (Protein Data Bank) database, SwissProt database, PIR (Protein Information Resource) database, and PRF (Protein Research Foundation) database. Gene Ontology (GO) annotations of unigenes was performed by coverting the GeneBank identifiers (gi) of hits from the BLASTX results into UniProt IDs through the IDmapping data files [106] downloaded from The Universal Protein Resource (UniProt; http://www.uniprot.org/), then the corresponding GO terms were retrieved via the UniProt IDs using home-made Perl scripts. KEGG (http://www.kegg.jp/) [107] pathway annotation of unigenes was performed using the BLASTX algorithm of DIAMOND program with E-values < 1.00E-5 and enabled ‘sensitive mode’ against KEGG gene peptide database (October, 2015), and then the corresponding KO identifiers, EC numbers and pathway categories were parsed using home-made Perl scripts.

### Comparative analysis of differentially expressed genes

The number of unigenes per sample was counted if the corresponding TMM-normalized FPKM values of unigenes above zero. For differential expression analysis, each pairwise comparison was performed from the TMM-normalized FPKM values using the R package limma (Linear Models for Microarray and RNA-Seq Data) [108] with upper-quantile normalization. Zero TMM-normalized FPKM values were replaced to 0.001 from either sample to avoid problems associated with zero value. A number of statistics were calculated, including log2-FC (fold changes) between the two samples, the *p*-values, and associated *q*-values (FDR-corrected *p*-values). Differentially expressed unigenes were selected using the criteria that the absolute value of the log2-FC ≥ 1, the *q*-values < 0.05, and different TMM-normalized FPKM values under variant stringency. Genes with a *q*-value < 0.05 were considered to be differentially expressed [109].

### Semi-quantitative RT-PCR, real-time RT-PCR analysis and SMART-RACE cDNA amplification

The cDNA products were diluted 20-fold with deionized water prior to use as a template in semi-quantitative RT-PCR and real-time PCR [110]. Semi-quantitative RT-PCR reactions were performed in 20 μl reactions containing gene-specific primers [See Additional file 15: Table S9] and the ubiquitin gene primer as an internal control [108, 111]. Additional reaction components were 1X Red Taq Mastermix (RBC Bioscience, Taipei, Taiwan), 1 mM MgCl_2_ and 1 μM of the specific primers. Following PCR amplification, 5 μl of the PCR products were separated on a 1 % TAE agarose gel containing ethidium bromide, and the bands were photographed under UV light using gel documentation system alpha imager EC (Alpha Innotech, Japan). Real-time RT-PCR was performed using the Power SYBR Green PCR Master Mix (Applied Biosystems, Foster City, CA, USA) and a 7900HT Fast Real-Time PCR System (Applied Biosystems, Foster City, CA, USA) according to the manufacturer’s instructions. SDS2.2.2 software (Applied Biosystems, Foster City, CA, USA) was used for the comparative ΔC_t_ analysis, and the ubiquitin gene served as an internal control. The relative gene expression was calculated using the 2^-ΔΔCt^ method [112]. In SMART-RACE cDNA amplification, the 5′ and 3′-RACE (5′ and 3′-rapid amplification of cDNA ends) was performed using the SMART-RACE cDNA amplification kit (Clontech, Palo Alto, CA, USA) according to the manufacturer’s instructions. All primers used in the present study are listed in Additional file 15: Table S9.

## Availability of supporting data

The data set supporting the results of this article are available in the NCBI GEO repository, with the accession numbers GSE73633 (https://www.ncbi.nlm.nih.gov/geo/query/acc.cgi?acc=GSE73633). All the supporting data are included as additional files.

## Additional files

Additional file 1: Figure S1. Gene ontology (GO) term classifications under the molecular function, biological process and cellular compartment categories at level 2 derived from the mapped unigenes using read data from (a) *Armillariella mellea*, (b) vegetative propagation corm of *Gastrodia elata*, and (c) juvenile tuber of *Gastrodia elata*. (PDF 227 kb) Additional file 2: Figure S2. Unigenes identified solely or both for *Armillaria mellea* and juvenile tuber of *Gastrodia elata* in the comparative transcriptome analysis. (PDF 185 kb) Additional file 3: Table S1. Sixty-nine unigenes were significantly up-regulated (log2-FC ≥1, *q*-value < 0.05, TMM-normalized FPKM > 0.3) in *Armillaria mellea* compared with juvenile tuber of *Gastrodia elata. (PDF 170 kb)*
Additional file 4: Table S2. 223 unigenes were significantly down-regulated (log2-FC ≤ -1, *q*-value < 0.05, TMM-normalized FPKM > 0.3) in *Armillaria mellea* compared with juvenile tuber of *Gastrodia elata*. (PDF 176 kb) Additional file 5: Table S3. Mapping of KEGG biological pathways for up-regulated (log2-FC ≥ 1, *q*-value < 0. 05, TMM-normalized FPKM > 0.3) unigenes from *Armillaria mellea* compared to juvenile tuber of *G. elata. (PDF 125 kb)*
Additional file 6: Table S4. Mapping of KEGG biological pathways for down-regulated (log2-FC ≤ -1, *q*-value < 0.05, TMM-normalized FPKM > 0.3) unigenes from *Armillaria mellea* compared to juvenile tuber of *G. elata. (PDF 130 kb)*
Additional file 7: Figure S3. Unigenes identified solely or both for vegetative propagation corm and juvenile tuber of *Gastrodia elata* in the comparative transcriptome analysis. (PDF 202 kb) Additional file 8: Table S5. 298 unigenes were significantly up-regulated (log2-FC ≥ 1, *q*-value < 0.05, TMM-normalized FPKM > 10) in juvenile tubers compared with vegetative propagation corms of *Gastrodia elata*. (PDF 183 kb) Additional file 9: Table S6. 405 unigenes were significantly down-regulated (log2-FC ≤ -1, *q*-value < 0.05, TMM-normalized FPKM > 10) in juvenile tubers compared with vegetative propagation corms of *Gastrodia elata*. (PDF 168 kb) Additional file 10: Table S7. Mapping of KEGG biological pathways for up-regulated (log2-FC ≥ 1, *q*-value < 0.05, TMM-normalized FPKM > 10) unigenes from juvenile tuber of *G. elata* compared to vegetative propagation corm of *G. elata*.. (PDF 131 kb) Additional file 11: Table S8. Mapping of KEGG biological pathways for down-regulated ((log2-FC ≤ -1, *q*-value < 0.05, TMM-normalized FPKM > 10) unigenes from juvenile tuber of *G. elata* compared to vegetative propagation corm of *G. elata*. (PDF 130 kb) Additional file 12: Figure S4.
*Armillaria mellea* fastq sequences checked by FastQC for (a) per base sequence quality and (b) per sequence quality scores. (a): The y-axis on the graph shows the quality scores. The higher the score the better the base call. The background of the graph divides the y axis into very good quality calls (green), calls of reasonable quality (orange), and calls of poor quality (red). The central red line is the median value. The yellow box represents the inter-quartile range (25-75 %). The upper and lower whiskers represent the 10 and 90 % points. The blue line represents the mean quality. (b): The most frequently observed mean quality below 38 is meaning that this equates to a 0.0158 % error rate. (PDF 108 kb) Additional file 13: Figure S5. Vegetative propagation corm of *Gastrodia elata* fastq sequences checked by FastQC for (a) per base sequence quality and (b) per sequence quality scores. (a): The y-axis on the graph shows the quality scores. The higher the score the better the base call. The background of the graph divides the y axis into very good quality calls (green), calls of reasonable quality (orange), and calls of poor quality (red). The central red line is the median value. The yellow box represents the inter-quartile range (25–75 %). The upper and lower whiskers represent the 10 and 90 % points. The blue line represents the mean quality. (b): The most frequently observed mean quality below 39 is meaning that this equates to a 0.0126 % error rate. (PDF 109 kb) Additional file 14: Figure S6. Juvenile tuber of *Gastrodia elata* fastq sequences checked by FastQC for (a) per base sequence quality and (b) per sequence quality scores. (a): The y-axis on the graph shows the quality scores. The higher the score the better the base call. The background of the graph divides the y axis into very good quality calls (green), calls of reasonable quality (orange), and calls of poor quality (red). The central red line is the median value. The yellow box represents the inter-quartile range (25–75 %). The upper and lower whiskers represent the 10 and 90 % points. The blue line represents the mean quality. (b): The most frequently observed mean quality below 39 is meaning that this equates to a 0.0126 % error rate. (PDF 109 kb) Additional file 15: Table S9. List of primers used for SMART-RACE and Real-time PCR analysis. (PDF 80 kb)

## Abbreviations

CYP450: cytochrome P450
EC: enzyme commission
EST: expressed sequence tag
FPKM: fragments per kilobase of transcript per Million mapped reads
GAFPs: gastrodia antifungal proteins
GO: Gene Ontology
KEGG: Kyoto Encyclopedia of Genes and Genomes
KOids: KEGG Ortholog identifiers
NGS: next generation sequencing
PDB: protein data bank
PIR: protein information resource
PRF: protein research foundation
RIN: RNA integrity number
RSEM: RNA-seq by expectation-maximization
TAIE: Taiwan Endemic Species Research Institute
TMM: trimmed mean of M-values
TSA: transcriptome shotgun assembly

## References

[1] Zhang W, Li B (1980). The biological relationship of *Gastrodia elata* and *Armillaria mellea*. Acta Bot Sin.

[2] Huang H, Liang Z, Wang W (2004). Review of nutrient source studies on *Gastrodia elata* Bl growth up. J Northwest Sci Tech Univ Agric For.

[3] Park E, Lee W, Ahn J (2012). In vitro propagation of myco-heterotrophic *Gastrodia elata*. Hortic Environ Biotech.

[4] Liu H, Luo Y, Liu H (2010). Studies of mycorrhizal fungi of Chinese orchids and their role in orchid conservation in China-A review. Bot Rev.

[5] Park E, Lee W (2013). In vitro symbiotic germination of myco-heterotrophic *Gastrodia elata* by *Mycena* species. Plant Biotech Rep.

[6] Xu J, Gao S (2000). Retrospect on the research of the cultivation of *Gastrodia elata* Bl, a rare traditional Chinese medicine. Chinese Med J.

[7] Xu J, Ran Y, Mou C, Wang C, Cao J, Wang M (1981). A brief report on the nutritious sources of seed germination of *Gastrodia elata*. Bull Chin Mat Med.

[8] Xu J, Ran Y, Guo S (1989). Studies on the life cycle of *Gastrodia elata*. Acta Acad Med Sin.

[9] Zhou J, Yang Y, Liang H, Liu C (1987). Morphology of *Gastrodia elata*.

[10] Liu T, Li CM, Han YL, Chiang TY, Chiang YC, Sung HM (2015). Highly diversified fungi are associated with the achlorophyllous orchid *Gastrodia flavilabella*. BMC Genomics.

[11] Fan L, Guo S (1999). Interaction between protocorms of *Gastrodia elata* (orchidaceae) and *Mycena dendrobii* in symbiotic germination. Mycosystema.

[12] Fan L, Guo S, Xiao P (2001). Interaction between protocorms of *Gastrodia elata* (orchidaceae) and *Mycena anoectochila* during symbiotic germination. Mycosystema.

[13] Fan L, Guo S, Cao W, Xiao P, Xu J (1996). Isolation, culture, identification and biological activity of *Mycena orchidicola* sp. nov. in*Cymbidium sinense* (orchidaceae). Acta Mycol Sin.

[14] Guo S, Fan L, Cao W, Xu J, Xiao P (1997). *Mycena anoectochila* sp. nov. isolated from mycorrhizal roots of *Anoectochilus roxburghii* from Xishuangbanna, China. Mycologia.

[15] Guo S, Fan L, Cao W, Chen X (1999). *Mycena dendrobii*, a new mycorrhizal fungus. Mycosystema.

[16] Xu J, Guo X (1989). Fungus associated with nutrition of seed germination of *Gastrodia elata-Mycena osmundicola* Lange. Acta Mycol Sin.

[17] Guo S, Xu J (1990). Studies on the cell ultrastructure in the course of *Gastrodia elata* digesting *Mycena osmundicola* LANGE and *Armillaria mellea FR*. Acta Mycol Sin.

[18] Kusano S (1911). *Gastrodia elata* and its symbiotic association with *Armillaria mellea*. J Coll Agric Imp Univ Tokyo.

[19] Zou N, Bai X, Lu J, Yang J, Xu G, Sun D (2010). Study on symbiotic mechanism between *Gastrodia elata* Blume and *Armillaria mellea* in tissue culture system. Med Plant.

[20] Lan J, Xu J, Li J (1986). Studies on the infecting process of labelled *Armillaria mellea* to *Gastrodia elata*. Acta Agric Nucleatae Sinica.

[21] Xu J (2001). The changes of cell structure in the courses of Armillaria mellea penetrating the nutritional stems of *Gastrodia elata*. Acta Acad Med Sin.

[22] Xu J, Fan L (2001). Cytodifferentiation of the seeds (protocorms) and vegetative propagation corms colonized by mycorrhizal fungi. Acta Bot Sin.

[23] Tang W, Eisenbrand G (1992). *Gastrodia elata* B1. Chinese drugs of plant origin.

[24] Bensky D, Gamble A (1993). Chinese herbal medicine: materia medica. Revised Edition.

[25] Jung T, Suh S, Lee H, Kim I, Kim H, Yoo H (2007). Protective effects of several components of *Gastrodia elata* on lipid peroxidation in gerbil brain homogenates. Phytother Res.

[26] Kim D, Kim J, Han Y (2007). Alzheimer’s disease drug discovery from herbs: neuroprotectivity from β-Amyloid (1–42) insult. J Altern Complement Med.

[27] Feng X, Chen Y, Yang J (1979). Studies on constituents of Tian-Ma.*Gastrodia elata* Bl. Acta Chim Sin.

[28] Zhou J, Yang Y, Yang J (1979). Chemistry of *Gastrodia elata* Bl. I. Isolation and identification of chemical constituents of *Gastrodia elata* Bl. Acta Chim Sin.

[29] Zhao Y, Cao Q, Xiang Y, Hu Z (1999). Identification and determination of active components in *Gastrodia elata* Bl by capillary electrophoresis. J Chromatogr.

[30] Zhen J (1961). Study on actions of tuber (tian-ma) and vanillin on counteracting epilepsy. Chin J Physiol.

[31] Huh K, Kim J, Kwon T (1998). The mechanism of anticonvulsive effect of the rhizoma of *Gastrodia elata* in pentylenetetrazole treated rats. Yakhak Hoechi.

[32] Hsieh C, Tang N, Chiang S, Hsieh C, Lin J (1999). Anticonvulsive and free radical scavenging actions of two herbs, *Uncaria rhynchophylla* (MIQ) Jack and *Gastrodia elata* Bl., in kainic acid-treated rats. Life Sci.

[33] Ha J, Lee D, Lee J, Kim J, Yong C, Kim J (2000). 4-Hydroxybenzaldehyde from *Gastrodia elata* B1. is active in the antioxidation and GABAergic neuromodulation of the rat brain. J Ethnopharmacol.

[34] Hsieh C, Chang C, Chiang S, Li T, Tang N, Pon C (2000). Anticonvulsive and free radical scavenging activities of vanillyl alcohol in ferric chlorideinduced epileptic seizures in Sprague-awley rats. Life Sci.

[35] Ha JH, Shin SM, Lee SK, Kim JS, Shin US, Huh K (2001). In vitro effects of hydroxybenzaldehydes from *Gastrodia elata* and their analogues on GABAergic neurotransmission, and a structure–activity correlation. Planta Med.

[36] Kim H, Moon K, Oh S, Kim S, Lee S (2001). Ether fraction of methanol extracts of *Gastrodia elata*, a traditional medicinal herb, protects against kainic acid-induced neuronal damage in the mouse hippocampus. Neurosci Lett.

[37] Taguchi H, Yosioka I, Yamasaki K, Kim I (1981). Studies on the constituents of *Gastrodia elata* Blume. Chem Pharm Bull.

[38] Huang NK, Chern Y, Fang JM, Lin CI, Chen WP, Lin YL (2007). Neuroprotective principles from *Gastrodia elata*. J Nat Prod.

[39] Yang X, Zhu J, Yang R, Liu J, Li L, Zhang H (2007). Phenolic constituents from the rhizomes of *Gastrodia elata*. Nat Prod Res.

[40] Gong X, Sucher N (1999). Stroke therapy in traditional medicine (TCM): prospects for drug discovery and development. TiPS.

[41] Jin W, Tian D (2000). Studies on chemisty and pharmacology of *Gastrodia elata*. Chin Tradit Drug Tech.

[42] Yang S, Lan J, Xu J (2000). Progress of study on *Gastrodia elata*. Chin Tradit Herb Drugs.

[43] Zhou J, Yang Y, Yang C (1980). Chemical study on gastrodin and related compounds, the synthese of gastrodin and related compounds. Acta Chem Sin.

[44] Ye H, Wang J, Wang S, Tan D (2003). Compare seed stem with commercial rhizoma *Gastrodia* in pharmacologic effect II. Lishizhen Med Mat Med Res.

[45] Zhu H, Dai P, Zhang W, Chen E, Han W, Chen C (2010). Enzymic synthesis of gastrodin through microbial transformation and purification of gastrodin biosynthesis enzyme. Biol Pharm Bull.

[46] Peng CX, Gong JS, Zhang XF, Zhang M, Zheng SQ (2008). Production of gastrodin through biotransformation of *p*-hydroxybenzyl alcohol using hairy root cultures of *Datura tatula* L. Afr J Biotechnol.

[47] Zhang H, He G, Liu J, Ruan H, Chen Q, Zhang Q (2008). Production of gastrodin through biotransformation of p-2-hydroxybenzyl alcohol by cultured cells of *Armillaria luteovirens* Sacc. Enzyme Microb Technol.

[48] Sun C, Li Y, Wu Q, Luo H, Sun Y, Song J (2010). *De novo* sequencing and analysis of the American ginseng root transcriptome using a GS FLX Titanium platform to discover putative genes involved in ginsenoside biosynthesis. BMC Genomics.

[49] Kouko J, Conn E (1961). The metabolism of aromatic compounds in higher plants: IV. Purification and propertiles of the phenylalanine deaminase of *Hordeum vulgare*. J Biol Chem.

[50] Heiden A, Kobel K, Komenda M, Koppmann R, Shao M, Wildt J (1999). Toluene emissions from plants. Geophys Res Lett.

[51] Singer A, Crowley D, Thompson I (2003). Secondary plant metabolites in phytoremediation and biotransformation. TRENDS Biotech.

[52] Rasi S, Veijanen A, Rintala J (2007). Trace compounds of biogas from different biogas production plants. Energy.

[53] Zhang H. Research on production of gastrodin through biotransformation by Armillaria luteo-virens Sacc. Master’s thesis, Zhejiang University. 2010.

[54] Carmona M, Zamarro M, Blazquez B, Durante-Rodriguez G, Juarez J, Valderrama J (2009). Anaerobic catabolism of aromatic compounds: a genetic and genomic view. Microbiol Mol Biol Rev.

[55] Radniecki T, Gilroy C, Semprini L (2011). Linking NE1545 gene expression with cell volume changes in *Nitrosomonas europaea* cells exposed to aromatic hydrocarbons. Chemosphere.

[56] Jones K, Trudgill P, Hopper D (1993). Metabolism of p-Cresol by the Fungus Aspergillus fumigatus. Microbiology.

[57] Coon M (2005). Cytochrome P450: nature’s most versatile biological catalyst. Annu Rev Pharmacol Toxicol.

[58] Morant M, Bak S, Moller B, Werck-Reichhart D (2003). Plant cytochromes P450: tools for pharmacology, plant protection and phytoremediation. Curr Opin Biotechnol.

[59] Metzker M (2010). Sequencing technologies-the next generation. Nat Rev Genet.

[60] Wakaguri H, Suzuki Y, Katayama T, Kawashima S, Kibukawa E, Hiranuka K (2009). Full-Malaria/Parasites and Full-Arthropods: databases of full-length cDNAs of parasites and arthropods. Nucleic Acids Res.

[61] Hua W, Zhang Y, Song J, Zhao L, Wang Z (2011). *De novo* transcriptome sequencing in *Salvia miltiorrhiza* to identify genes involved in the biosynthesis of active ingredients. Genomics.

[62] Wang W, Wang Y, Zhang Q, Qi Y, Guo D (2009). Global characterization of *Artemisia annua* glandular trichome transcriptome using 454 pyrosequencing. BMC Genomics.

[63] Barrero R, Chapman B, Yang Y, Moolhuijzen P, Keeble-Gagnère G, Zhang N (2011). *De novo* assembly of Euphorbia fischeriana root transcriptome identifies prostratin pathway related genes. BMC Genomics.

[64] Hao D, Ge G, Xiao P, Zhang Y, Yang L (2011). The first insight into the tissue specific taxus transcriptome via Illumina second generation sequencing. PLoS ONE.

[65] Liu S, Li W, Wu Y, Chen C, Lei J (2013). *De novo* transcriptome assembly in chili pepper (*Capsicum frutescens*) to identify genes involved in the biosynthesis of capsaicinoids. PLoS ONE.

[66] Vega-Arreguin J, Ibarra-Laclette E, Jimenez-Moraila B, Martinez O, Vielle-Calzada J, Herrera-Estrella L (2009). Deep sampling of the Palomero maize transcriptome by a high throughput strategy of pyrosequencing. BMC Genomics.

[67] Annadurai R, Neethiraj R, Jayakumar V, Damodaran A, Rao S, Katta M (2013). *De novo* transcriptome assembly (NGS) of *Curcuma longa* L. rhizome reveals novel transcripts related to anticancer and antimalarial terpenoids. PLoS ONE.

[68] Barakat A, DiLoreto D, Zhang Y, Smith C, Baier K, Powell W (2009). Comparison of the transcriptomes of American chestnut (*Castanea dentata*) and Chinese chestnut (*Castanea mollissima*) in response to the chestnut blight infection. BMC Plant Biol.

[69] Mizrachi E, Hefer C, Ranik M, Joubert F, Myburg A (2010). *De novo* assembled expressed gene catalog of a fast-growing *Eucalyptus* tree produced by Illumina mRNA-Seq. BMC Genomics.

[70] Alagna F, D’Agostino N, Torchia L, Servili M, Rao R, Pietrella M (2009). Comparative 454 pyrosequencing of transcripts from two olive genotypes during fruit development. BMC Genomics.

[71] Shi C, Yang H, Wei C, Yu O, Zhang Z, Jiang C (2011). Deep sequencing of the *Camellia sinensis* transcriptome revealed candidate genes for major metabolic pathways of tea-specific compounds. BMC Genomics.

[72] Wang Z, Fang B, Chen J, Zhang X, Luo Z, Huang L (2010). *De novo* assembly and characterization of root transcriptome using Illumina paired-end sequencing and development of cSSR markers in sweet potato (*Ipomoea batatas*). BMC Genomics.

[73] Weber A, Weber K, Carr K, Wilkerson C, Ohlrogge J (2007). Sampling the Arabidopsis transcriptome with massively parallel pyrosequencing. Plant Physiol.

[74] Su C, Chao Y, Chang Y, Chen W, Chen C, Lee A (2011). *De novo* assembly of expressed transcripts and global analysis of the *Phalaenopsis aphrodite* transcriptome. Plant Cell Physiol.

[75] Feng C, Chen M, Xu C, Bai L, Yin X, Li X (2012). Transcriptomic analysis ofChinese bayberry (*Myrica rubra*) fruit development and ripening using RNA-Seq. BMC Genomics.

[76] Kudapa H, Bharti A, Cannon S, Farmer A, Mulaosmanovic B, Kramer R (2012). A comprehensive transcriptome assembly of Pigeonpea (*Cajanus cajan* L.) using sanger and second-generation sequencing platforms. Mol Plant.

[77] Ranjan A, Ichihashi Y, Farhi M, Zumstein K, Townsley B, David-Schwartz R (2014). *De novo* assembly and characterization of the transcriptome of the parasitic weed *Cuscuta pentagona* identifies genes associated with plant parasitism. Plant Physiol.

[78] Salgado L, Koop D, Pinheiro D, Rivallan R, Guen V, Nicolás M (2014). *De novo* transcriptome analysis of *Hevea brasiliensis* tissues by RNA-seq and screening for molecular markers. BMC Genomics.

[79] Wang Y, Pan Y, Liu Z, Zhu X, Zhai L, Xu L (2013). *De novo* transcriptome sequencing of radish (*Raphanus sativus* L.) and analysis of major genes involved in glucosinolate metabolism. BMC Genomics.

[80] Trapnell C, Williams BA, Pertea G, Mortazavi A, Kwan G, van Baren MJ (2010). Transcript assembly and quantification by RNA-Seq reveals unannotated transcripts and isoform switching during cell differentiation. Nat Biotechnol.

[81] Wang HX, Yang T, Zeng Y, Hu Z (2007). Identification of the gastrodianin gene ga4B in an achlorophyllous plant Gastrodia elata Orchidaceae and its expression pattern during life cycle. Acta Bot Yunnanica.

[82] Hu Z, Yang Z, Wang J (1988). Isolation and partial characterization of an antifungal protein from *Gastrodia elata* corm. Acta Bot Yunnan.

[83] Shiau Y, Ho W (2012). Method for extraction of gastrodin in *Anoectochilus formosanus* Hayata. J Taiwan Agric Res.

[84] Bowles D, Lim E, Poppenberger B, Vaistij F (2006). Glycosyltransferases of lipophilic small molecules. Annu Rev Plant Biol.

[85] Gachon C, Langlois-Meurinne M, Saindrenan P (2005). Plant secondary metabolism glycosyltransferases: the emerging functional analysis. Trends Plant Sci.

[86] Lairson L, Henrissat B, Davies G, Withers S (2008). Glycosyltransferases: structures, functions, and mechanisms. Annu Rev Biochem.

[87] Kozak M (1991). Structural features in eukaryotic mRNAs that modulate the initiation of translation. J Biol Chem.

[88] Morris DR, Geballe AP (2000). Upstream open reading frames as regulators of mRNA translation. Mol Cell Biol.

[89] Mendell JT, Sharifi NA, Meyers JL, Martinez-Murillo F, Dietz HC (2004). Nonsense surveillance regulates expression of diverse classes of mammalian transcripts and mutes genomic noise. Nat Genet.

[90] Wittmann J, Hol EM, Jack H-M (2006). hUPF2 silencing identifies physiologic substrates of mammalian nonsense-mediated mRNA decay. Mol Cell Biol.

[91] Yepiskoposyan H, Aeschimann F, Nilsson D, Okoniewski M, Muhlemann O (2011). Autoregulation of the nonsense-mediated mRNA decay pathway in human cells. RNA.

[92] Watatani Y, Ichikawa K, Nakanishi N, Fujimoto M, Takeda H, Kimura N (2008). Is regulated by the 5′-untranslated region stress-induced translation of ATF5 mRNA. J Biol Chem.

[93] Spriggs KA, Bushell M, Willis AE (2010). Translational regulation of gene expression during conditions of cell stress. Mol Cell.

[94] Barbosa C, Romao L (2014). Translation of the human erythropoietin transcript is regulated by an upstream open reading frame in response to hypoxia. RNA.

[95] Zhao C, Datta S, Mandal P, Xu S, Hamilton T (2010). Stress-sensitive regulation of IFRD1 mRNA decay is mediated by an upstream open reading frame. J Biol Chem.

[96] Hatano M, Umemura M, Kimura N, Yamazaki T, Takeda H, Nakano H (2013). The 5′-untranslated region regulates ATF5 mRNA stability via nonsense-mediated mRNA decay in response to environmental stress. FEBS J.

[97] Mishra A, Dubey N (1994). Evaluation of some essential oils for their toxicity against fungi causing deterioration of stored food commodities. Appl Environ Microbiol.

[98] Bolger AM, Lohse M, Usadel B (2014). Trimmomatic: a flexible trimmer for Illumina sequence data. Bioinformatics.

[99] Grabherr MG, Haas BJ, Yassour M, Levin JZ, Thompson DA, Amit I (2011). Full-length transcriptome assembly from RNA-Seq data without a reference genome. Nat Biotechnol.

[100] Li B, Dewey CN (2011). RSEM: accurate transcript quantification from RNA-Seq data with or without a reference genome. BMC Bioinformatics.

[101] Langmead B, Trapnell C, Pop M, Salzberg S (2009). Ultrafast and memoryefficient alignment of short DNA sequences to the human genome. Genome Biol.

[102] Robinson MD, Oshlack A (2010). A scaling normalization method for differential expression analysis of RNA-seq data. Genome Biol.

[103] Dillies MA, Rau A, Aubert J, Hennequet-Antier C, Jeanmougin M, Servant N (2013). A comprehensive evaluation of normalization methods for Illumina high-throughput RNA sequencing data analysis. Brief Bioinform.

[104] Edgar R, Domrachev M, Lash AE (2002). Gene expression omnibus: NCBI gene expression and hybridization array data repository. Nucleic Acids Res.

[105] Buchfink B, Xie C, Huson DH (2015). Fast and sensitive protein alignment using DIAMOND. Nat Methods.

[106] Huang H, McGarvey PB, Suzek BE, Mazumder R, Zhang J, Chen Y (2011). A comprehensive protein-centric ID mapping service for molecular data integration. Bioinformatics.

[107] Kanehisa M, Goto S (2007). KEGG: Kyoto encyclopedia of genes and genomes. Nucleic Acids Res.

[108] Ritchie ME, Phipson B, Wu D, Hu Y, Law CW, Shi W (2015). Limma powers differential expression analyses for RNA-sequencing and microarray studies. Nucleic Acids Res.

[109] Benjamini Y, Hochberg Y (1995). Controlling the false discovery rate: a practical and powerful approach to multiple testing. J Royal Stat Soc B Methodol.

[110] García-Vallejo J, Van het Hof B, Robben J, Van Wijkb J, Van Diea I, Joziasse D (2004). Approach for defining endogenous reference genes in gene expression experiments. Anal Biochem.

[111] Pfaffl M, Tichopad A, Prgomet C, Neuvians T (2004). Determination of stable housekeeping genes, differentially regulated target genes and sample integrity: BestKeeper – Excel-based tool using pair-wise correlations. Biotechnol Lett.

[112] Livak K, Schmittgen T (2001). Analysis of relative gene expression data using real-time quantitative PCR and the 2^-ΔΔCT^ method. Methods.

