# Supplementary material for: Comparative transcriptome analysis of Gastrodia elata (Orchidaceae) in response to fungus symbiosis to identify gastrodin biosynthesis-related genes
Source: BMC Genomics. 2016 Mar 9;17:212. doi: 10.1186/s12864-016-2508-6 (PMC4784368; doi:10.1186/s12864-016-2508-6)
Supplement: Additional file 1: Figure S1. — Gene ontology (GO) term classifications under the molecular function, biological process and cellular compartment categories at level 2 derived from the mapped unigenes using read data from (a) Armillariella mellea, (b) vegetative propagation corm of Gastrodia elata, and (c) juvenile tuber of Gastrodia elata. (PDF 227 kb) [file 12864_2016_2508_MOESM1_ESM.pdf]

(a)

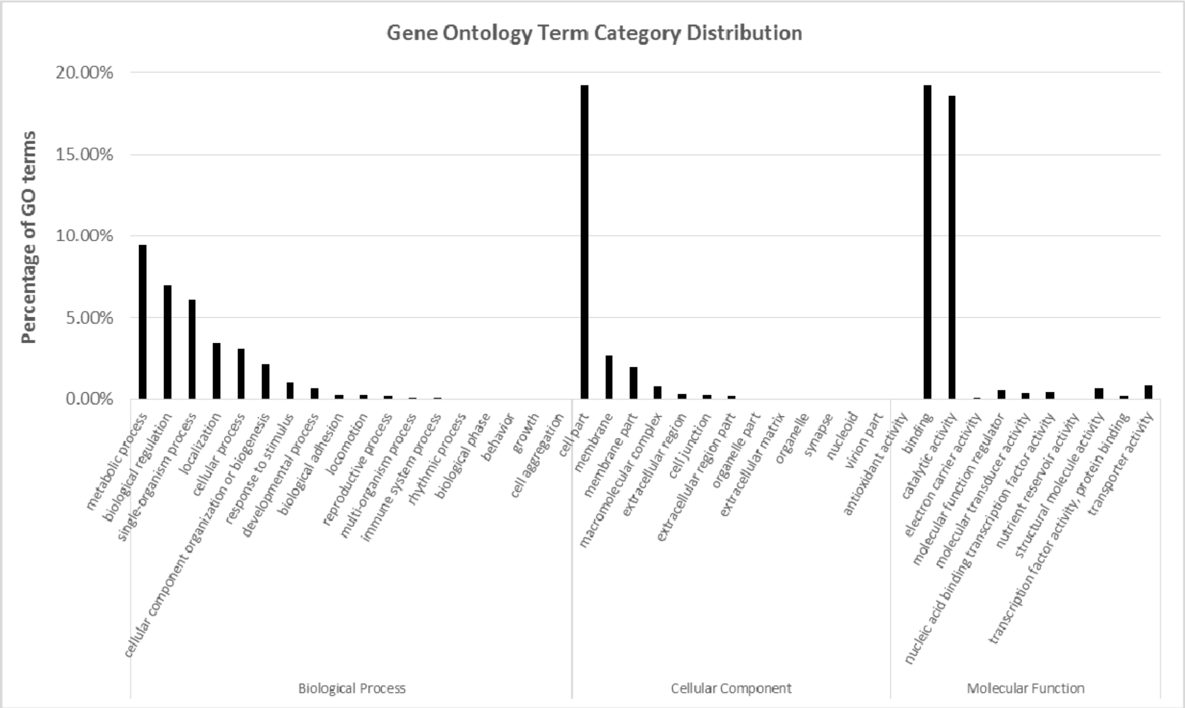
Gene Ontology Term Category Distribution
Percentage of GO terms
20.00%
15.00%
10.00%
5.00%
0.00%
metabolic process
biological regulation
single-organism process
localization
cellular process
cellular component organization or biogenesis
response to stimulus
developmental process
biological adhesion
locomotion
reproductive process
multi-organism process
immune system process
rhythmic process
biological phase
behavior
growth
cell aggregation
cell part
membrane
membrane part
macromolecular complex
extracellular region
cell junction
extracellular region part
organelle part
extracellular matrix
organelle
synapse
nucleoid
virion part
antioxidant activity
binding
catalytic activity
electron carrier activity
molecular function regulator
molecular transducer activity
nucleic acid binding transcription factor activity
nutrient reservoir activity
structural molecule activity
transcription factor activity, protein binding
transporter activity
Biological Process
Cellular Component
Molecular Function

(b)

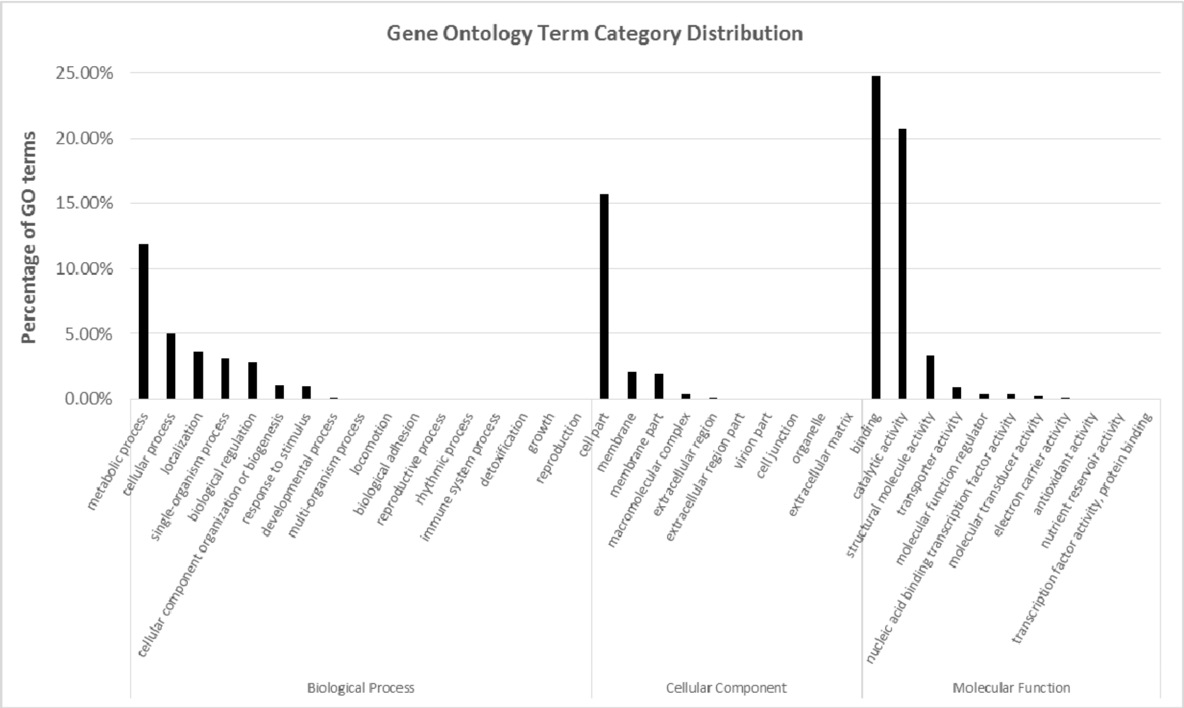
Gene Ontology Term Category Distribution
Percentage of GO terms
25.00%
20.00%
15.00%
10.00%
5.00%
0.00%
metabolic process
cellular process
localization
single-organism process
biological regulation
cellular component organization or biogenesis
response to stimulus
developmental process
multi-organism process
locomotion
biological adhesion
reproductive process
rhythmic process
immune system process
detoxification
growth
reproduction
cell part
membrane
membrane part
macromolecular complex
extracellular region
extracellular region part
virion part
cell junction
organelle
extracellular matrix
binding
catalytic activity
structural molecule activity
transporter activity
molecular function regulator
nucleic acid binding transcription factor activity
molecular transducer activity
electron carrier activity
antioxidant activity
nutrient reservoir activity
transcription factor activity, protein binding
Biological Process
Cellular Component
Molecular Function

(c)

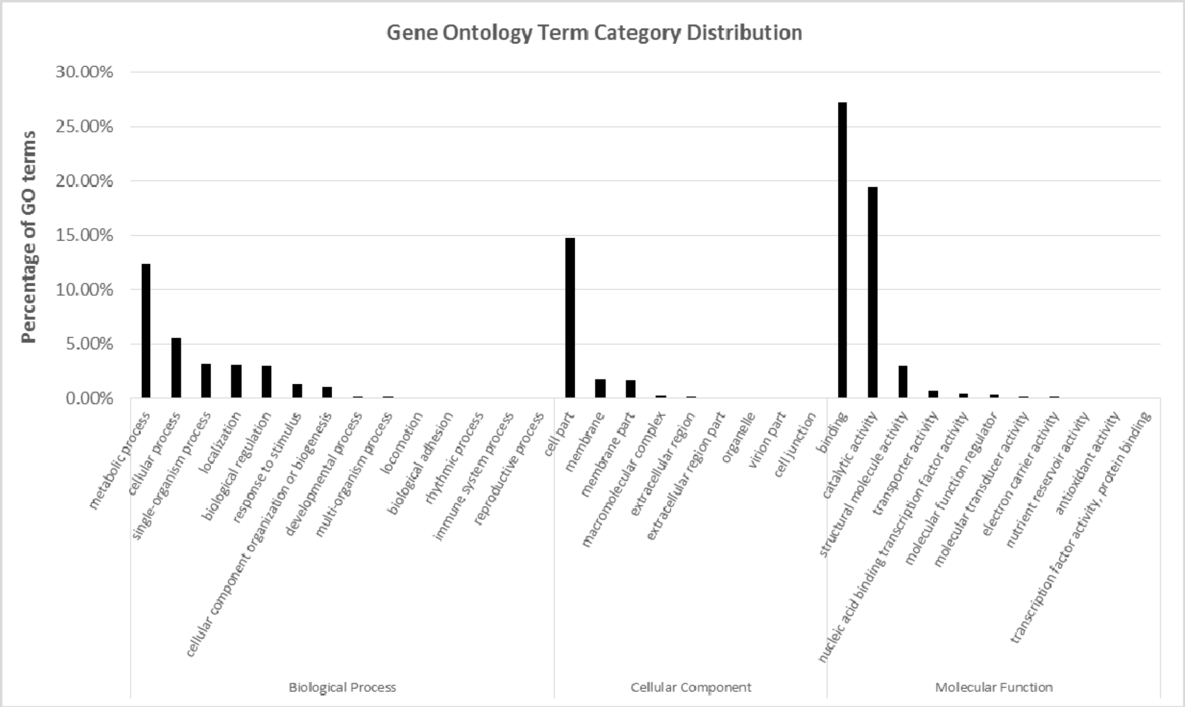

Additional file 1: Figure S1. Gene ontology (GO) term classifications under the molecular function, biological process and cellular compartment categories at level 2 derived from the mapped unigenes using read data from (a) *Armillariella mellea*, (b) vegetative propagation corm of *Gastrodia elata*, and (c) juvenile tuber of *Gastrodia elata*.
