# Supplementary material for: Comparative transcriptome analysis of Gastrodia elata (Orchidaceae) in response to fungus symbiosis to identify gastrodin biosynthesis-related genes
Source: BMC Genomics. 2016 Mar 9;17:212. doi: 10.1186/s12864-016-2508-6 (PMC4784368; doi:10.1186/s12864-016-2508-6)
Supplement: Additional file 2: Figure S2. — Unigenes identified solely or both for Armillaria mellea and juvenile tuber of Gastrodia elata in the comparative transcriptome analysis. (PDF 185 kb) [file 12864_2016_2508_MOESM2_ESM.pdf]

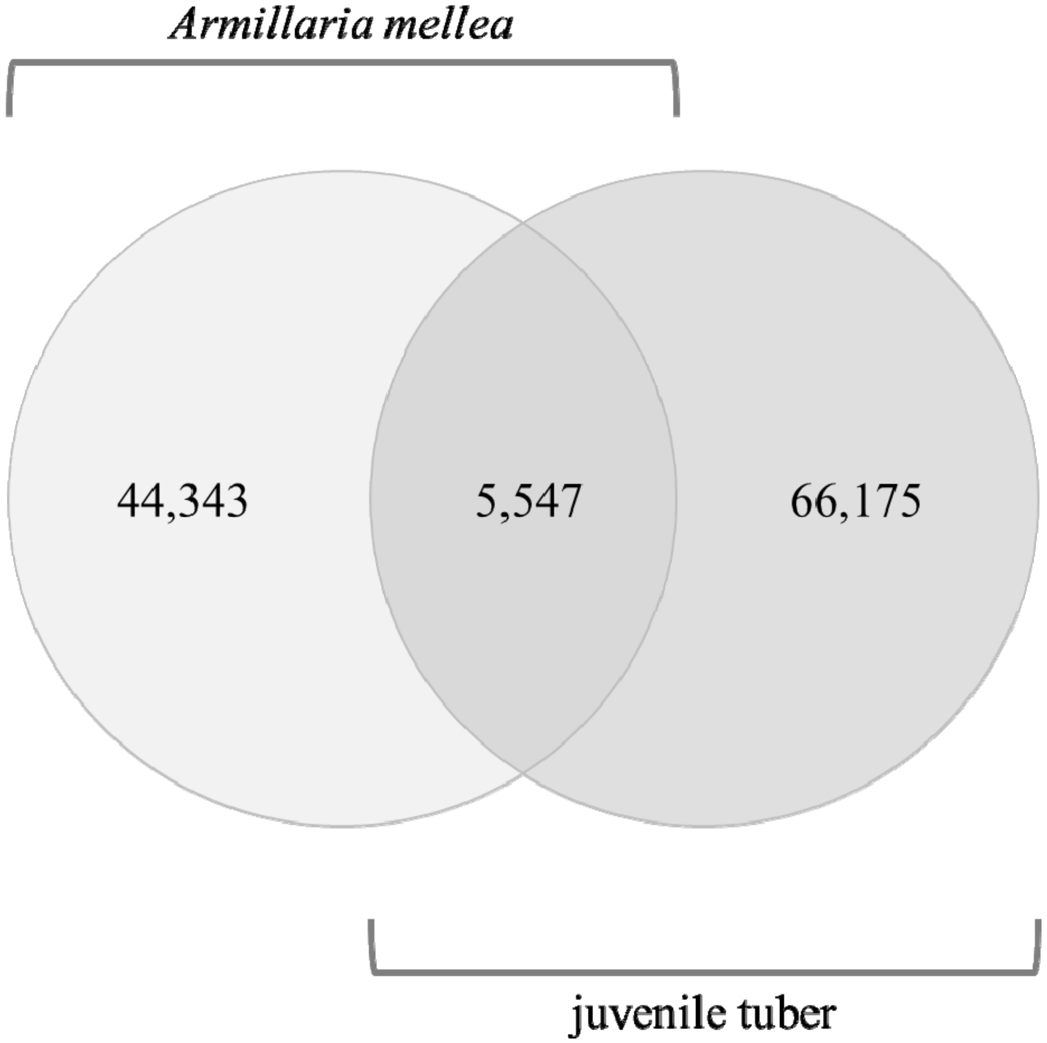

Additional file 2: Figure S2. Unigenes identified solely or both for *Armillaria mellea* and juvenile tuber of *Gastrodia elata* in the comparative transcriptome analysis.
