# Supplementary material for: Comparative transcriptome analysis of Gastrodia elata (Orchidaceae) in response to fungus symbiosis to identify gastrodin biosynthesis-related genes
Source: BMC Genomics. 2016 Mar 9;17:212. doi: 10.1186/s12864-016-2508-6 (PMC4784368; doi:10.1186/s12864-016-2508-6)
Supplement: Additional file 3: Table S1. — Sixty-nine unigenes were significantly up-regulated (log2-FC ≥1, q-value < 0.05, TMM-normalized FPKM > 0.3) in Armillaria mellea compared with juvenile tuber of Gastrodia elata. (PDF 170 kb) [file 12864_2016_2508_MOESM3_ESM.pdf]

Additional file 3: Table S1. Sixty-nine unigenes were significantly up-regulated (log2-FC ≥1, *q*-value < 0.05, TMM-normalized FPKM > 0.3) in *Armillaria mellea* compared with juvenile tuber of *Gastrodia elata*.

Unigene id	log2-FC	*q*-value	Hit accession
TRINITY_DN103263_c0_g1	1.758027	0.004641	ABK25179
TRINITY_DN103541_c0_g1	1.331409	0.006337	ABR16248
TRINITY_DN104027_c0_g1	1.762961	0.004597	XP_010713346
TRINITY_DN105719_c0_g1	2.117695	0.002471	ABK21163
TRINITY_DN14796_c0_g1	1.238787	0.006337	XP_012452367
TRINITY_DN15255_c0_g1	1.335603	0.006337	XP_422188
TRINITY_DN25069_c0_g1	2.409391	0.001592	-
TRINITY_DN26385_c0_g1	2.092757	0.002573	XP_010726358
TRINITY_DN2923_c0_g1	1.33963	0.006337	EMT29952
TRINITY_DN31655_c0_g1	1.304334	0.006337	XP_009334308
TRINITY_DN33079_c0_g1	2.131706	0.002416	ACD43781
TRINITY_DN33842_c0_g1	1.813587	0.004181	ABR18415
TRINITY_DN35410_c0_g1	10.83178	0.000256	XP_007315202
TRINITY_DN40716_c0_g1	4.988087	0.000295	-
TRINITY_DN41213_c1_g1	1.149747	0.007157	-
TRINITY_DN44709_c0_g1	13.9307	0.000256	-
TRINITY_DN46350_c0_g1	2.289507	0.001894	KFU91206
TRINITY_DN47304_c0_g1	1.023459	0.010228	XP_417505
TRINITY_DN48437_c0_g1	2.418583	0.001572	XP_002467304
TRINITY_DN48535_c0_g1	9.097447	0.000256	KIK48774
TRINITY_DN48650_c0_g1	2.81214	0.000956	CDX42303
TRINITY_DN48964_c0_g1	13.5188	0.000256	XP_001837318
TRINITY_DN50311_c0_g1	6.666172	0.000261	XP_418775
TRINITY_DN51255_c0_g1	11.93991	0.000256	XP_008040864
TRINITY_DN51898_c0_g2	1.53574	0.006337	XP_006017577
TRINITY_DN53449_c0_g1	11.02153	0.000256	KIK43154
TRINITY_DN54916_c0_g1	1.054884	0.009326	AJA33576
TRINITY_DN54922_c0_g2	13.92246	0.000256	KIY73555
TRINITY_DN55314_c0_g1	12.74911	0.000256	XP_007845736
TRINITY_DN5537_c0_g1	1.027203	0.010114	EPZ62736
TRINITY_DN55504_c2_g1	12.68599	0.000256	WP_015175893
TRINITY_DN55937_c0_g1	13.25514	0.000256	-
TRINITY_DN56395_c0_g1	12.91837	0.000256	-

TRINITY_DN57292_c0_g1	11.95982	0.000256	KDQ58530
TRINITY_DN57581_c1_g1	1.00738	0.010732	KOM48406
TRINITY_DN57749_c0_g1	10.07699	0.000256	-
TRINITY_DN59785_c0_g1	13.05511	0.000256	XP_001878516
TRINITY_DN60138_c0_g1	4.735509	0.000308	-
TRINITY_DN60777_c0_g1	13.96133	0.000256	KIY66619
TRINITY_DN60786_c0_g2	7.446493	0.000258	XP_007332183
TRINITY_DN61164_c14_g1	10.80964	0.000256	XP_001829580
TRINITY_DN61164_c4_g1	11.75414	0.000256	EGO00841
TRINITY_DN61389_c0_g2	7.335796	0.000258	KIK70050
TRINITY_DN62430_c0_g1	12.86687	0.000256	KIK68584
TRINITY_DN62705_c0_g1	3.119739	0.000697	XP_006685644
TRINITY_DN63467_c2_g3	3.584963	0.000484	KIK56569
TRINITY_DN64118_c0_g1	1.386058	0.006337	AAC49686
TRINITY_DN66240_c0_g1	1.366782	0.006337	-
TRINITY_DN70028_c0_g1	2.027631	0.002866	ABR16441
TRINITY_DN70364_c0_g1	1.33963	0.006337	-
TRINITY_DN72667_c0_g1	1.81214	0.004192	ACN40686
TRINITY_DN73087_c0_g1	1.386058	0.006337	ABK24012
TRINITY_DN76092_c0_g1	1.349334	0.006337	CBI36994
TRINITY_DN76314_c0_g1	1.771925	0.00452	-
TRINITY_DN76963_c0_g1	2.029231	0.002858	-
TRINITY_DN77974_c0_g1	1.798742	0.004298	AAC49686
TRINITY_DN78025_c0_g1	13.2544	0.000256	KIY65142
TRINITY_DN78522_c0_g1	1.117997	0.007801	AAA85367
TRINITY_DN78723_c0_g1	1.804984	0.004248	AJA33577
TRINITY_DN83238_c0_g1	1.327805	0.006337	NP_001291330
TRINITY_DN83644_c0_g1	1.354665	0.006337	XP_424857
TRINITY_DN83929_c0_g1	1.754888	0.004668	ABK24657
TRINITY_DN86189_c0_g1	1.363883	0.006337	ABK22623
TRINITY_DN90348_c0_g1	2.155278	0.002327	-
TRINITY_DN90469_c0_g1	2.547119	0.001321	-
TRINITY_DN90767_c0_g1	1.720958	0.004983	XP_010714729
TRINITY_DN93560_c0_g1	1.387926	0.006337	AIL29328
TRINITY_DN94357_c0_g1	2.613992	0.001212	XP_001234565
TRINITY_DN99558_c0_g1	12.49192	0.000256	KFA65880
