# Supplementary material for: Comparative transcriptome analysis of Gastrodia elata (Orchidaceae) in response to fungus symbiosis to identify gastrodin biosynthesis-related genes
Source: BMC Genomics. 2016 Mar 9;17:212. doi: 10.1186/s12864-016-2508-6 (PMC4784368; doi:10.1186/s12864-016-2508-6)
Supplement: Additional file 4: Table S2. — 223 unigenes were significantly down-regulated (log2-FC ≤ -1, q-value < 0.05, TMM-normalized FPKM > 0.3) in Armillaria mellea compared with juvenile tuber of Gastrodia elata. (PDF 176 kb) [file 12864_2016_2508_MOESM4_ESM.pdf]

Additional file 4: Table S2. 223 unigenes were significantly down-regulated (log2-FC ≤ -1, *q*-value < 0.05, TMM-normalized FPKM > 0.3) in *Armillaria mellea* compared with juvenile tuber of *Gastrodia elata*

Unigene id	log2-FC	*q*-value	Hit accession
TRINITY_DN100634_c0_g1	-10.4149	0.006337	XP_009408514
TRINITY_DN100900_c0_g1	-11.0193	0.006337	AGH14255
TRINITY_DN101949_c0_g1	-9.57495	0.006337	XP_009414180
TRINITY_DN103516_c0_g1	-1.75803	0.011301	-
TRINITY_DN104641_c0_g1	-1.25873	0.015353	-
TRINITY_DN12597_c0_g1	-12.4254	0.006337	XP_008775490
TRINITY_DN12597_c0_g2	-6.88738	0.006366	XP_008775490
TRINITY_DN14658_c0_g1	-11.2277	0.006337	XP_008801013
TRINITY_DN15433_c0_g1	-12.1383	0.006337	AIZ68155
TRINITY_DN15639_c0_g2	-8.28191	0.006337	XP_010918607
TRINITY_DN16081_c0_g1	-10.0218	0.006337	XP_009415269
TRINITY_DN20598_c0_g1	-1.2123	0.015935	-
TRINITY_DN20954_c0_g1	-1.78932	0.011136	-
TRINITY_DN2118_c0_g1	-11.8373	0.006337	XP_010514560
TRINITY_DN21223_c1_g1	-10.9986	0.006337	XP_011075552
TRINITY_DN21508_c0_g1	-10.2888	0.006337	XP_009402509
TRINITY_DN22596_c0_g1	-1.2448	0.015522	-
TRINITY_DN22891_c1_g2	-10.9876	0.006337	ACJ38541
TRINITY_DN22913_c1_g1	-1.27534	0.015158	-
TRINITY_DN23688_c1_g1	-1.78136	0.011178	-
TRINITY_DN24387_c0_g1	-11.1172	0.006337	XP_007043894
TRINITY_DN2511_c1_g1	-1.26187	0.015316	-
TRINITY_DN27631_c0_g1	-10.7145	0.006337	XP_009420546
TRINITY_DN27666_c0_g1	-9.51621	0.006337	XP_007137929
TRINITY_DN27869_c0_g1	-10.6776	0.006337	XP_011075889
TRINITY_DN27922_c1_g3	-9.50747	0.006337	XP_008793824
TRINITY_DN28094_c0_g1	-11.2991	0.006337	XP_010925425
TRINITY_DN28094_c0_g2	-8.22789	0.006337	XP_010925425
TRINITY_DN28522_c0_g1	-7.31902	0.006348	XP_010906464
TRINITY_DN29035_c0_g1	-11.1103	0.006337	XP_010551079
TRINITY_DN29256_c0_g1	-10.8717	0.006337	AAL16908AF420017_1
TRINITY_DN2929_c0_g1	-1.87447	0.010722	KGN53990
TRINITY_DN29534_c0_g2	-9.80678	0.006337	XP_008784360

TRINITY_DN30613_c0_g1	-11.3172	0.006337	XP_008784753
TRINITY_DN31233_c0_g1	-10.6103	0.006337	XP_009406308
TRINITY_DN33625_c1_g1	-7.30999	0.006348	XP_008780830
TRINITY_DN35306_c1_g1	-3.34042	0.007476	-
TRINITY_DN36282_c1_g1	-11.2472	0.006337	XP_008787350
TRINITY_DN36402_c0_g1	-1.21681	0.015876	XP_010531518
TRINITY_DN36631_c1_g1	-1.25987	0.01534	-
TRINITY_DN37078_c0_g3	-8.20569	0.006337	NP_001062064
TRINITY_DN37431_c0_g1	-1.80296	0.011067	-
TRINITY_DN38087_c0_g2	-11.1991	0.006337	AIK03054
TRINITY_DN38536_c0_g2	-10.2535	0.006337	AAT08648
TRINITY_DN38858_c0_g1	-12.8776	0.006337	XP_003548693
TRINITY_DN38858_c1_g2	-10.6473	0.006337	-
TRINITY_DN39104_c0_g1	-1.76553	0.011261	-
TRINITY_DN39639_c1_g1	-4.81112	0.00666	-
TRINITY_DN39959_c0_g1	-1.2854	0.015042	CAC84489
TRINITY_DN40308_c3_g1	-1.28999	0.01499	-
TRINITY_DN40472_c1_g1	-2.1836	0.009548	-
TRINITY_DN40679_c0_g1	-9.57413	0.006337	NP_001148325
TRINITY_DN40763_c0_g1	-10.1792	0.006337	XP_010920591
TRINITY_DN44220_c1_g1	-4.50873	0.006759	-
TRINITY_DN44440_c1_g1	-6.9685	0.006362	-
TRINITY_DN44615_c0_g1	-9.50099	0.006337	XP_009407247
TRINITY_DN44832_c0_g1	-10.9704	0.006337	XP_008776254
TRINITY_DN45841_c0_g2	-11.3542	0.006337	XP_010916678
TRINITY_DN46410_c0_g1	-1.24917	0.015468	-
TRINITY_DN46920_c0_g1	-9.94178	0.006337	XP_010912635
TRINITY_DN46986_c0_g4	-11.1196	0.006337	CBI28785
TRINITY_DN48522_c0_g1	-1.48278	0.013132	-
TRINITY_DN48867_c0_g1	-10.4238	0.006337	AAK59994
TRINITY_DN48996_c0_g3	-1.25873	0.015353	-
TRINITY_DN49004_c5_g1	-7.36899	0.006346	-
TRINITY_DN49061_c0_g1	-11.9509	0.006337	XP_006645179
TRINITY_DN49061_c2_g2	-10.6103	0.006337	-
TRINITY_DN49061_c3_g1	-12.1293	0.006337	XP_013717439
TRINITY_DN50006_c0_g2	-9.17329	0.006337	XP_010912299
TRINITY_DN50711_c5_g1	-6.66804	0.006378	XP_010906316
TRINITY_DN51276_c0_g1	-1.19794	0.016125	EYC17119

TRINITY_DN51372_c0_g1	-8.7902	0.006337	XP_008783554
TRINITY_DN51934_c2_g1	-6.58754	0.006383	-
TRINITY_DN52558_c1_g1	-3.93643	0.007026	-
TRINITY_DN52605_c0_g1	-10.5068	0.006337	-
TRINITY_DN53023_c0_g1	-11.4623	0.006337	KHN15441
TRINITY_DN53023_c0_g2	-11.7137	0.006337	XP_013599762
TRINITY_DN53183_c0_g1	-11.7929	0.006337	AAA20899
TRINITY_DN53745_c0_g2	-8.33486	0.006337	ERN16036
TRINITY_DN53745_c0_g3	-13.3133	0.006337	ERN16036
TRINITY_DN53745_c0_g5	-13.1445	0.006337	XP_013587907
TRINITY_DN53745_c1_g1	-11.1459	0.006337	-
TRINITY_DN53747_c1_g1	-5.36435	0.00653	XP_009411213
TRINITY_DN54082_c1_g1	-12.0559	0.006337	CAD22154
TRINITY_DN540_c0_g2	-10.5983	0.006337	XP_008812836
TRINITY_DN54448_c2_g1	-11.1324	0.006337	CDP13175
TRINITY_DN54785_c0_g1	-11.0321	0.006337	AGC23439
TRINITY_DN54952_c3_g1	-6.80805	0.00637	-
TRINITY_DN55215_c0_g1	-3.45545	0.00737	-
TRINITY_DN55283_c0_g1	-2.94371	0.007938	XP_009395879
TRINITY_DN55657_c2_g3	-10.1639	0.006337	NP_001064729
TRINITY_DN55841_c5_g1	-2.3388	0.009104	-
TRINITY_DN56486_c2_g1	-1.83825	0.010893	-
TRINITY_DN56805_c0_g1	-11.6161	0.006337	XP_008805761
TRINITY_DN56918_c1_g1	-8.56867	0.006337	XP_010907075
TRINITY_DN57246_c1_g1	-11.4525	0.006337	EKV14875
TRINITY_DN57440_c3_g1	-2.75416	0.00823	XP_009418693
TRINITY_DN57478_c2_g1	-3.44505	0.007379	XP_011099853
TRINITY_DN57663_c0_g1	-2.13386	0.009708	-
TRINITY_DN58270_c3_g1	-10.3834	0.006337	XP_010261221
TRINITY_DN58564_c0_g1	-3.74028	0.007149	XP_010909223
TRINITY_DN58783_c4_g2	-9.42257	0.006337	XP_010939730
TRINITY_DN59037_c1_g1	-4.91995	0.00663	-
TRINITY_DN59302_c0_g1	-8.87394	0.006337	XP_010939670
TRINITY_DN59361_c0_g1	-10.8446	0.006337	XP_008776897
TRINITY_DN59908_c1_g2	-6.90856	0.006365	-
TRINITY_DN60133_c0_g2	-4.383	0.006807	-
TRINITY_DN60183_c12_g1	-10.5363	0.006337	AGA17037
TRINITY_DN60307_c4_g1	-6.49267	0.006389	XP_008813450

TRINITY_DN60432_c3_g1	-5.82443	0.006457	-
TRINITY_DN60542_c2_g1	-11.2785	0.006337	AKG50115
TRINITY_DN60661_c2_g1	-4.19747	0.006889	BAG46932
TRINITY_DN60661_c4_g1	-5.33889	0.006535	CDY19671
TRINITY_DN60765_c6_g1	-11.2775	0.006337	XP_009384009
TRINITY_DN61074_c6_g1	-1.30908	0.014777	XP_007312009
TRINITY_DN61164_c13_g1	-9.52255	0.006337	-
TRINITY_DN61164_c2_g2	-8.34152	0.006337	XP_008809862
TRINITY_DN61164_c9_g1	-11.518	0.006337	XP_002267206
TRINITY_DN61204_c2_g1	-6.82453	0.006369	-
TRINITY_DN61204_c4_g2	-5.28737	0.006545	-
TRINITY_DN61257_c13_g1	-7.57336	0.006341	XP_008797502
TRINITY_DN61257_c8_g5	-9.98067	0.006337	XP_007020288
TRINITY_DN61353_c0_g2	-8.95606	0.006337	XP_010246318
TRINITY_DN61734_c12_g2	-7.92358	0.006337	-
TRINITY_DN61851_c4_g2	-9.62414	0.006337	-
TRINITY_DN61901_c0_g3	-9.36572	0.006337	XP_008777998
TRINITY_DN62266_c0_g1	-10.1603	0.006337	XP_010915281
TRINITY_DN62358_c6_g1	-8.52419	0.006337	XP_010919989
TRINITY_DN62610_c1_g2	-9.2663	0.006337	XP_009386029
TRINITY_DN62647_c2_g2	-2.03449	0.010058	XP_004350535
TRINITY_DN63038_c1_g3	-3.19715	0.007624	EOB10733
TRINITY_DN63038_c3_g1	-1.83735	0.010897	ADI18832
TRINITY_DN63213_c6_g1	-3.02975	0.007823	-
TRINITY_DN63449_c7_g2	-3.52098	0.007314	-
TRINITY_DN63466_c2_g1	-5.51239	0.006504	-
TRINITY_DN63477_c9_g3	-10.1	0.006337	XP_010908921
TRINITY_DN63564_c0_g1	-9.83708	0.006337	XP_002142680
TRINITY_DN63577_c0_g1	-10.2971	0.006337	XP_009394878
TRINITY_DN63689_c0_g1	-10.4117	0.006337	XP_002270182
TRINITY_DN63694_c0_g1	-10.3851	0.006337	CBI32005
TRINITY_DN63748_c0_g1	-9.25989	0.006337	XP_008786698
TRINITY_DN63780_c0_g1	-9.73168	0.006337	AKR76258
TRINITY_DN63819_c0_g1	-10.401	0.006337	ACQ41837
TRINITY_DN63849_c0_g1	-10.1365	0.006337	XP_006828325
TRINITY_DN64033_c0_g1	-10.3774	0.006337	XP_008798125
TRINITY_DN65010_c0_g1	-2.21864	0.009441	-
TRINITY_DN65110_c0_g1	-8.76305	0.006337	-

TRINITY_DN65487_c0_g1	-10.281	0.006337	XP_011100211
TRINITY_DN65534_c0_g1	-10.625	0.006337	XP_010912398
TRINITY_DN65885_c0_g1	-1.26303	0.015302	CSD41531
TRINITY_DN6678_c0_g1	-1.78069	0.011181	-
TRINITY_DN67434_c0_g1	-1.26573	0.01527	ABK22260
TRINITY_DN68064_c0_g1	-1.25873	0.015353	-
TRINITY_DN68480_c0_g1	-1.84088	0.01088	ABR18218
TRINITY_DN68643_c0_g1	-1.25678	0.015376	-
TRINITY_DN69289_c0_g1	-2.1964	0.009508	XP_012468306
TRINITY_DN70236_c0_g1	-1.79701	0.011097	-
TRINITY_DN7058_c0_g1	-1.25081	0.015449	CAN63660
TRINITY_DN70645_c0_g1	-10.9661	0.006337	XP_008787474
TRINITY_DN70658_c0_g1	-12.689	0.006337	XP_008370710
TRINITY_DN70666_c0_g1	-12.1832	0.006337	XP_004984672
TRINITY_DN70668_c0_g1	-12.1041	0.006337	AAG52664AF225410_1
TRINITY_DN70693_c0_g1	-12.3705	0.006337	XP_008793269
TRINITY_DN70694_c0_g1	-3.04307	0.007806	-
TRINITY_DN70694_c1_g1	-9.4958	0.006337	XP_010935461
TRINITY_DN70735_c0_g1	-11.0294	0.006337	XP_009404265
TRINITY_DN70806_c0_g1	-9.29399	0.006337	XP_008806788
TRINITY_DN70854_c0_g1	-9.84871	0.006337	XP_008812787
TRINITY_DN71276_c0_g1	-7.00721	0.006361	-
TRINITY_DN71300_c0_g1	-8.13311	0.006337	XP_010937279
TRINITY_DN71434_c0_g1	-9.84283	0.006337	XP_012469291
TRINITY_DN71441_c0_g1	-10.7145	0.006337	XP_008775117
TRINITY_DN71569_c0_g1	-3.06324	0.007781	-
TRINITY_DN71850_c0_g1	-10.3363	0.006337	CDO98927
TRINITY_DN72299_c0_g1	-9.38736	0.006337	EMS55792
TRINITY_DN72450_c0_g1	-1.84891	0.010842	AEW08684
TRINITY_DN72860_c0_g1	-1.27403	0.015173	-
TRINITY_DN7452_c0_g1	-2.18536	0.009543	XP_012472420
TRINITY_DN76044_c0_g1	-8.61731	0.006337	XP_010260125
TRINITY_DN76109_c0_g1	-2.20469	0.009483	-
TRINITY_DN76317_c0_g1	-1.82018	0.010981	XP_008350380
TRINITY_DN77003_c0_g1	-2.27775	0.00927	-
TRINITY_DN77413_c0_g1	-1.24189	0.015558	ABK24654
TRINITY_DN7758_c0_g1	-9.66313	0.006337	XP_010932283
TRINITY_DN77874_c0_g1	-10.8393	0.006337	XP_008791900

TRINITY_DN78012_c0_g1	-9.9372	0.006337	XP_009409585
TRINITY_DN78034_c0_g1	-10.812	0.006337	XP_009629758
TRINITY_DN78042_c0_g1	-11.2511	0.006337	XP_008795561
TRINITY_DN78147_c0_g1	-9.77853	0.006337	CDY70031
TRINITY_DN78260_c0_g1	-9.31284	0.006337	XP_010912255
TRINITY_DN78531_c0_g1	-9.6065	0.006337	XP_008811745
TRINITY_DN78903_c0_g1	-10.6109	0.006337	ADG34846
TRINITY_DN78976_c0_g1	-10.5187	0.006337	XP_008812023
TRINITY_DN79200_c0_g1	-9.66399	0.006337	ABZ80408
TRINITY_DN80099_c0_g1	-2.99484	0.007869	XP_010936924
TRINITY_DN81025_c0_g1	-1.25023	0.015456	ABK22247
TRINITY_DN85195_c0_g1	-1.28597	0.015035	AGW46496
TRINITY_DN85344_c0_g1	-9.42143	0.006337	XP_010922265
TRINITY_DN85361_c0_g1	-10.4351	0.006337	XP_008788265
TRINITY_DN85365_c0_g1	-11.3947	0.006337	WP_017428777
TRINITY_DN85384_c0_g1	-12.0048	0.006337	XP_010255557
TRINITY_DN85440_c0_g1	-1.8661	0.010761	AAU87299
TRINITY_DN85509_c0_g1	-10.2436	0.006337	XP_010248011
TRINITY_DN85913_c0_g1	-10.5916	0.006337	NP_001234786
TRINITY_DN85985_c0_g1	-11.2757	0.006337	ABD66515
TRINITY_DN86066_c0_g1	-12.2389	0.006337	XP_010922267
TRINITY_DN86251_c0_g1	-9.57457	0.006337	XP_010922267
TRINITY_DN86739_c0_g1	-11.0116	0.006337	XP_008783948
TRINITY_DN87476_c0_g1	-8.64483	0.006337	-
TRINITY_DN92482_c0_g1	-5.50007	0.006506	XP_007373464
TRINITY_DN92625_c0_g1	-10.6097	0.006337	XP_011004789
TRINITY_DN92653_c0_g1	-11.8198	0.006337	XP_010251870
TRINITY_DN92698_c0_g1	-11.0794	0.006337	XP_010926040
TRINITY_DN92708_c0_g2	-9.51361	0.006337	EMT27803
TRINITY_DN92897_c0_g1	-9.86421	0.006337	KJB39134
TRINITY_DN94537_c0_g1	-9.19204	0.006337	XP_002277628
TRINITY_DN97184_c0_g1	-1.27253	0.01519	-
TRINITY_DN98724_c0_g1	-1.82947	0.010935	-
TRINITY_DN99434_c0_g1	-12.3605	0.006337	Q9FV99LEC2_CROVR
TRINITY_DN99520_c0_g1	-10.7236	0.006337	XP_007010484
TRINITY_DN99566_c0_g1	-9.72735	0.006337	XP_009408498
TRINITY_DN99660_c0_g1	-11.8138	0.006337	XP_006659552
TRINITY_DN99691_c0_g1	-10.6269	0.006337	XP_010939664
