# Supplementary material for: Comparative transcriptome analysis of Gastrodia elata (Orchidaceae) in response to fungus symbiosis to identify gastrodin biosynthesis-related genes
Source: BMC Genomics. 2016 Mar 9;17:212. doi: 10.1186/s12864-016-2508-6 (PMC4784368; doi:10.1186/s12864-016-2508-6)
Supplement: Additional file 5: Table S3. — Mapping of KEGG biological pathways for up-regulated (log2-FC ≥ 1, q-value < 0. 05, TMM-normalized FPKM > 0.3) unigenes from Armillaria mellea compared to juvenile tuber of G. elata. (PDF 125 kb) [file 12864_2016_2508_MOESM5_ESM.pdf]

Additional file 5: Table S3 Mapping of KEGG biological pathways for up-regulated (log2-FC ≥ 1, *q*-value < 0. 05, TMM-normalized FPKM > 0.3) unigenes from *Armillaria mellea* compared to juvenile tuber of *G. elata.*

Pathway category	Pathway	Number of genes
#Metabolism		
##Global and overview maps		
	Carbon metabolism	3
	Biosynthesis of amino acids	1
##Carbohydrate metabolism		
	Glyoxylate and dicarboxylate metabolism	2
	Galactose metabolism	1
	Glycolysis / Gluconeogenesis	1
##Energy metabolism		
	Oxidative phosphorylation	2
	Carbon fixation in photosynthetic organisms	1
#Genetic Information Processing		
##Transcription		
	Spliceosome	3
##Translation		
	Ribosome	7
##Folding, sorting and degradation		
	Protein processing in endoplasmic reticulum	1
#Environmental Information Processing		
##Signal transduction		
	FoxO signaling pathway	1
	AMPK signaling pathway	1
	MAPK signaling pathway	1
	HIF-1 signaling pathway	1
#Cellular Processes		
##Transport and catabolism		
	Peroxisome	1
	Endocytosis	1
