# Supplementary material for: Comparative transcriptome analysis of Gastrodia elata (Orchidaceae) in response to fungus symbiosis to identify gastrodin biosynthesis-related genes
Source: BMC Genomics. 2016 Mar 9;17:212. doi: 10.1186/s12864-016-2508-6 (PMC4784368; doi:10.1186/s12864-016-2508-6)
Supplement: Additional file 6: Table S4. — Mapping of KEGG biological pathways for down-regulated (log2-FC ≤ -1, q-value < 0.05, TMM-normalized FPKM > 0.3) unigenes from Armillaria mellea compared to juvenile tuber of G. elata. (PDF 130 kb) [file 12864_2016_2508_MOESM6_ESM.pdf]

Additional file 6: Table S4. Mapping of KEGG biological pathways for down-regulated (log2-FC ≤ -1, *q*-value < 0.05, TMM-normalized FPKM > 0.3) unigenes from *Armillaria mellea* compared to juvenile tuber of *G. elata.*

Pathway category	Pathway	Number of genes
#Metabolism		
##Global and overview maps		
	Carbon metabolism	2
	Biosynthesis of amino acids	2
##Carbohydrate metabolism		
	Fructose and mannose metabolism	2
	Glyoxylate and dicarboxylate metabolism	2
	Amino sugar and nucleotide sugar metabolism	2
	Starch and sucrose metabolism	2
	Ascorbate and aldarate metabolism	1
	Pentose and glucuronate interconversions	1
	Inositol phosphate metabolism	1
##Energy metabolism		
	Oxidative phosphorylation	3
	Nitrogen metabolism	1
	Sulfur metabolism	1
##Amino acid metabolism		
	Phenylalanine metabolism	2
	Alanine, aspartate and glutamate metabolism	2
	Tryptophan metabolism	1
	Arginine and proline metabolism	1
	Cysteine and methionine metabolism	1
	Glycine, serine and threonine metabolism	1
##Metabolism of other amino acids		
	Glutathione metabolism	4
	Cyanoamino acid metabolism	1
##Metabolism of cofactors and vitamins		
	Ubiquinone and other terpenoid-quinone biosynthesis	1
##Biosynthesis of other secondary metabolites		
	Phenylpropanoid biosynthesis	3
	Streptomycin biosynthesis	1

##Xenobiotics biodegradation and metabolism		
	Drug metabolism - cytochrome P450	4
	Metabolism of xenobiotics by cytochrome P450	4
#Genetic Information Processing		
##Transcription		
	Spliceosome	6
##Translation		
	RNA transport	9
	Ribosome	9
	mRNA surveillance pathway	2
##Folding, sorting and degradation		
	Protein processing in endoplasmic reticulum	12
	Ubiquitin mediated proteolysis	4
	Proteasome	1
#Environmental Information Processing		
##Signal transduction		
	MAPK signaling pathway	3
	PI3K-Akt signaling pathway	2
	Rap1 signaling pathway	2
	Hippo signaling pathway	2
	Hippo signaling pathway - fly	2
	Phosphatidylinositol signaling system	1
	Ras signaling pathway	1
	Two-component system	1
	Calcium signaling pathway	1
	Notch signaling pathway	1
	TGF-beta signaling pathway	1
	cAMP signaling pathway	1
	Wnt signaling pathway	1
	cGMP-PKG signaling pathway	1
	FoxO signaling pathway	1
	Plant hormone signal transduction	1
#Cellular Processes		
##Transport and catabolism		
	Endocytosis	6
	Phagosome	3
	Peroxisome	2
