# Supplementary material for: Comparative transcriptome analysis of Gastrodia elata (Orchidaceae) in response to fungus symbiosis to identify gastrodin biosynthesis-related genes
Source: BMC Genomics. 2016 Mar 9;17:212. doi: 10.1186/s12864-016-2508-6 (PMC4784368; doi:10.1186/s12864-016-2508-6)
Supplement: Additional file 8: Table S5. — 298 unigenes were significantly up-regulated (log2-FC ≥ 1, q-value < 0.05, TMM-normalized FPKM > 10) in juvenile tubers compared with vegetative propagation corms of Gastrodia elata. (PDF 183 kb) [file 12864_2016_2508_MOESM8_ESM.pdf]

Additional file 8: Table S5. 298 unigenes were significantly up-regulated (log2-FC ≥ 1, *q*-value < 0.05, TMM-normalized FPKM > 10) in juvenile tubers compared with vegetative propagation corms of *Gastrodia elata* .

Unigene id	log2-FC	*q*-value	Hit accession
TRINITY_DN100713_c0_g1	1.639925	0.034039	EPS66415
TRINITY_DN100788_c0_g1	1.224663	0.040371	XP_013632659
TRINITY_DN10993_c0_g2	3.583873	0.025582	XP_009394433
TRINITY_DN12597_c0_g2	1.285946	0.039132	XP_008775490
TRINITY_DN14409_c0_g2	1.175381	0.041812	XP_010907898
TRINITY_DN14758_c0_g1	2.487861	0.028761	XP_009392070
TRINITY_DN14758_c0_g2	2.703543	0.027921	XP_009392070
TRINITY_DN14986_c0_g1	1.379252	0.037388	XP_010942890
TRINITY_DN14986_c0_g2	1.077163	0.044809	XP_010942890
TRINITY_DN15118_c0_g1	1.505575	0.035171	ACN41049
TRINITY_DN15433_c0_g1	1.502324	0.035171	AIZ68155
TRINITY_DN15926_c0_g2	1.296224	0.039057	-
TRINITY_DN1599_c0_g1	1.03512	0.046379	XP_009413850
TRINITY_DN16066_c0_g2	1.773366	0.032289	-
TRINITY_DN16171_c0_g1	1.733832	0.03283	XP_010914939
TRINITY_DN16271_c0_g1	1.859809	0.031309	XP_008776735
TRINITY_DN16416_c0_g1	1.138845	0.042929	XP_009407211
TRINITY_DN16416_c0_g2	1.261429	0.039706	XP_010932192
TRINITY_DN18034_c0_g1	1.50534	0.035171	XP_009380438
TRINITY_DN18299_c0_g2	1.525114	0.035171	XP_010270806
TRINITY_DN191_c0_g2	1.476639	0.035354	XP_008811426
TRINITY_DN19649_c0_g1	1.015004	0.047097	-
TRINITY_DN19791_c0_g1	1.726407	0.03289	XP_006429871
TRINITY_DN20276_c0_g1	1	0.047669	XP_008796730
TRINITY_DN2118_c0_g1	1.5441	0.035171	XP_010514560
TRINITY_DN22997_c0_g1	1.172875	0.041889	XP_010910372
TRINITY_DN23160_c0_g1	1.399857	0.036936	XP_009419273
TRINITY_DN24387_c0_g1	4.363231	0.024894	XP_007043894
TRINITY_DN25216_c0_g2	1.575656	0.035044	XP_008792652
TRINITY_DN25444_c0_g2	1.206223	0.040902	XP_008796849
TRINITY_DN26650_c0_g1	1.737139	0.032787	XP_010251818
TRINITY_DN27072_c0_g1	1.245998	0.040108	XP_010941130
TRINITY_DN27159_c0_g1	1.543379	0.035171	XP_010930042

TRINITY_DN27366_c0_g1	1.251831	0.039967	XP_010935001
TRINITY_DN27808_c0_g1	1.081805	0.044646	XP_008807233
TRINITY_DN28728_c0_g2	1.051233	0.045777	XP_010915439
TRINITY_DN29351_c0_g2	1.004629	0.047509	AGI21023
TRINITY_DN30126_c0_g2	1.407609	0.036775	XP_010939796
TRINITY_DN30599_c0_g2	1.684506	0.033409	ABR53728
TRINITY_DN30800_c0_g1	1.684001	0.033414	AFW87388
TRINITY_DN32552_c0_g2	1.509478	0.035171	XP_010939748
TRINITY_DN32922_c0_g2	2.991575	0.02685	XP_003529476
TRINITY_DN32970_c0_g1	1.214736	0.040653	XP_010938102
TRINITY_DN33963_c0_g1	1.60307	0.03462	XP_008799933
TRINITY_DN34052_c0_g1	1.67071	0.033603	XP_008782287
TRINITY_DN34117_c0_g1	1.437629	0.036151	XP_010519769
TRINITY_DN34152_c0_g1	1.127173	0.043233	XP_010940869
TRINITY_DN35087_c0_g1	1.48764	0.035193	XP_009411381
TRINITY_DN35387_c0_g1	2.15249	0.03077	XP_008792710
TRINITY_DN3590_c0_g1	1.088345	0.044419	XP_009414934
TRINITY_DN36116_c0_g1	1.485233	0.035218	XP_010925933
TRINITY_DN36389_c0_g1	1.143225	0.042852	XP_009412922
TRINITY_DN36907_c0_g1	1.169452	0.041993	AGT78194
TRINITY_DN37628_c0_g1	1.033063	0.046461	AGI60277
TRINITY_DN37941_c0_g1	1.099529	0.044033	XP_010928441
TRINITY_DN38205_c1_g1	1.31118	0.038864	XP_010910900
TRINITY_DN38546_c1_g1	1.49684	0.035171	XP_013601962
TRINITY_DN38770_c1_g1	1.344371	0.038196	XP_008780048
TRINITY_DN38840_c0_g1	1.686151	0.033386	XP_004333525
TRINITY_DN38858_c0_g1	1.431233	0.036278	XP_003548693
TRINITY_DN38858_c1_g1	1.427896	0.036348	-
TRINITY_DN38858_c1_g2	1.281145	0.039186	-
TRINITY_DN39639_c0_g1	1.034341	0.046412	XP_008790305
TRINITY_DN39796_c0_g3	1.526955	0.035171	XP_009400291
TRINITY_DN40624_c0_g1	1.090137	0.044363	-
TRINITY_DN40664_c0_g2	1.41772	0.036564	XP_009414082
TRINITY_DN41033_c0_g1	1.661792	0.033736	-
TRINITY_DN41762_c0_g1	1.073655	0.044933	XP_011098289
TRINITY_DN41762_c0_g3	1.440896	0.036084	XP_002467670
TRINITY_DN42823_c0_g1	1.384044	0.03728	XP_004339805
TRINITY_DN42834_c0_g1	3.38294	0.025693	AAA19578

TRINITY_DN42934_c0_g2	1.178381	0.041724	XP_010917337
TRINITY_DN43172_c0_g1	1.066302	0.045206	XP_008810306
TRINITY_DN43381_c0_g1	2.490092	0.028757	XP_009381432
TRINITY_DN43471_c0_g2	1.719032	0.032946	XP_010932763
TRINITY_DN43472_c0_g1	2.13658	0.030904	XP_010905156
TRINITY_DN44260_c1_g1	1.090403	0.044356	XP_011020467
TRINITY_DN44558_c1_g1	2.268789	0.030169	XP_010920047
TRINITY_DN44785_c0_g1	1.080563	0.04469	XP_006286283
TRINITY_DN44785_c1_g1	1.024526	0.046745	-
TRINITY_DN4489_c0_g2	1.272503	0.039401	XP_007015380
TRINITY_DN45703_c0_g1	1.713358	0.033003	AGW24660
TRINITY_DN46198_c0_g1	1.691451	0.033307	XP_009420693
TRINITY_DN46645_c0_g1	2.206164	0.030305	XP_008813210
TRINITY_DN46645_c0_g2	1.282302	0.039165	XP_009417273
TRINITY_DN46948_c1_g1	1.189353	0.041404	AIE56161
TRINITY_DN46986_c0_g4	2.42606	0.029137	CBI28785
TRINITY_DN47846_c0_g1	1.558451	0.035171	XP_008788132
TRINITY_DN48266_c0_g1	1.802908	0.031937	XP_009391274
TRINITY_DN48632_c2_g1	3.1339	0.02641	XP_008801417
TRINITY_DN48756_c0_g2	1.28806	0.039111	-
TRINITY_DN48882_c1_g1	1.157458	0.042382	AIU48020
TRINITY_DN48960_c0_g1	1.771919	0.032306	XP_008800780
TRINITY_DN49007_c0_g1	1.15623	0.042423	XP_008804656
TRINITY_DN49038_c0_g1	1.721167	0.032925	XP_009420628
TRINITY_DN49061_c0_g1	1.685521	0.033395	XP_006645179
TRINITY_DN49061_c1_g1	1.835522	0.031526	XP_009589046
TRINITY_DN49061_c2_g1	1.549168	0.035171	-
TRINITY_DN49061_c2_g2	1.811105	0.031833	-
TRINITY_DN49061_c3_g1	1.637601	0.034071	XP_013717439
TRINITY_DN49089_c0_g2	1.380925	0.037352	XP_008787999
TRINITY_DN49164_c1_g1	1.10835	0.043793	XP_006430106
TRINITY_DN49358_c0_g1	1.458508	0.035713	XP_010266028
TRINITY_DN49705_c0_g1	1.535792	0.035171	XP_004234444
TRINITY_DN49877_c0_g1	1.314333	0.038803	XP_008789200
TRINITY_DN49901_c0_g1	2.089746	0.031309	XP_010915883
TRINITY_DN50094_c0_g1	1.375135	0.037468	KCW72555
TRINITY_DN50118_c2_g1	1.965913	0.031309	XP_009398286
TRINITY_DN50209_c0_g1	1.11827	0.043503	XP_009403160

TRINITY_DN50227_c0_g1	1.317441	0.038734	AGN70893
TRINITY_DN50741_c0_g3	2.836591	0.027387	XP_010087311
TRINITY_DN50813_c0_g3	1.523717	0.035171	XP_008812796
TRINITY_DN51039_c1_g1	1.301905	0.039001	XP_008803073
TRINITY_DN51689_c0_g2	1.330608	0.038504	XP_010937085
TRINITY_DN51716_c0_g1	1.349921	0.038058	XP_008803933
TRINITY_DN51876_c0_g1	1.149438	0.042648	-
TRINITY_DN51894_c1_g1	1.443245	0.036036	XP_010918671
TRINITY_DN51960_c0_g1	1.102122	0.043956	XP_010923316
TRINITY_DN52456_c0_g1	2.208032	0.030289	XP_008803482
TRINITY_DN52469_c0_g1	1.044198	0.046036	XP_010918752
TRINITY_DN52637_c0_g1	1.939465	0.031309	XP_009409520
TRINITY_DN5269_c0_g1	1.52588	0.035171	XP_010906116
TRINITY_DN52736_c0_g1	2.21076	0.03027	XP_008777796
TRINITY_DN52827_c0_g1	1.463737	0.035608	XP_010936352
TRINITY_DN52892_c0_g2	1.196238	0.04119	XP_008783926
TRINITY_DN52912_c0_g2	1.05366	0.045683	XP_010926686
TRINITY_DN52922_c0_g1	1.789917	0.032089	XP_010934565
TRINITY_DN53023_c0_g1	1.317076	0.038734	KHN15441
TRINITY_DN53023_c0_g2	1.292827	0.039075	XP_013599762
TRINITY_DN53023_c0_g3	1.291767	0.039075	XP_010513507
TRINITY_DN53183_c0_g1	1.16183	0.042239	AAA20899
TRINITY_DN53413_c0_g2	2.040763	0.031309	AGI62058
TRINITY_DN53436_c0_g1	1.361342	0.03778	CAJ29308
TRINITY_DN53476_c0_g1	1.472208	0.035436	XP_008662843
TRINITY_DN53478_c0_g1	1.174375	0.041843	AGI62050
TRINITY_DN53569_c0_g2	1.059861	0.045453	XP_008797904
TRINITY_DN53745_c0_g1	4.542864	0.02471	XP_013709477
TRINITY_DN53745_c0_g3	4.43643	0.024818	ERN16036
TRINITY_DN53745_c0_g4	4.093769	0.025239	XP_012481562
TRINITY_DN53745_c0_g5	4.220792	0.025061	XP_013587907
TRINITY_DN53745_c1_g1	4.260668	0.025014	-
TRINITY_DN53783_c0_g1	1.212519	0.040717	XP_010915492
TRINITY_DN53966_c0_g1	1.566199	0.035171	XP_010925457
TRINITY_DN54082_c1_g1	1.026511	0.046677	CAD22154
TRINITY_DN54282_c0_g1	1.241674	0.040739	XP_010942370
TRINITY_DN54418_c0_g3	1.192394	0.041309	-
TRINITY_DN54561_c0_g2	1.614811	0.034438	XP_010925854

TRINITY_DN55151_c0_g1	1.87378	0.031309	XP_010930878
TRINITY_DN55170_c0_g1	1.14454	0.04281	XP_010053546
TRINITY_DN55289_c1_g3	1.785961	0.032133	
TRINITY_DN55313_c1_g1	1.252112	0.039962	XP_008792589
TRINITY_DN55327_c0_g2	1.63882	0.034052	XP_009394604
TRINITY_DN55491_c2_g1	1.243306	0.040131	XP_010918679
TRINITY_DN55491_c3_g3	1.11985	0.043454	XP_009414927
TRINITY_DN55565_c1_g1	1.078003	0.04478	-
TRINITY_DN55648_c0_g1	1.07404	0.044921	XP_010937050
TRINITY_DN55657_c2_g1	7.512538	0.02354	XP_010911047
TRINITY_DN55709_c0_g1	1.052516	0.045725	XP_008805752
TRINITY_DN55822_c0_g2	1.090745	0.044342	XP_008790910
TRINITY_DN56161_c2_g1	2.092118	0.031309	XP_008806310
TRINITY_DN56319_c0_g1	2.307101	0.029887	AFQ31623
TRINITY_DN56585_c0_g3	1.409998	0.036725	AFW68533
TRINITY_DN56989_c1_g1	1.601343	0.034647	XP_010918079
TRINITY_DN57246_c0_g1	2.283945	0.030067	-
TRINITY_DN57455_c0_g2	1.376732	0.037435	XP_010923269
TRINITY_DN57656_c1_g1	1.816393	0.031767	XP_010917615
TRINITY_DN57811_c1_g1	2.990918	0.026851	XP_009400338
TRINITY_DN58270_c4_g1	1.745239	0.03267	XP_008802983
TRINITY_DN58276_c0_g2	1.149422	0.042648	XP_008809448
TRINITY_DN58298_c6_g2	1.009329	0.047318	XP_010919616
TRINITY_DN58361_c2_g1	1.498069	0.035171	AGK07583
TRINITY_DN58541_c1_g1	1.287071	0.039122	XP_008810895
TRINITY_DN58641_c2_g3	1.196204	0.04119	XP_008799673
TRINITY_DN58653_c0_g2	1.415317	0.036614	XP_003630695
TRINITY_DN58659_c1_g1	1.900863	0.031309	XP_006850002
TRINITY_DN58717_c0_g1	2.031805	0.031309	XP_008455528
TRINITY_DN58763_c0_g1	1.185754	0.041513	XP_002265297
TRINITY_DN58831_c0_g1	1.179865	0.04168	XP_010937594
TRINITY_DN59361_c0_g1	3.64248	0.025582	XP_008776897
TRINITY_DN59564_c1_g1	1.626836	0.03424	XP_010906889
TRINITY_DN59624_c0_g1	1.11802	0.043512	XP_010914678
TRINITY_DN59906_c3_g2	1.002365	0.047604	XP_009421339
TRINITY_DN59932_c0_g1	1.777887	0.032231	XP_008777748
TRINITY_DN60403_c16_g1	1.394567	0.037048	AEG78833
TRINITY_DN60601_c1_g1	1.00144	0.047643	-

TRINITY_DN60670_c7_g2	2.257485	0.030169	XP_008801227
TRINITY_DN60830_c2_g2	1.653589	0.033852	-
TRINITY_DN61089_c4_g1	1.310103	0.038885	XP_010922677
TRINITY_DN61175_c9_g6	1.040408	0.046186	XP_008813036
TRINITY_DN61230_c0_g1	2.880042	0.02722	CDY44804
TRINITY_DN61230_c0_g2	3.006617	0.026795	XP_012069312
TRINITY_DN61462_c6_g2	1.002451	0.047601	-
TRINITY_DN61627_c9_g2	1.007225	0.047401	XP_010932826
TRINITY_DN61656_c0_g1	2.464998	0.028894	XP_010919951
TRINITY_DN61734_c0_g1	1.263272	0.039652	XP_008779870
TRINITY_DN61949_c0_g1	1.834172	0.031544	XP_008800482
TRINITY_DN61977_c0_g1	1.209089	0.040818	-
TRINITY_DN62005_c5_g1	1.185331	0.041526	XP_010943217
TRINITY_DN62057_c0_g1	1.183092	0.041592	-
TRINITY_DN62152_c10_g3	1.903033	0.031309	XP_010927732
TRINITY_DN62167_c1_g6	1.062248	0.045361	XP_010265484
TRINITY_DN62339_c5_g3	1.511467	0.035171	XP_003612269
TRINITY_DN62500_c6_g4	1.022596	0.046807	-
TRINITY_DN62532_c10_g1	1.095772	0.044164	XP_008786726
TRINITY_DN62591_c12_g3	1.393002	0.037079	-
TRINITY_DN62620_c8_g1	1.378595	0.037398	KMT01534
TRINITY_DN62823_c0_g1	1.283918	0.039146	XP_010942728
TRINITY_DN62883_c6_g2	1.485488	0.035216	XP_009392714
TRINITY_DN63163_c0_g1	2.010949	0.031309	XP_008781171
TRINITY_DN63212_c3_g2	1.719971	0.032938	-
TRINITY_DN63365_c3_g2	1.138848	0.042929	-
TRINITY_DN63485_c5_g1	1.402735	0.036876	XP_012853719
TRINITY_DN63724_c0_g1	1.269206	0.039485	XP_010939224
TRINITY_DN63869_c0_g1	1.016197	0.047046	EKV05924
TRINITY_DN63929_c0_g1	1.329053	0.038536	ABC02343
TRINITY_DN64101_c0_g1	3.471943	0.025582	XP_010912492
TRINITY_DN64125_c0_g1	2.041027	0.031309	XP_009408845
TRINITY_DN64137_c0_g1	1.554525	0.035171	XP_009398603
TRINITY_DN64574_c0_g1	1.14378	0.042835	XP_002519430
TRINITY_DN64626_c0_g1	1.115124	0.043595	XP_009391366
TRINITY_DN65255_c0_g1	1.321622	0.038661	XP_010927360
TRINITY_DN65487_c0_g1	1.566385	0.035171	XP_011100211
TRINITY_DN65649_c0_g1	1.419762	0.036523	XP_008779090

TRINITY_DN65747_c0_g1	1.592413	0.034774	XP_009390323
TRINITY_DN66292_c0_g1	2.711528	0.027891	XP_008466730
TRINITY_DN67104_c0_g1	1.622623	0.034312	-
TRINITY_DN67612_c0_g1	1.740471	0.032739	-
TRINITY_DN70645_c0_g1	1.362419	0.037754	XP_008787474
TRINITY_DN70658_c0_g1	3.035879	0.026695	XP_008370710
TRINITY_DN70666_c0_g1	3.814968	0.025582	XP_004984672
**TRINITY_DN70668_c0_g1**	**2.840148**	**0.027375**	**AAG52664;AF225410_1**
TRINITY_DN70672_c0_g1	1.943036	0.031309	ACJ02103
TRINITY_DN70689_c0_g1	1.881621	0.031309	XP_010927393
TRINITY_DN70692_c0_g1	1.209572	0.040804	AFS50097
TRINITY_DN70693_c0_g1	3.993001	0.025388	XP_008793269
TRINITY_DN70714_c0_g1	1.635107	0.034108	XP_009388359
TRINITY_DN70762_c0_g1	1.684461	0.033409	XP_008669428
TRINITY_DN70819_c0_g1	1.131817	0.043106	XP_008810568
TRINITY_DN71094_c0_g1	1.70384	0.033126	XP_010928985
TRINITY_DN71282_c0_g1	1.112366	0.043667	AIB06955
TRINITY_DN71339_c0_g1	1.00198	0.047621	XP_009380624
TRINITY_DN71395_c0_g1	2.625192	0.028313	XP_010917262
TRINITY_DN71530_c0_g1	1.370198	0.037577	XP_008782717
TRINITY_DN71589_c0_g1	2.249037	0.030169	XP_010911301
TRINITY_DN7158_c0_g1	1.151663	0.042574	-
TRINITY_DN71676_c0_g1	2.788039	0.0276	ABG27026
TRINITY_DN71752_c0_g1	1.175524	0.041808	XP_010924297
TRINITY_DN7542_c0_g1	4.251769	0.025026	WP_047447838
TRINITY_DN7702_c0_g1	2.09243	0.031309	XP_008461081
TRINITY_DN7730_c0_g1	1.719342	0.032942	XP_010943376
TRINITY_DN77933_c0_g1	1.105082	0.043883	XP_012573034
TRINITY_DN78024_c0_g1	3.667757	0.025582	AAM28293
TRINITY_DN78066_c0_g1	1.260386	0.039734	XP_010929910
TRINITY_DN78089_c0_g1	1.183356	0.041585	XP_009399660
TRINITY_DN78195_c0_g1	1.285425	0.039134	XP_010929163
TRINITY_DN78300_c0_g1	1.951575	0.031309	XP_010939659
TRINITY_DN78323_c0_g1	1.723139	0.032912	NP_001149596
TRINITY_DN78587_c0_g1	2.376811	0.029458	XP_002520835
TRINITY_DN78900_c0_g1	1.103789	0.043921	ABC55716
TRINITY_DN78943_c0_g1	1.059888	0.045453	XP_008802404
TRINITY_DN79010_c0_g1	1.007312	0.047398	XP_008791035

TRINITY_DN7941_c0_g1	1.032081	0.046491	XP_008803456
TRINITY_DN80221_c0_g1	2.558841	0.028534	XP_008229501
TRINITY_DN81528_c0_g1	1.095297	0.044179	XP_012073949
TRINITY_DN82133_c0_g1	1.383854	0.037284	CKX19056
TRINITY_DN83656_c0_g1	1.00699	0.04741	XP_008806985
TRINITY_DN85384_c0_g1	1.656899	0.033812	XP_010255557
TRINITY_DN85392_c0_g1	3.324323	0.025835	XP_008804619
TRINITY_DN85541_c0_g1	2.75442	0.027754	XP_003555937
TRINITY_DN85591_c0_g1	2.92405	0.027104	XP_010919404
TRINITY_DN85614_c0_g1	1.45886	0.035707	XP_009379971
TRINITY_DN85736_c0_g1	1.469116	0.035497	XP_010922574
TRINITY_DN85739_c0_g1	1.724416	0.032904	XP_010921012
TRINITY_DN85771_c0_g1	1.977177	0.031309	XP_009397426
TRINITY_DN86028_c0_g2	1.407062	0.036787	XP_010919401
TRINITY_DN86037_c0_g1	1.379178	0.037389	XP_008796816
TRINITY_DN86800_c0_g1	1.209786	0.0408	AFS50010
TRINITY_DN87777_c0_g1	1.739464	0.032752	EAZ38663
TRINITY_DN9015_c0_g1	1.077898	0.044782	XP_009417164
TRINITY_DN92392_c0_g1	1.368615	0.037613	XP_009420139
TRINITY_DN92479_c0_g1	1.838395	0.031497	XP_010942073
TRINITY_DN92653_c0_g1	1.95822	0.031309	XP_010251870
TRINITY_DN93146_c0_g1	1.522056	0.035171	XP_010246607
TRINITY_DN93367_c0_g1	4.507577	0.024744	XP_009402509
TRINITY_DN93440_c0_g1	1.251993	0.039964	XP_008811258
TRINITY_DN94167_c0_g1	3.067907	0.026586	XP_008783548
TRINITY_DN94738_c0_g1	1.895222	0.031309	XP_010937168
TRINITY_DN95264_c0_g1	1.267853	0.039525	XP_008800094
TRINITY_DN9580_c0_g1	1.352203	0.038005	XP_010933529
TRINITY_DN99434_c0_g1	3.67487	0.025582	Q9FV99LEC2_CROVR
TRINITY_DN99439_c0_g1	3.228688	0.026111	XP_008777642
TRINITY_DN99470_c0_g1	1.319952	0.038701	XP_008785941
TRINITY_DN99473_c0_g1	1.183711	0.041574	XP_004968074
TRINITY_DN99603_c0_g1	2.029851	0.031309	-
TRINITY_DN99660_c0_g1	2.379791	0.029439	XP_006659552
TRINITY_DN99850_c0_g1	1.437519	0.036153	XP_010937940
TRINITY_DN99884_c0_g1	1.508463	0.035171	ERN17394
TRINITY_DN99887_c0_g1	1.037775	0.046283	XP_008798819
