# Supplementary material for: Comparative transcriptome analysis of Gastrodia elata (Orchidaceae) in response to fungus symbiosis to identify gastrodin biosynthesis-related genes
Source: BMC Genomics. 2016 Mar 9;17:212. doi: 10.1186/s12864-016-2508-6 (PMC4784368; doi:10.1186/s12864-016-2508-6)
Supplement: Additional file 9: Table S6. — 405 unigenes were significantly down-regulated (log2-FC ≤ -1, q-value < 0.05, TMM-normalized FPKM > 10) in juvenile tubers compared with vegetative propagation corms of Gastrodia elata. (PDF 168 kb) [file 12864_2016_2508_MOESM9_ESM.pdf]

Additional file 9: Table S6. 405 unigenes were significantly down-regulated (log2-FC ≤ -1, *q*-value < 0.05, TMM-normalized FPKM > 10) in juvenile tubers compared with vegetative propagation corms of *Gastrodia elata*.

Unigene id	log2-FC	*q*-value	Hit accession
TRINITY_DN100190_c0_g1	-1.69666	0.03130923	XP_009403415
TRINITY_DN100498_c0_g1	-1.18392	0.04904615	XP_012462455
TRINITY_DN100727_c0_g1	-1.1737	0.04989028	
TRINITY_DN101396_c0_g1	-1.3215	0.04292178	
TRINITY_DN105655_c0_g1	-2.56608	0.02352708	
TRINITY_DN1125_c0_g2	-1.50065	0.03517105	XP_009380990
TRINITY_DN11803_c1_g1	-1.48545	0.0356672	XP_010932564
TRINITY_DN13036_c0_g1	-1.28598	0.0448629	XP_008791269
TRINITY_DN13256_c0_g2	-1.39207	0.03941393	XP_008783619
TRINITY_DN13755_c0_g1	-2.10713	0.02558154	XP_008779112
TRINITY_DN14116_c0_g1	-1.65434	0.03135345	
TRINITY_DN14409_c0_g1	-1.77479	0.03045468	XP_010907898
TRINITY_DN14656_c0_g1	-1.30311	0.04384604	XP_009420419
TRINITY_DN15893_c0_g1	-1.84945	0.02910495	XP_010905948
TRINITY_DN15968_c0_g1	-1.57989	0.03360308	XP_010922601
TRINITY_DN16005_c0_g3	-1.6409	0.03173921	XP_010943207
TRINITY_DN16005_c0_g4	-1.82348	0.02965116	XP_010943207
TRINITY_DN16109_c0_g1	-2.01598	0.0264194	KDO74541
TRINITY_DN16125_c0_g1	-1.22186	0.04884464	XP_010942045
TRINITY_DN16136_c0_g2	-1.21463	0.04884464	AAB80714
TRINITY_DN16171_c0_g3	-1.25591	0.04679492	XP_010914939
TRINITY_DN16367_c0_g1	-1.19794	0.04884464	XP_010924632
TRINITY_DN16393_c0_g1	-1.26023	0.04654953	XP_009407672
TRINITY_DN16393_c0_g2	-1.28793	0.04474089	XP_008792606
TRINITY_DN16454_c0_g1	-1.48364	0.03574204	XP_008791806
TRINITY_DN1675_c0_g1	-1.20729	0.04884464	XP_008811830
TRINITY_DN18816_c0_g2	-2.58679	0.02352708	ADK74340
TRINITY_DN18938_c0_g1	-1.2967	0.04420125	XP_008801109
TRINITY_DN18971_c0_g1	-1.46156	0.03670732	XP_009400437
TRINITY_DN19124_c0_g1	-1.29427	0.0443577	
TRINITY_DN1920_c0_g2	-1.3184	0.04306434	KJB35265
TRINITY_DN1966_c0_g1	-1.53337	0.03517105	XP_009778111
TRINITY_DN19678_c0_g1	-1.65057	0.03143873	

TRINITY_DN20546_c0_g1	-1.51512	0.03517105	XP_010914078
TRINITY_DN20747_c0_g1	-2.42798	0.02352708	XP_008795809
TRINITY_DN22295_c0_g1	-1.24637	0.04742761	XP_008808288
TRINITY_DN22456_c0_g1	-1.60872	0.03275433	XP_010911822
TRINITY_DN23245_c0_g1	-2.40603	0.02352708	XP_010266134
TRINITY_DN23481_c0_g2	-1.33371	0.04223981	XP_010926728
TRINITY_DN24520_c0_g1	-1.79752	0.03016905	XP_008795208
TRINITY_DN25897_c0_g2	-1.21415	0.04884464	XP_008796609
TRINITY_DN2611_c0_g1	-2.20258	0.02465862	XP_010914931
TRINITY_DN26968_c0_g2	-1.34781	0.04145128	XP_008808375
TRINITY_DN27041_c0_g2	-1.72104	0.03130923	XP_008799546
TRINITY_DN27046_c0_g1	-1.54374	0.03484616	XP_009387750
TRINITY_DN27270_c0_g1	-1.2426	0.04766859	XP_010908363
TRINITY_DN27683_c0_g2	-1.30122	0.043942	XP_010908490
TRINITY_DN27741_c0_g1	-1.26036	0.04654012	XP_008795725
TRINITY_DN28094_c0_g2	-1.83141	0.02950138	XP_010925425
TRINITY_DN28420_c0_g2	-1.60644	0.03282992	XP_010914285
TRINITY_DN28529_c0_g1	-1.42901	0.03819033	XP_008806831
TRINITY_DN28945_c0_g1	-2.57456	0.02352708	XP_008779774
TRINITY_DN29408_c0_g1	-1.373	0.04016228	AGI62046
TRINITY_DN30047_c0_g1	-1.25376	0.04693657	XP_004488942
TRINITY_DN30353_c0_g1	-1.39548	0.03924785	XP_010241296
TRINITY_DN30750_c0_g1	-2.06192	0.02568594	XP_010917623
TRINITY_DN30859_c0_g2	-1.23277	0.04842616	XP_010942683
TRINITY_DN30888_c0_g1	-1.76571	0.03069891	AEX56222
TRINITY_DN31233_c0_g2	-1.70759	0.03130923	XP_009406308
TRINITY_DN31341_c0_g2	-1.87589	0.02871172	KMZ68638
TRINITY_DN32630_c0_g1	-1.33626	0.04209105	XP_008805432
TRINITY_DN32688_c0_g2	-1.28425	0.04497404	
TRINITY_DN33022_c0_g1	-1.22362	0.04884464	XP_008785598
TRINITY_DN33339_c0_g1	-1.63003	0.03207329	XP_010258037
TRINITY_DN33467_c0_g3	-1.50561	0.03517105	XP_010933407
TRINITY_DN33625_c1_g1	-3.48985	0.02352708	XP_008780830
TRINITY_DN34046_c0_g3	-1.35492	0.04104904	XP_008784439
TRINITY_DN34049_c0_g1	-1.28976	0.04463033	XP_012847790
TRINITY_DN35238_c0_g1	-1.6701	0.03130923	XP_004493883
TRINITY_DN35268_c1_g1	-1.46092	0.03673257	AJG01145
TRINITY_DN35308_c0_g2	-1.89661	0.02838713	XP_010258573

TRINITY_DN35495_c0_g1	-1.88995	0.02847866	XP_012486676
TRINITY_DN35650_c1_g1	-1.93983	0.02765307	XP_008792862
TRINITY_DN36381_c0_g2	-1.33323	0.04227011	XP_009412591
TRINITY_DN36571_c0_g1	-2.70966	0.02352708	
TRINITY_DN36571_c0_g2	-2.35977	0.02352708	
TRINITY_DN36835_c0_g2	-1.5117	0.03517105	
TRINITY_DN37101_c0_g1	-1.41321	0.03882967	AIZ68134
TRINITY_DN37627_c0_g1	-1.23018	0.04861471	XP_008786118
TRINITY_DN37726_c1_g1	-1.84433	0.02921045	XP_008785419
TRINITY_DN37882_c0_g1	-1.65721	0.03130923	
TRINITY_DN38026_c0_g2	-1.48855	0.03553329	XP_008805610
TRINITY_DN38147_c0_g2	-2.82297	0.02352708	XP_008792572
TRINITY_DN38148_c0_g1	-1.23227	0.04846354	XP_008796127
TRINITY_DN38334_c0_g1	-2.16145	0.02517544	
TRINITY_DN38627_c0_g1	-1.76486	0.03071995	XP_010926928
TRINITY_DN38663_c0_g1	-1.26403	0.04630934	XP_010663946
TRINITY_DN38753_c0_g1	-1.25629	0.04677165	XP_008783404
TRINITY_DN38765_c0_g1	-2.24056	0.02419258	XP_009395469
TRINITY_DN38856_c0_g1	-1.36204	0.04065731	XP_006344662
TRINITY_DN38856_c2_g1	-1.44426	0.03746341	
TRINITY_DN38921_c0_g2	-2.57835	0.02352708	XP_010928608
TRINITY_DN38998_c0_g2	-1.42958	0.03816175	XP_010935539
TRINITY_DN39183_c0_g1	-1.45905	0.03681541	XP_008791913
TRINITY_DN39639_c1_g1	-1.6547	0.03134564	
TRINITY_DN40290_c0_g1	-1.36303	0.04060217	XP_009407353
TRINITY_DN40362_c0_g1	-1.28079	0.0452039	XP_008787779
TRINITY_DN40362_c0_g2	-1.54953	0.03465281	XP_010941596
TRINITY_DN40596_c0_g1	-2.20607	0.02461697	XP_010935251
TRINITY_DN41002_c0_g1	-1.76231	0.0307798	AIS35930
TRINITY_DN41059_c0_g1	-2.1216	0.02558154	XP_008812736
TRINITY_DN41115_c0_g1	-1.55097	0.03460429	XP_008792916
TRINITY_DN42115_c0_g2	-1.23898	0.04795633	XP_009401168
TRINITY_DN42759_c0_g1	-2.06707	0.02561975	XP_003558432
TRINITY_DN43116_c0_g1	-1.38336	0.03988143	XP_009387998
TRINITY_DN43164_c0_g2	-1.31856	0.04305684	XP_009415024
TRINITY_DN43518_c0_g1	-1.79907	0.03016905	XP_010918244
TRINITY_DN44222_c0_g1	-1.82762	0.02957316	XP_008801542
TRINITY_DN44682_c0_g1	-2.0985	0.02558154	XP_009399078

TRINITY_DN44829_c0_g1	-1.70657	0.03130923	
TRINITY_DN44890_c0_g1	-1.32596	0.04271072	XP_009597213
TRINITY_DN45048_c0_g1	-1.45202	0.0371184	XP_008791235
TRINITY_DN45782_c0_g1	-1.27047	0.04588194	XP_008794313
TRINITY_DN45835_c0_g2	-1.28164	0.04514641	XP_010938325
TRINITY_DN4595_c0_g1	-2.06221	0.02568122	
TRINITY_DN46037_c0_g2	-1.52234	0.03517105	XP_008796761
TRINITY_DN46219_c0_g1	-1.2004	0.04884464	XP_010938812
TRINITY_DN47561_c0_g1	-1.9842	0.02691318	
TRINITY_DN47983_c0_g1	-1.3274	0.04262406	XP_009381595
TRINITY_DN48119_c0_g1	-1.2016	0.04884464	XP_008796138
TRINITY_DN48183_c0_g1	-1.32857	0.04255228	XP_009388576
TRINITY_DN48212_c0_g1	-1.66297	0.03130923	XP_010916734
TRINITY_DN48564_c0_g1	-1.28048	0.04522037	XP_008798040
TRINITY_DN48600_c0_g1	-1.57454	0.03379325	XP_008778966
TRINITY_DN48656_c0_g1	-1.19514	0.04884464	XP_010267939
TRINITY_DN48828_c0_g2	-2.0894	0.02558154	XP_008800636
TRINITY_DN49004_c2_g1	-1.41446	0.0387662	
TRINITY_DN49105_c0_g1	-1.49359	0.03532713	XP_008810258
TRINITY_DN49543_c1_g1	-1.9039	0.02828747	XP_010908606
TRINITY_DN49548_c0_g1	-1.28268	0.04507965	XP_009398590
TRINITY_DN49568_c2_g2	-1.41948	0.03859447	XP_010915190
TRINITY_DN49660_c0_g1	-2.29409	0.02363567	XP_010926206
TRINITY_DN50037_c0_g1	-1.77358	0.03048978	
TRINITY_DN50259_c0_g1	-1.54385	0.03484435	XP_010918554
TRINITY_DN50357_c0_g1	-1.453	0.03707485	XP_009407936
TRINITY_DN50491_c0_g1	-1.77032	0.0305741	XP_008809638
TRINITY_DN50544_c0_g2	-1.669	0.03130923	XP_008799157
TRINITY_DN50600_c1_g1	-1.6828	0.03130923	ABD61227
TRINITY_DN50676_c0_g1	-1.35619	0.0409799	XP_009388315
TRINITY_DN50676_c2_g1	-1.22406	0.04884464	XP_010932694
TRINITY_DN50731_c0_g1	-1.30225	0.04389832	XP_008799798
TRINITY_DN50738_c0_g1	-1.75332	0.03101205	XP_008775334
TRINITY_DN50744_c0_g1	-1.21632	0.04884464	XP_010278836
TRINITY_DN50797_c0_g1	-1.24232	0.04768895	XP_010923190
TRINITY_DN50861_c0_g2	-1.3572	0.04092589	XP_010933875
TRINITY_DN50901_c0_g1	-1.27554	0.04555138	XP_009387788
TRINITY_DN50911_c1_g1	-1.31223	0.04337123	XP_010917383

TRINITY_DN51010_c0_g1	-1.42091	0.03854089	XP_012850886
TRINITY_DN51034_c1_g1	-2.39694	0.02352708	XP_008792147
TRINITY_DN51034_c3_g1	-2.27974	0.0237821	XP_009410342
TRINITY_DN51113_c0_g2	-1.49494	0.03527817	
TRINITY_DN51114_c0_g2	-1.34142	0.0417934	XP_009421396
TRINITY_DN51720_c0_g1	-1.94542	0.02754809	XP_008813473
TRINITY_DN51824_c0_g2	-1.44567	0.03740366	XP_008800080
TRINITY_DN51993_c0_g1	-1.28256	0.04508734	XP_010904858
TRINITY_DN52162_c0_g2	-2.70882	0.02352708	AIZ68166
TRINITY_DN52202_c0_g1	-1.18372	0.04904927	
TRINITY_DN52322_c0_g1	-2.06739	0.02561972	
TRINITY_DN52322_c0_g2	-1.31621	0.04316597	
TRINITY_DN52388_c1_g1	-1.19985	0.04884464	
TRINITY_DN52406_c0_g1	-1.21369	0.04884464	XP_008805121
TRINITY_DN52467_c0_g1	-1.31618	0.04316671	XP_008789064
TRINITY_DN524_c0_g1	-1.18114	0.04924685	XP_010917791
TRINITY_DN52508_c0_g1	-1.39529	0.03925489	XP_004961326
TRINITY_DN52508_c1_g1	-1.62831	0.03211714	XP_009393568
TRINITY_DN52660_c0_g1	-1.32053	0.04295886	XP_008775348
TRINITY_DN52675_c0_g1	-1.59831	0.03299356	XP_010244253
TRINITY_DN52692_c0_g1	-1.17507	0.0497746	
TRINITY_DN52697_c0_g3	-1.83392	0.0294497	XP_010940867
TRINITY_DN52812_c0_g2	-1.43294	0.03799687	XP_008789094
TRINITY_DN52871_c0_g1	-1.4717	0.03626774	XP_010923125
TRINITY_DN52940_c0_g1	-1.55348	0.03451825	DAA60408
TRINITY_DN52969_c0_g1	-1.85883	0.0288985	XP_008791967
TRINITY_DN53080_c0_g3	-1.36527	0.04047968	AJD80258
TRINITY_DN53153_c1_g1	-1.36207	0.04065646	NP_001049510
TRINITY_DN53235_c0_g1	-1.86454	0.02877336	XP_009408038
TRINITY_DN53235_c0_g2	-1.3456	0.04157391	XP_009408038
TRINITY_DN53303_c0_g1	-1.46559	0.03653507	XP_009361508
TRINITY_DN53319_c0_g1	-1.41021	0.03892939	XP_010933476
TRINITY_DN53418_c0_g1	-1.40213	0.03907466	XP_008654556
TRINITY_DN53423_c0_g3	-1.17908	0.04942233	
TRINITY_DN53497_c0_g1	-1.23861	0.04798646	XP_008795352
TRINITY_DN53517_c0_g1	-1.3634	0.04058176	XP_010922646
TRINITY_DN53536_c0_g1	-2.44174	0.02352708	XP_009418657
TRINITY_DN53554_c0_g1	-1.17815	0.04949941	XP_008793972

TRINITY_DN53581_c0_g1	-1.37397	0.04015686	
TRINITY_DN53747_c0_g1	-1.19603	0.04884464	XP_009396650
TRINITY_DN53747_c1_g1	-1.35786	0.04089082	XP_009411213
TRINITY_DN53882_c0_g1	-1.24629	0.04743225	XP_008791563
TRINITY_DN53930_c0_g1	-1.21268	0.04884464	XP_010922777
TRINITY_DN53930_c0_g2	-1.18116	0.04924589	XP_010922777
TRINITY_DN54055_c0_g1	-1.36409	0.04054531	NP_001176041
TRINITY_DN54080_c1_g1	-2.21072	0.02456074	XP_008806688
TRINITY_DN54080_c1_g2	-2.79875	0.02352708	CAN80239
TRINITY_DN54272_c0_g2	-1.25926	0.04660573	XP_010253963
TRINITY_DN54301_c0_g1	-3.18537	0.02352708	XP_010244875
TRINITY_DN54621_c0_g1	-1.47307	0.03620857	XP_010938570
TRINITY_DN54624_c1_g1	-2.80015	0.02352708	XP_010911246
TRINITY_DN54624_c1_g3	-3.68032	0.02332788	XP_010911246
TRINITY_DN54624_c1_g4	-2.55108	0.02352708	XP_010911246
TRINITY_DN54624_c3_g1	-3.84987	0.02283301	XP_010911246
TRINITY_DN54785_c1_g1	-1.68178	0.03130923	
TRINITY_DN54926_c1_g1	-2.39601	0.02352708	XP_010534893
TRINITY_DN55008_c0_g1	-1.69905	0.03130923	XP_013746138
TRINITY_DN55032_c5_g1	-1.59681	0.03303485	
TRINITY_DN55105_c0_g5	-1.99083	0.02679751	XP_006648402
TRINITY_DN55192_c0_g1	-1.6031	0.0328964	XP_010914895
TRINITY_DN55200_c0_g1	-1.75752	0.03090203	XP_010907563
TRINITY_DN55200_c1_g1	-1.27998	0.04525264	ABF70146
TRINITY_DN55211_c0_g3	-1.30656	0.04366732	XP_009390484
TRINITY_DN55230_c2_g1	-1.39895	0.03913406	XP_008808045
TRINITY_DN55297_c0_g1	-1.46459	0.03657745	XP_010919498
TRINITY_DN55417_c3_g4	-1.27059	0.04587629	XP_008782184
TRINITY_DN55522_c1_g1	-1.19488	0.04884464	XP_008795735
TRINITY_DN55709_c0_g2	-1.46561	0.03653507	XP_006643716
TRINITY_DN55761_c0_g2	-1.28977	0.04463026	XP_008801216
TRINITY_DN55819_c0_g2	-1.9372	0.02770153	XP_010914891
TRINITY_DN55901_c1_g1	-1.46383	0.03661108	XP_010941741
TRINITY_DN55929_c2_g3	-1.423	0.03846542	XP_010943221
TRINITY_DN56007_c0_g2	-1.56951	0.0339394	XP_010923085
TRINITY_DN56009_c1_g1	-3.15999	0.02352708	XP_010907652
TRINITY_DN56009_c1_g2	-1.26186	0.0464524	XP_010907652
TRINITY_DN56009_c4_g1	-1.36723	0.04037952	XP_006845257

TRINITY_DN56061_c0_g1	-1.95198	0.02742494	XP_010938619
TRINITY_DN56139_c0_g1	-1.32488	0.04277853	XP_010939751
TRINITY_DN56180_c0_g2	-1.28241	0.04509718	XP_010921098
TRINITY_DN56231_c0_g1	-2.01888	0.02636996	XP_008787478
TRINITY_DN56362_c0_g1	-2.23987	0.02420132	XP_008796338
TRINITY_DN56380_c0_g1	-1.94357	0.02758196	XP_008782898
TRINITY_DN56415_c0_g1	-2.07995	0.02558154	XP_008802139
TRINITY_DN56455_c0_g1	-1.26916	0.04596755	XP_008795142
TRINITY_DN56585_c0_g2	-1.57353	0.03382521	XP_008793701
TRINITY_DN56650_c0_g2	-1.74133	0.03130923	XP_008788451
TRINITY_DN56650_c4_g1	-2.48689	0.02352708	XP_008782325
TRINITY_DN56813_c2_g2	-1.51584	0.03517105	
TRINITY_DN56973_c3_g1	-1.26623	0.04617057	ABC55715
TRINITY_DN57045_c0_g2	-1.61882	0.03241647	XP_010934362
TRINITY_DN57205_c1_g1	-1.45536	0.03697214	XP_010936096
TRINITY_DN57211_c1_g3	-2.06614	0.02563499	XP_010929278
TRINITY_DN57211_c3_g1	-2.04081	0.02601281	XP_010913679
TRINITY_DN57340_c0_g2	-1.23355	0.04836618	XP_002525660
TRINITY_DN57378_c0_g2	-1.72286	0.03130923	XP_008811947
TRINITY_DN57407_c0_g1	-1.32881	0.04253847	XP_008813541
TRINITY_DN57531_c0_g2	-1.3446	0.041627	XP_009406286
TRINITY_DN57626_c0_g1	-1.37759	0.04012973	XP_010923144
TRINITY_DN57705_c0_g1	-2.34153	0.02352708	XP_010256849
TRINITY_DN57762_c1_g2	-2.11113	0.02558154	XP_008797589
TRINITY_DN57910_c0_g1	-2.40566	0.02352708	XP_008803948
TRINITY_DN57970_c0_g1	-1.24765	0.04734083	XP_006648478
TRINITY_DN58199_c0_g1	-1.90128	0.02832935	XP_009414463
TRINITY_DN58270_c2_g2	-1.27416	0.04564367	XP_008801552
TRINITY_DN58270_c5_g1	-2.00269	0.0265983	
TRINITY_DN58298_c10_g1	-1.34466	0.04162553	
TRINITY_DN58351_c5_g1	-1.29297	0.04443171	XP_006582340
TRINITY_DN583_c0_g1	-1.2418	0.04772929	XP_003613866
TRINITY_DN58443_c2_g1	-1.34405	0.04165507	
TRINITY_DN58603_c1_g1	-1.19313	0.04884464	XP_010273499
TRINITY_DN58761_c0_g3	-1.1751	0.04977344	XP_010923823
TRINITY_DN58798_c3_g1	-1.61964	0.03238772	XP_010925291
TRINITY_DN58832_c3_g1	-1.38248	0.03992477	
TRINITY_DN58914_c0_g4	-1.20147	0.04884464	

TRINITY_DN58943_c1_g1	-2.26112	0.02398887	
TRINITY_DN58943_c1_g2	-2.2986	0.02357966	
TRINITY_DN59090_c0_g1	-1.34583	0.0415624	XP_010935791
TRINITY_DN59090_c1_g1	-1.32814	0.04257766	
TRINITY_DN59302_c0_g1	-1.38211	0.03994403	XP_010939670
TRINITY_DN59377_c2_g2	-1.23413	0.04832412	XP_008783544
TRINITY_DN59397_c0_g1	-2.24773	0.02410885	XP_009388290
TRINITY_DN59423_c15_g1	-1.78174	0.03027601	XP_010936610
TRINITY_DN59561_c2_g1	-1.23163	0.04850895	ADB81911
TRINITY_DN59561_c3_g1	-1.31967	0.04300195	XP_008787821
TRINITY_DN59707_c11_g3	-1.78383	0.03022717	
TRINITY_DN59744_c1_g1	-2.1779	0.02496179	XP_009417291
TRINITY_DN59820_c0_g1	-1.86306	0.02880061	XP_008787873
TRINITY_DN59933_c1_g2	-1.30407	0.04380389	XP_008798317
TRINITY_DN60133_c6_g1	-1.60048	0.03294248	
TRINITY_DN60143_c9_g1	-1.67172	0.03130923	
TRINITY_DN60175_c4_g1	-1.68238	0.03130923	
TRINITY_DN60183_c11_g1	-3.08273	0.02352708	AAQ55798
TRINITY_DN60259_c0_g5	-1.84783	0.02913913	XP_010943599
TRINITY_DN60301_c0_g1	-1.31919	0.04302602	XP_008798584
TRINITY_DN60307_c2_g1	-1.75945	0.03085551	
TRINITY_DN60307_c2_g2	-1.5468	0.03474201	
TRINITY_DN60554_c0_g2	-1.21889	0.04884464	XP_010939001
TRINITY_DN60659_c0_g1	-1.31761	0.04309505	
TRINITY_DN60716_c3_g1	-1.56979	0.03393363	XP_007137892
TRINITY_DN60716_c4_g1	-1.81937	0.02972917	XP_004968705
TRINITY_DN60882_c17_g1	-1.49453	0.03529289	
TRINITY_DN60882_c8_g9	-1.55387	0.03450461	
TRINITY_DN60980_c3_g3	-1.18176	0.04919797	XP_008791152
TRINITY_DN61125_c8_g2	-1.71632	0.03130923	XP_009394770
TRINITY_DN61138_c0_g1	-2.85711	0.02352708	XP_010917011
TRINITY_DN61175_c9_g5	-1.19649	0.04884464	XP_008813036
TRINITY_DN61227_c4_g1	-1.18754	0.04884979	XP_008781230
TRINITY_DN61257_c3_g2	-1.64044	0.03175454	XP_002468605
TRINITY_DN61257_c6_g1	-1.2579	0.04667821	
TRINITY_DN61265_c1_g3	-1.18604	0.04891297	XP_006358601
TRINITY_DN61291_c1_g3	-1.39827	0.03914604	XP_008805754
TRINITY_DN61291_c6_g1	-1.65478	0.03134351	XP_009398735

TRINITY_DN61295_c10_g1	-1.27135	0.04583145	AEZ49509
TRINITY_DN61309_c2_g1	-1.66857	0.03130923	XP_007047137
TRINITY_DN61353_c0_g3	-1.73604	0.03130923	XP_010246318
TRINITY_DN61492_c1_g1	-2.40379	0.02352708	
TRINITY_DN61492_c7_g1	-2.32351	0.02352708	XP_013708284
TRINITY_DN61610_c5_g2	-2.80531	0.02352708	XP_008810393
TRINITY_DN61734_c11_g5	-1.1815	0.04921774	
TRINITY_DN61851_c4_g2	-1.61389	0.0325866	
TRINITY_DN61901_c0_g1	-1.2568	0.04674549	XP_010933130
TRINITY_DN61901_c0_g3	-1.26861	0.04600316	XP_008777998
TRINITY_DN62005_c12_g1	-1.26836	0.04601886	XP_010933089
TRINITY_DN62014_c7_g7	-1.87472	0.028735	
TRINITY_DN62124_c7_g2	-1.48624	0.0356345	XP_003579991
TRINITY_DN62133_c4_g11	-1.74398	0.0312687	KDO71656
TRINITY_DN62133_c5_g3	-1.40878	0.03897521	XP_010938163
TRINITY_DN62243_c0_g1	-1.91828	0.02799819	AIM58716
TRINITY_DN62296_c5_g2	-1.18851	0.04884464	NP_001159140
TRINITY_DN62358_c6_g1	-1.33909	0.04192252	XP_010919989
TRINITY_DN62384_c0_g1	-1.2949	0.04432011	
TRINITY_DN62394_c2_g2	-1.2552	0.04683859	KNA12247
TRINITY_DN62396_c5_g3	-2.00764	0.02654412	
TRINITY_DN62409_c1_g1	-1.17694	0.04960935	
TRINITY_DN62420_c2_g1	-1.18945	0.04884464	
TRINITY_DN62640_c3_g4	-1.43861	0.03771875	XP_010936715
TRINITY_DN62691_c8_g1	-1.28117	0.04517975	
TRINITY_DN62717_c2_g5	-1.22421	0.04884464	
TRINITY_DN62959_c9_g6	-1.33471	0.04217879	
TRINITY_DN63049_c3_g1	-1.19259	0.04884464	
TRINITY_DN63102_c2_g2	-1.5308	0.03517105	XP_010231391
TRINITY_DN63102_c3_g2	-2.40303	0.02352708	XP_010938799
TRINITY_DN63288_c0_g1	-1.64343	0.03165984	XP_008790194
TRINITY_DN63326_c3_g1	-1.91629	0.02804159	
TRINITY_DN63364_c6_g7	-1.44969	0.03722526	
TRINITY_DN63365_c4_g2	-1.53338	0.03517105	
TRINITY_DN63399_c1_g1	-1.3156	0.04319368	
TRINITY_DN63411_c4_g1	-1.64632	0.03156616	
TRINITY_DN63417_c1_g2	-1.46339	0.03662772	XP_010935256
TRINITY_DN63427_c7_g3	-1.94792	0.02750013	

TRINITY_DN63462_c9_g5	-1.22089	0.04884464	XP_009399299
TRINITY_DN63477_c2_g1	-2.0269	0.02622873	
TRINITY_DN63592_c0_g1	-1.20353	0.04884464	XP_008783323
TRINITY_DN63608_c0_g1	-1.29756	0.04414906	XP_010911323
TRINITY_DN63624_c0_g1	-1.34341	0.0416851	XP_008775581
TRINITY_DN63748_c0_g1	-2.3091	0.02352708	XP_008786698
TRINITY_DN64074_c0_g1	-1.23806	0.04803037	XP_010925278
TRINITY_DN64104_c0_g1	-1.5966	0.03304053	XP_008784795
TRINITY_DN64219_c0_g1	-2.60852	0.02352708	XP_010941086
TRINITY_DN64231_c0_g1	-1.3078	0.04361105	XP_009417196
TRINITY_DN64319_c0_g1	-1.32946	0.04250063	XP_010905906
TRINITY_DN64353_c0_g1	-1.18898	0.04884464	XP_009414262
TRINITY_DN64472_c0_g1	-2.38664	0.02352708	
TRINITY_DN64670_c0_g1	-1.8755	0.02871927	XP_008791094
TRINITY_DN64723_c0_g1	-1.75208	0.031045	XP_006357534
TRINITY_DN64852_c0_g3	-2.41778	0.02352708	XP_008788927
TRINITY_DN65078_c0_g1	-1.51094	0.03517105	XP_009386835
TRINITY_DN65219_c0_g1	-1.25961	0.04658698	XP_010934414
TRINITY_DN65240_c0_g1	-1.53892	0.03502336	XP_010918594
TRINITY_DN66089_c0_g1	-1.8252	0.02962	EKV05924
TRINITY_DN70661_c0_g1	-1.41275	0.03885234	XP_008798471
TRINITY_DN70694_c1_g1	-1.47491	0.03613062	XP_010935461
TRINITY_DN70748_c0_g1	-1.97039	0.02713181	ABC55715
TRINITY_DN70758_c0_g1	-1.24757	0.04734445	XP_004241838
TRINITY_DN71154_c0_g1	-2.6704	0.02352708	XP_008808813
TRINITY_DN71159_c0_g1	-1.52233	0.03517105	XP_010919189
TRINITY_DN71483_c0_g1	-1.27247	0.04575487	ADZ76153
TRINITY_DN71853_c0_g1	-1.21685	0.04884464	XP_010929567
TRINITY_DN72430_c0_g1	-1.48773	0.03556861	XP_010915682
TRINITY_DN73755_c0_g1	-1.75663	0.03092636	XP_008801065
TRINITY_DN745_c0_g1	-2.1936	0.02476709	
TRINITY_DN74966_c0_g1	-1.84183	0.02927122	
TRINITY_DN7627_c0_g1	-1.46862	0.03640031	XP_010919331
TRINITY_DN7643_c0_g1	-1.33685	0.04205445	XP_008800087
TRINITY_DN7758_c0_g1	-1.66013	0.03130923	XP_010932283
TRINITY_DN77952_c0_g1	-1.35611	0.04098093	AAB67883
TRINITY_DN77957_c0_g1	-1.56022	0.03426905	BAF36563
TRINITY_DN77971_c0_g2	-1.30424	0.04379499	XP_010931749

TRINITY_DN78005_c0_g1	-1.3287	0.04254476	XP_008813119
TRINITY_DN78011_c0_g1	-1.27962	0.04527867	XP_008783178
TRINITY_DN78302_c0_g1	-1.365	0.04049477	XP_009383460
TRINITY_DN78336_c0_g1	-1.1995	0.04884464	XP_010911198
TRINITY_DN78409_c0_g1	-2.14847	0.02535368	ACQ41837
TRINITY_DN78455_c0_g1	-3.68741	0.02331078	XP_010242173
TRINITY_DN78646_c0_g1	-1.4591	0.03681407	XP_010940051
TRINITY_DN78650_c0_g1	-1.57295	0.03384281	XP_008808743
TRINITY_DN78917_c0_g1	-1.84747	0.02914785	XP_008790305
TRINITY_DN79200_c0_g1	-1.53573	0.03514729	ABZ80408
TRINITY_DN7956_c0_g1	-1.42119	0.03853591	XP_009381090
TRINITY_DN8254_c0_g2	-1.49172	0.03540237	XP_009415734
TRINITY_DN85344_c0_g1	-1.88203	0.0285769	XP_010922265
TRINITY_DN85510_c0_g1	-1.94139	0.0276204	XP_008792681
TRINITY_DN85725_c0_g1	-2.01302	0.02647361	
TRINITY_DN85976_c0_g1	-1.58022	0.03359303	XP_002455523
TRINITY_DN86449_c0_g1	-2.15313	0.02528958	XP_009612669
TRINITY_DN8678_c0_g3	-1.28449	0.04495969	XP_010916637
TRINITY_DN86825_c0_g1	-1.43845	0.0377257	
TRINITY_DN87932_c0_g1	-1.61146	0.03266074	XP_010941635
TRINITY_DN92596_c0_g1	-1.79554	0.03016905	XP_010928510
TRINITY_DN92676_c0_g1	-3.96668	0.02252261	XP_008778500
TRINITY_DN92708_c0_g1	-1.3471	0.04149149	EMS65000
TRINITY_DN93496_c0_g2	-1.58774	0.03334177	XP_010934070
TRINITY_DN93843_c0_g1	-1.22608	0.04884464	XP_008801652
TRINITY_DN93900_c0_g2	-1.37769	0.04012973	XP_008777030
TRINITY_DN97690_c0_g1	-2.1609	0.02518224	
TRINITY_DN99462_c0_g1	-1.38517	0.03978401	XP_008784606
TRINITY_DN99566_c0_g1	-1.31468	0.04323496	XP_009408498
TRINITY_DN99748_c0_g1	-1.42894	0.03819301	
