# Supplementary material for: Comparative transcriptome analysis of Gastrodia elata (Orchidaceae) in response to fungus symbiosis to identify gastrodin biosynthesis-related genes
Source: BMC Genomics. 2016 Mar 9;17:212. doi: 10.1186/s12864-016-2508-6 (PMC4784368; doi:10.1186/s12864-016-2508-6)
Supplement: Additional file 10: Table S7. — Mapping of KEGG biological pathways for up-regulated (log2-FC ≥ 1, q-value < 0.05, TMM-normalized FPKM > 10) unigenes from juvenile tuber of G. elata compared to vegetative propagation corm of G. elata.. (PDF 131 kb) [file 12864_2016_2508_MOESM10_ESM.pdf]

Additional file 10: Table S7. Mapping of KEGG biological pathways for up-regulated (log2-FC ≥ 1, q-value < 0.05, TMM-normalized FPKM > 10) unigenes from juvenile tuber of *G. elata* compared to vegetative propagation corm of *G. elata*.

Pathway category	Pathway	Number of genes
#Metabolism		
##Global and overview maps		
	Carbon metabolism	2
	Degradation of aromatic compounds	1
	Fatty acid metabolism	1
	Biosynthesis of amino acids	1
##Carbohydrate metabolism		
	Starch and sucrose metabolism	7
	Pyruvate metabolism	5
	Pentose and glucuronate interconversions	4
	Glycolysis / Gluconeogenesis	3
	Amino sugar and nucleotide sugar metabolism	1
	Glyoxylate and dicarboxylate metabolism	1
	Citrate cycle (TCA cycle)	1
	Ascorbate and aldarate metabolism	1
	Propanoate metabolism	1
	Fructose and mannose metabolism	1
##Energy metabolism		
	Nitrogen metabolism	1
	Sulfur metabolism	1
	Carbon fixation in photosynthetic organisms	1
##Lipid metabolism		
	Cutin, suberine and wax biosynthesis	3
	Fatty acid degradation	2
	Glycerophospholipid metabolism	2
	Steroid biosynthesis	1
	Glycerolipid metabolism	1
	Fatty acid elongation	1
	Biosynthesis of unsaturated fatty acids	1
	Ether lipid metabolism	1
	Linoleic acid metabolism	1
	Fatty acid biosynthesis	1

	Steroid hormone biosynthesis	1
##Nucleotide metabolism		
	Purine metabolism	2
	Pyrimidine metabolism	2
##Amino acid metabolism		
	Phenylalanine metabolism	8
	Cysteine and methionine metabolism	2
	Alanine, aspartate and glutamate metabolism	2
	Lysine degradation	1
	Glycine, serine and threonine metabolism	1
	Valine, leucine and isoleucine degradation	1
	Tryptophan metabolism	1
	Histidine metabolism	1
	Arginine and proline metabolism	1
##Metabolism of other amino acids		
	Glutathione metabolism	3
	Cyanoamino acid metabolism	3
	beta-Alanine metabolism	1
##Glycan biosynthesis and metabolism		
	Other glycan degradation	1
##Metabolism of cofactors and vitamins		
	Ubiquinone and other terpenoid-quinone biosynthesis	3
##Metabolism of terpenoids and polyketides		
	Diterpenoid biosynthesis	1
	Terpenoid backbone biosynthesis	1
	Limonene and pinene degradation	1
##Biosynthesis of other secondary metabolites		
	Phenylpropanoid biosynthesis	12
	Stilbenoid, diarylheptanoid and gingerol biosynthesis	2
	Flavonoid biosynthesis	2
##Xenobiotics biodegradation and metabolism		
	Drug metabolism - cytochrome P450	2
	Metabolism of xenobiotics by cytochrome P450	2
	Chloroalkane and chloroalkene degradation	1

#Genetic Information Processing		
##Transcription		
	Spliceosome	5
	RNA polymerase	1
##Translation		
	Ribosome	4
	RNA transport	2
##Folding, sorting and degradation		
	Protein processing in endoplasmic reticulum	12
	Proteasome	1
	Ubiquitin mediated proteolysis	1
	RNA degradation	1
	Protein export	1
#Environmental Information Processing		
##Signal transduction		
	Plant hormone signal transduction	4
	MAPK signaling pathway	3
	Two-component system	1
	PI3K-Akt signaling pathway	1
	cAMP signaling pathway	1
	MAPK signaling pathway - yeast	1
	Ras signaling pathway	1
	FoxO signaling pathway	1
	Wnt signaling pathway	1
	Sphingolipid signaling pathway	1
	Rap1 signaling pathway	1
	AMPK signaling pathway	1
#Cellular Processes		
##Transport and catabolism		
	Endocytosis	4
	Peroxisome	3
	Phagosome	1
	Regulation of autophagy	1
