# Supplementary material for: Comparative transcriptome analysis of Gastrodia elata (Orchidaceae) in response to fungus symbiosis to identify gastrodin biosynthesis-related genes
Source: BMC Genomics. 2016 Mar 9;17:212. doi: 10.1186/s12864-016-2508-6 (PMC4784368; doi:10.1186/s12864-016-2508-6)
Supplement: Additional file 11: Table S8. — Mapping of KEGG biological pathways for down-regulated ((log2-FC ≤ -1, q-value < 0.05, TMM-normalized FPKM > 10) unigenes from juvenile tuber of G. elata compared to vegetative propagation corm of G. elata. (PDF 130 kb) [file 12864_2016_2508_MOESM11_ESM.pdf]

Additional file 11: Table S8 Mapping of KEGG biological pathways for down-regulated ((log2-FC ≤ -1, *q*-value < 0.05, TMM-normalized FPKM > 10) unigenes from juvenile tuber of *G. elata* compared to vegetative propagation corm of *G. elata*.

Pathway category	Pathway	Number of genes
#Metabolism		
##Global and overview maps		
	Carbon metabolism	5
	Fatty acid metabolism	3
	Biosynthesis of amino acids	3
	2-Oxocarboxylic acid metabolism	1
##Carbohydrate metabolism		
	Starch and sucrose metabolism	6
	Amino sugar and nucleotide sugar metabolism	4
	Pentose and glucuronate interconversions	3
	Fructose and mannose metabolism	2
	Glycolysis / Gluconeogenesis	2
	Ascorbate and aldarate metabolism	2
	Pyruvate metabolism	2
	Galactose metabolism	2
	Citrate cycle (TCA cycle)	1
	Inositol phosphate metabolism	1
	Glyoxylate and dicarboxylate metabolism	1
	C5-Branched dibasic acid metabolism	1
##Energy metabolism		
	Oxidative phosphorylation	3
	Carbon fixation in photosynthetic organisms	2
	Carbon fixation pathways in prokaryotes	1
	Sulfur metabolism	1
	Methane metabolism	1
	Nitrogen metabolism	1
##Lipid metabolism		
	Biosynthesis of unsaturated fatty acids	3
	Glycerophospholipid metabolism	2
	alpha-Linolenic acid metabolism	2
	Fatty acid degradation	2
	Fatty acid elongation	2

	Sphingolipid metabolism	1
	Glycerolipid metabolism	1
	Cutin, suberine and wax biosynthesis	1
##Nucleotide metabolism		
	Pyrimidine metabolism	1
	Purine metabolism	1
##Amino acid metabolism		
	Phenylalanine metabolism	7
	Glycine, serine and threonine metabolism	5
	Tyrosine metabolism	4
	Tryptophan metabolism	1
	Valine, leucine and isoleucine biosynthesis	1
	Alanine, aspartate and glutamate metabolism	1
	Cysteine and methionine metabolism	1
##Metabolism of other amino acids		
	beta-Alanine metabolism	3
	Cyanoamino acid metabolism	2
	Glutathione metabolism	1
##Metabolism of cofactors and vitamins		
	Ubiquinone and other terpenoid-quinone biosynthesis	2
	Vitamin B6 metabolism	2
##Metabolism of terpenoids and polyketides		
	Zeatin biosynthesis	1
##Biosynthesis of other secondary metabolites		
	Phenylpropanoid biosynthesis	10
	Isoquinoline alkaloid biosynthesis	3
	Tropane, piperidine and pyridine alkaloid biosynthesis	3
	Flavonoid biosynthesis	2
	Stilbenoid, diarylheptanoid and gingerol biosynthesis	1
	Betalain biosynthesis	1
	Butirosin and neomycin biosynthesis	1
	Streptomycin biosynthesis	1
##Xenobiotics biodegradation and metabolism		
	Metabolism of xenobiotics by cytochrome	1

	P450	
	Styrene degradation	1
	Drug metabolism - cytochrome P450	1
#Genetic Information Processing		
##Transcription		
	Spliceosome	2
	RNA polymerase	1
##Translation		
	mRNA surveillance pathway	2
	Ribosome	1
	RNA transport	1
##Folding, sorting and degradation		
	SNARE interactions in vesicular transport	2
	Ubiquitin mediated proteolysis	2
	Protein processing in endoplasmic reticulum	1
	Proteasome	1
##Replication and repair		
	Fanconi anemia pathway	1
#Environmental Information Processing		
##Signal transduction		
	Plant hormone signal transduction	5
	Phosphatidylinositol signaling system	3
	cAMP signaling pathway	3
	Calcium signaling pathway	3
	Rap1 signaling pathway	2
	FoxO signaling pathway	2
	NF-kappa B signaling pathway	2
	cGMP-PKG signaling pathway	2
	Ras signaling pathway	2
	HIF-1 signaling pathway	1
#Cellular Processes		
##Transport and catabolism		
	Lysosome	5
	Peroxisome	2
	Endocytosis	1
	Regulation of autophagy	1
