# Supplementary material for: Comparative transcriptome analysis of Gastrodia elata (Orchidaceae) in response to fungus symbiosis to identify gastrodin biosynthesis-related genes
Source: BMC Genomics. 2016 Mar 9;17:212. doi: 10.1186/s12864-016-2508-6 (PMC4784368; doi:10.1186/s12864-016-2508-6)
Supplement: Additional file 13: Figure S5. — Vegetative propagation corm of Gastrodia elata fastq sequences checked by FastQC for (a) per base sequence quality and (b) per sequence quality scores. (a): The y-axis on the graph shows the quality scores. The higher the score the better the base call. The background of the graph divides the y axis into very good quality calls (green), calls of reasonable quality (orange), and calls of poor quality (red). The central red line is the median value. The yellow box represents the inter-quartile range (25–75 %). The upper and lower whiskers represent the 10 and 90 % points. The blue line represents the mean quality. (b): The most frequently observed mean quality below 39 is meaning that this equates to a 0.0126 % error rate. (PDF 109 kb) [file 12864_2016_2508_MOESM13_ESM.pdf]

(a)

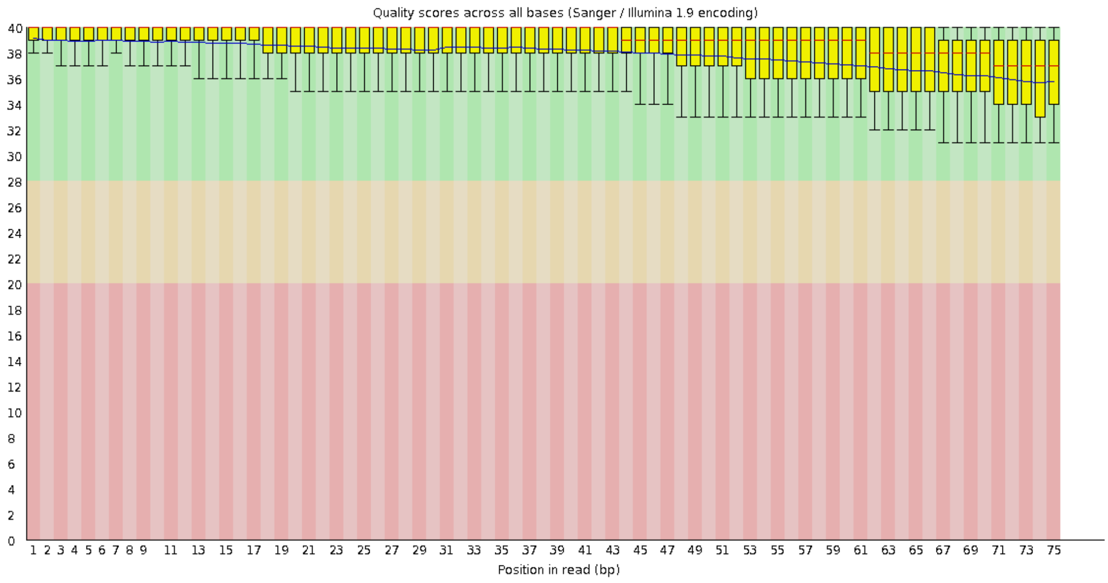

(b)

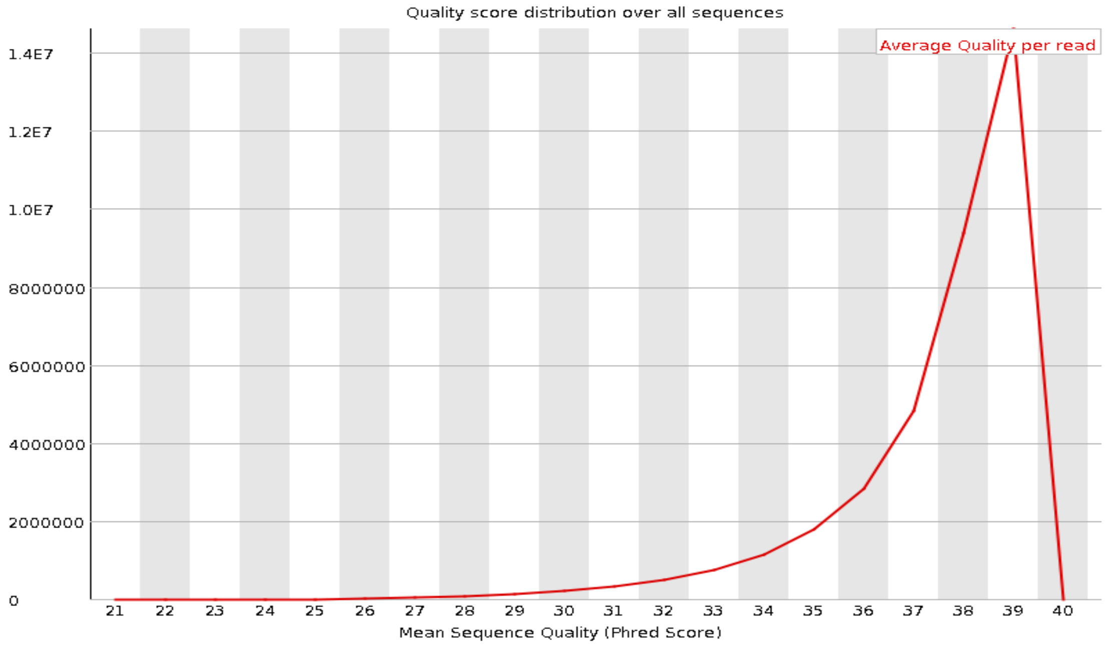

Additional file 13: Figure S5. Vegetative propagation corm of *Gastrodia elata* fastq sequences checked by FastQC for (a) per base sequence quality and (b) per sequence quality scores. (a): The y-axis on the graph shows the quality scores. The higher the score the better the base call. The background of the graph divides the y axis into very good quality calls (green), calls of reasonable quality (orange), and calls of poor quality (red). The central red line is the median value. The yellow box represents the inter-quartile range (25-75%). The upper and lower whiskers represent the 10% and 90% points. The blue line represents the mean quality. (b): The most frequently observed mean quality below 39 is meaning that this equates to a 0.0126% error rate.
