# Supplementary material for: Comparative transcriptome analysis of Gastrodia elata (Orchidaceae) in response to fungus symbiosis to identify gastrodin biosynthesis-related genes
Source: BMC Genomics. 2016 Mar 9;17:212. doi: 10.1186/s12864-016-2508-6 (PMC4784368; doi:10.1186/s12864-016-2508-6)
Supplement: Additional file 15: Table S9. — List of primers used for SMART-RACE and Real-time PCR analysis. (PDF 80 kb) [file 12864_2016_2508_MOESM15_ESM.pdf]

Additional file 15: TableS9. List of primers used for SMART-RACE and Real-time PCR analysis.

Primer name	Sequence 5'-3'	Direction
GlcT-F	TGGAGATCCTATGCGCCAAGAGAGTGTC	sqPCR and qPCR
GlcT-R	TCGATCTCAGGAAGGAGCACAGAGATGG	sqPCR and qPCR
CYP450-F	CGAGATCTTCGCCTCCATCATCCAATCC	sqPCR and qPCR
CYP450-R	TGGTCGCCCGCTCTTGTATCTTGAATCG	sqPCR and qPCR
Ubiquitin-F	ACATTCAGAAGGAGTCGACCCTTCATCTC	sqPCR and qPCR
Ubiquitin-R	GCATTCCAGCCAAGGATCACTGTATTCAC	sqPCR and qPCR
GlcT-F1	TGGTGTTTGGCGCATTGCAGCTATAAACG	SMART-RACE PCR
GlcT-F2	GGACTTAGCAATCAGAGCCACACTTGTTG	SMART-RACE PCR
GlcT-R1	AAAGTCAGATGAACTGAGCTGGGAGTTCC	SMART-RACE PCR
GlcT-R2	GTTATGATGGCTGGGATGTACACTACTCC	SMART-RACE PCR
CYP450-F1	AGCGTAGATCTGAAGCACGAGCTGGA	SMART-RACE PCR
CYP450-F2	CTTTGATGACGTCTCCGCCCTATTCC	SMART-RACE PCR
CYP450-R1	ACCACCATGGCGTTCCAATCGATCTC	SMART-RACE PCR
CYP450-R2	ATGTCGCCAGCGGGTATATCGAACTC	SMART-RACE PCR
